# Design, Synthesis and Biological Evaluation of Biscarbamates as Potential Selective Butyrylcholinesterase Inhibitors for the Treatment of Alzheimer’s Disease

**DOI:** 10.3390/ph15101220

**Published:** 2022-09-30

**Authors:** Ana Matošević, Anamarija Knežević, Antonio Zandona, Nikola Maraković, Zrinka Kovarik, Anita Bosak

**Affiliations:** 1 Biochemistry and Organic Analytical Chemistry Unit, Institute for Medical Research and Occupational Health, HR-10000 Zagreb, Croatia; 2 Division of Organic Chemistry and Biochemistry, Ruđer Bošković Institute, HR-10000 Zagreb, Croatia

**Keywords:** acetylcholinesterase, metal chelating, detailed kinetic study, spontaneous decarbamylation, rate constants, molecular docking

## Abstract

As butyrylcholinesterase (BChE) plays a role in the progression of symptoms and pathophysiology of Alzheimer’s disease (AD), selective inhibition of BChE over acetylcholinesterase (AChE) can represent a promising pathway in treating AD. The carbamate group was chosen as a pharmacophore because the carbamates currently or previously in use for the treatment of AD displayed significant positive effects on cognitive symptoms. Eighteen biscarbamates with different substituents at the carbamoyl and hydroxyaminoethyl chain were synthesized, and their inhibitory potential toward both cholinesterases and inhibition selectivity were determined. The ability of carbamates to cross the blood–brain barrier (BBB) by passive transport, their cytotoxic profile and their ability to chelate biometals were also evaluated. All biscarbamates displayed a time-dependent inhibition with inhibition rate constants within 10^−3^–10^−6^ M^−1^ min^−1^ range for both cholinesterases, with generally higher preference to BChE. For two biscarbamates, it was determined that they should be able to pass the BBB by passive transport, while for five biscarbamates, this ability was slightly limited. Fourteen biscarbamates did not exhibit a cytotoxic effect toward liver, kidney and neuronal cells. In conclusion, considering their high BChE selectivity, non-toxicity, ability to chelate biometals and pass the BBB, compounds **2** and **16** were pointed out as the most promising compounds for the treatment of middle and late stages of AD.

## 1. Introduction

Alzheimer’s disease (AD) is the most common and widespread neurodegenerative disease characterized by memory and judgment ability loss and personality changes [1]. Today, it affects more than 50 million people worldwide and has a tendency to continuously grow as a result of the aging of the world’s population [2]. AD is a multifactorial disease with various pathological features: deficiency of the neurotransmitter acetylcholine (ACh), accumulation of beta-amyloid (Aβ) plaques, hyperphosphorylation of tau protein, overstimulation of *N*-methyl-*D*-aspartate receptor, changes in the homeostasis of biometals, increased MAO-B enzyme activity and oxidative stress [3,4].

Although the multifactorial nature of disease points at the existence of a number of possible targets, the existing treatment for AD is based mainly on increasing the concentration of ACh by inhibiting the action of enzymes responsible for its hydrolysis, acetylcholinesterase (AChE) and butyrylcholinesterase (BChE), or using *N*-methyl-*D*-aspartate (NMDA) receptor antagonists; both directions are directed at the restoration of cognitive functions of patients and alleviating the symptoms of the disease. The new entry in AD treatment is the use of a monoclonal IgG1 antibody [5], aducanumab, which is the only drug aimed to cure or stop the progression of the disease. It exerts its mechanism of action by selectively targeting and binding aggregated soluble oligomers and insoluble fibrils conformations of Aβ plaques to reduce their levels in the brain [5]. Three of the five currently approved drugs for AD treatment are cholinesterases inhibitors. Due to AChE’s crucial role in neurotransmission and consequently the development and progression of AD, drugs aimed at increasing the levels of ACh in the brain were developed primarily as AChE inhibitors. The currently approved [6] cholinesterase inhibitors donepezil and galantamine are selective AChE inhibitors, while carbamate rivastigmine is a non-selective inhibitor of AChE and BChE [7,8].

An important role of the BChE in the regulation of brain ACh levels in late AD [9,10] was pointed out based on findings that, during disease progression, AChE activity decreases to about 33–45% of its normal activity, while BChE activity progressively increases by about 40–90% of its normal activity [8]. Animal studies on rodents have shown that selective inhibition of BChE, with respect to AChE, has a beneficial effect on cognitive abilities of rodents with AD and reduces accumulations of amyloid plaques in their brains [11,12]. Moreover, recent studies have shown that selective inhibition of BChE reduces the occurrence of side effects like tremor, anxiety, hypersalivation and sweating seen with the AChE or nonselective cholinesterase inhibitors currently in use [13]. Consequently, selective inhibition of BChE has evolved into a promising new approach in the treatment of middle and advanced AD.

To date, a number of selective BChE inhibitors with different structural elements have been synthesized and tested. Many of them had tacrine as a structural scaffold (thienothiazines, thiazoles, quinuclidines, benzofuranes, quinolines, etc.), but many other structural scaffolds were also used, as well as hybrids containing galantamine, donepezil and rivastigmine as moieties [14,15,16,17,18,19,20]. However, so far, no drug that is a selective BChE inhibitor has been approved for the treatment of AD.

**Figure 1:**
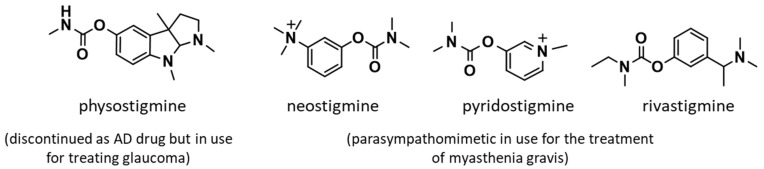
Carbamate based drugs that are or have been used for treatment of AD.

Very promising results were obtained for compounds with a carbamate group as a functional scaffold due to the similarity of mechanism of cholinesterases hydrolysis of carbamates with the mechanism of AChE hydrolysis of its physiological substrate ACh [21,22]; the difference lies in the stability of the carbamylated cholinesterases which decarbamylate more slowly than acetylated AChE [22]. Several drugs which are currently in use or had been used for the treatment of AD were carbamates (Figure 1) [23] of which rivastigmine is a non-selective cholinesterase inhibitor currently in use [24,25]. Physostigmine was the first carbamate clinically used as a cholinesterase inhibitor, but its use today is reduced due to its poor bioavailability and adverse side effects [8]. Many physostigmine derivatives entered clinical trials: development of eptastigmine as a drug was stopped due the development of neutropenia in patients [26]; phenserine tartrate entered phase III of clinical trials, but its further development failed due to certain concerns regarding the documentation of its clinical trials [27,28,29]; cymserine inhibits BChE 15 times faster than AChE, but its development has been discontinued due to unacceptable side effects caused by its toxic metabolite eseroline [30,31,32]. After cymserine, several cymserine derivatives have been synthesized with greater selectivity for BChE than cymserine, and several of them were tested in animals demonstrating the ability to increase brain ACh levels and produce nootropic effects, as well as reducing levels of amyloid precursor protein and Aβ [30], but so far only bisnorcymserine has entered phase I clinical trials for the treatment of AD [33].

Bambuterol, a biscarbamate ester of terbutaline, a drug for the treatment of asthma, has been shown to be highly selective to BChE by inhibiting it 20,000 times faster than AChE [22,34]. Considering the fact that it is already in use as a drug without side effects related to the cholinergic system, bambuterol stood out as a promising candidate for repurposing into an agent for AD treatment. This was additionally supported by the fact that the monocarbamate derivative of bambuterol, released after the decarbamylation of cholinesterase inhibited by bambuterol, also inhibits cholinesterases [22] which enables the prolonged action of bambuterol as an AD drug targeting BChE. However, repurposing bambuterol as an AD drug failed due to its poor ability to cross the blood–brain barrier (BBB) [35]. Wu and colleagues synthesized a series of bambuterol analogues with different amino parts of the molecule with the aim to achieve a potent and BChE selective carbamate-based inhibitor where derivatives with 2-methylbutan as a substituent on the amine were pointed out as the most promising for further evaluation as AD drugs [36,37].

In this paper, we synthesized eighteen new biscarbamates with different substituents at the carbamoyl and dihydroxyaminoethyl part of the molecule with carbamate moieties in the meta position on the benzene ring. Their inhibition potency and selectivity toward human BChE or AChE were determined and analyzed using molecular modelling. As newly synthesized biscarbamates were synthesized with the aim of using them as central nervous system (CNS) active drugs, we evaluated their physicochemical properties and estimated their ability to cross the blood–brain barrier (BBB) by passive transport and tested their cytotoxicity on cells that represent models of individual organs. Additionally, the ability of tested biscarbamates to chelate biometals (Zn, Cu and Fe) was evaluated as several studies have shown that dyshomeostasis of biometals may contribute to AD pathology.

## 2. Results and Discussion

### 2.1. Design and Synthesis of Compounds

**Scheme 1:**
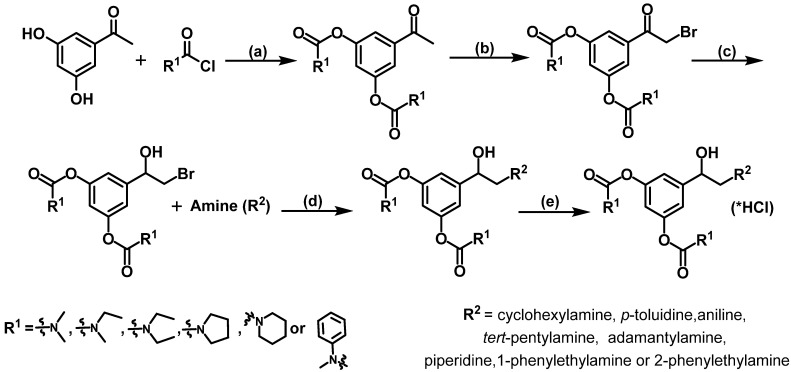
The synthetic route of the designed biscarbamates. Reagents and conditions: (**a**) triethylamine, 4-(dimethylamino) pyridine, 1,2-dichloroethane, 60–65 °C, t = 24 h; (**b**) CuBr_2_, ethyl acetate:chloroform = 1:1, 60 °C, t = 5 h; (**c**) NaBH_4_, MeOH, CH_2_Cl_2_, t = 3 h; (**d**) isopropanol, 60–80 °C, t = 24 h; (**e**) HCl in isopropanol, 60 °C.

**Figure 2:**
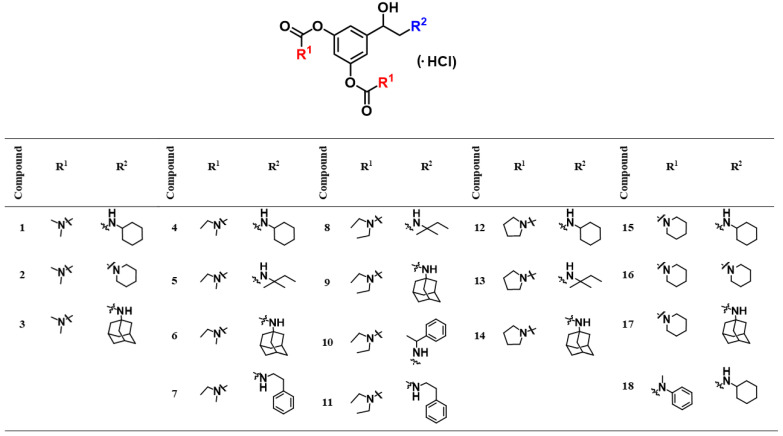
General structure of the synthesized biscarbamates.

Biscarbamates were designed using bambuterol as a structural scaffold retaining the meta-position of carbamate groups on the benzene ring. The choice of meta disposition of carbamate groups on benzene was rationalized based on our previous study which determined that the inhibition potency and selectivity of biscarbamates towards BChE over AChE is dictated by the disposition of carbamate groups on the benzene ring, where a meta-position was preferred over an ortho-position [37,38]. In this study, we attempted to explore the impact of the carbamate group size and the size of the terminal amino group on inhibition potency and selectivity of biscarbamates to BChE over AChE. Eighteen dimethyl, ethylmethyl, diethyl, 1-pyrrolidine, 1-piperidine and methylphenyl biscarbamates with various alkyl, cyclic or aromatic terminal amines (Figure 2) were synthesized as racemates starting from commercially available 3,5-dihydoxyacetophenone, applying a slightly modified protocol described previously (Scheme 1) [36]. Briefly, the five-step synthesis included: carbamylation of 3,5-dihydroxyacetophenone using carbamoyl chloride; the introduction of bromine into the α-position in relation to the biscarbamate keto group; reduction of α-bromoketone to β-bromoalcohol; addition of the corresponding amine on β-bromoalcohol; and the hydrochlorination of biscarbamate β-aminoalcohol. A more detailed description of the synthetic route is available in Supporting Information I.

All of the synthesized biscarbamates were obtained at a 10–70% yield as oils and had a chemical purity ≥95%. Fourteen out of eighteen compounds were new compounds. Structures of the prepared compounds were confirmed with NMR and HRMS spectra.

### 2.2. Kinetic Studies

**Figure 3:**
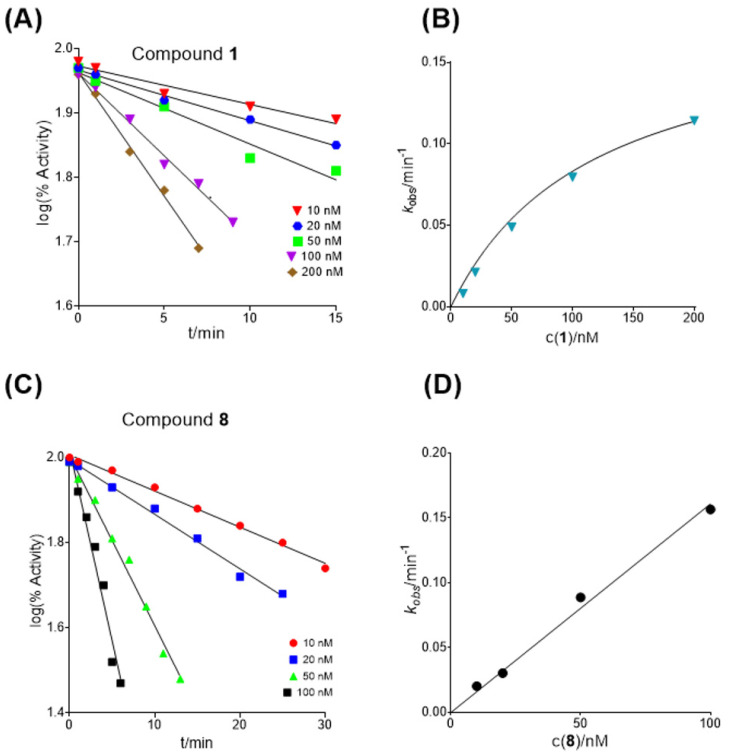
Representative experiment of inhibition of BChE by compounds **1** and **8**. Points in panels (**A**,**C**) indicate the logarithm of residual activity, while the slope of the lines determines the observed first-order rate constant *k*_obs_. On panels (**B**,**D**), constants *k*_obs_ were plotted as a function of biscarbamate concentration wherefrom the second-order rate constant, *k*_i_, was calculated. Compound **1** displayed a nonlinear dependence of the *k*_obs_ on biscarbamate concentration (panel (**B**)) allowing determination of the maximal first-order rate constant of carbamylation, *k*_max_, and dissociation constant of Michaelis type of complex between enzyme and inhibitor, *K*_i_, while *k*_i_ is defined as their ratio. For biscarbamate **8**, k_obs_ was a linear function of biscarbamate concentration (panel (**D**)), and the slope defined the *k*_i_.

The ability of biscarbamates to reduce the activity of human BChE and AChE was tested for all of the synthesized compounds. All of the compounds displayed a time-dependent inhibition of both, BChE and AChE, which indicated that, during the binding of the inhibitor to the active site of both enzymes, a covalent bond between the catalytic serine and the carbamate group of biscarbamate was formed. The experiments were designed so that the maximum preincubation time of the enzyme with carbamates was 30 min to assure that during that period no spontaneous decarbamylation would occur [39,40,41]. Inhibition of both cholinesterases by all of the tested carbamates followed first-order kinetics at any given inhibitor concentration (Figure 3A,C). The inhibition potency of the compounds was expressed by the overall inhibition rate constant (*k*_i_) that represents the first step in biscarbamate hydrolysis (enzyme inhibition scheme in Section 4) determined from the relation between the first-order rate constant (*k*_obs_) and inhibitor concentration. For the majority of compounds, relation of *k*_obs_ vs. [CC′] deviated from linearity (Figure 3B), allowing determination of intrinsic kinetic constants: the maximal first-order rate constant of carbamylation, *k*_max_, and dissociation constant, *K*_i_ (Table 1). For the rest of the compounds, the *k*_obs_ constant was a linear function of biscarbamate concentration (Figure 3D).

#### 2.2.1. Inhibition of Butyrylcholinesterase

The tested carbamates were divided into six groups according to the substituents on carbamoyl nitrogen (Table 1). Within a particular group, the compounds differed in the size of the hydroxyaminoethyl chain attached to the benzene ring.

All of the compounds inhibited BChE with *k*_i_ constants in the range 0.0144–38.0·10^6^ M^−1^ min^−1^, which makes these compounds fast or very fast BChE inhibitors. The fastest inhibition was obtained by compound **16** with piperidine in the carbamoyl and hydroxyaminoethyl chain of the compound, being almost a 10 times faster inhibitor than bambuterol. Compounds **6** and **13** were equally potent inhibitors and ten compounds (**2**, **4**, **5**, **8**, **9**, **12**, **14**, **15**, **17** and **18**) had an about two times lower inhibition potency of BChE than bambuterol. The tested carbamates were divided into six groups according to the substituents on the carbamoyl nitrogen. Within a particular group, the compounds differed in the size of the hydroxyaminoethyl chain attached to the benzene ring. Generally, no clear correlation between the length and/or the size of groups on the carbamoyl nitrogen and inhibition potency of compounds was detected It can be said that a lower inhibition potency can be expected for compounds with an aromatic amine on the hydroxyaminoethyl chain since the lowest inhibition potency, about 2400 times lower than that of compound **16**, was determined for compounds **10** and **11** with phenyl as a substituent on the hydroxyaminoethyl chain combined with alkyl groups on carbamoyl nitrogen. But, when more detailed structure-activity analysis was performed, analyzing the influence of different substituents on carbamoyl nitrogen with the aniline on the hydroxyaminoethyl chain, it could be seen that replacement of aliphatic substituents does not affect the inhibition potency of the compounds; the increase in inhibition potency occurred when piperidine was used. That observation is in accordance with the structure-activity analysis of the same class of compounds reported earlier by Wu et al., where it was demonstrated that replacing dimethyl groups with ethylmethyl on the carbamoyl part of molecules did not affect the inhibition potency of the biscarbamates [36].

The BChE active site did not discriminate binding of structural isomers **10** and **11**, which differed in the substituent on the hydroxyaminoethyl chain.

Detailed inhibition studies revealed that fifteen out of eighteen biscarbamates displayed a nonlinear dependence of the first-order rate constant (*k*_obs_) on biscarbamate concentration allowing for the determination of the maximal first-order rate constant of carbamylation, *k*_max_, and dissociation constant of the enzyme–carbamate Michaelis type of complex, *K*_i_. For three biscarbamates **8**, **10** and **11**, *k*_obs_ was a linear function of biscarbamate concentration. As the reciprocal value of *K*_i_ represents the affinity of the enzyme to the compounds, it seems that the low inhibition potency of the compound **3** can be a result of BChE’s low affinity to that compound. In line with that, the high inhibition potency of compounds **2**, **5** and **16** can be due to BChE’s high affinity towards them. On the other hand, though generally *k*_max_ had a lower impact on the value of *k*_i_ compared to *K*_i_, higher impact can be considered for compounds **4**, **9**, **13** and **14** with higher *k*_max_ values than the rest of the compounds.

The inhibition potency of the tested compounds was compared to the inhibition potency of rivastigmine as an FDA-approved drug for the treatment of ADs currently in use. The majority of tested carbamates were equally or more potent (even up to 690 times) BChE inhibitors than rivastigmine. Compounds **3**, **10** and **11** were the only whose inhibition potency was about three times lower than the inhibition potency of rivastigmine. The *k*_i_ constant of rivastigmine determined in the study was about two-three times lower than that determined earlier [24]. The tested compounds inhibited human BChE at generally lower concentrations than dimethyl, and methylethyl biscarbamates, synthesized by Wu, inhibited horse BChE [36]. The inhibition rate constant determined for compound **5** corresponded to the value determine earlier by Wu [36].

#### 2.2.2. Inhibition of Acetylcholinesterase

The tested carbamates inhibited AChE with *k*_i_ constants in the range of (0.00330–4.56) 10^6^ M^−1^ min^−1^. The fastest inhibition was obtained by compound **8** with diethyl groups in the carbamoyl and *tert*-dimethylethyl substituent in the hydroxyaminoethyl chain of the compound. The tested carbamates were 15 to 20.727 times more potent AChE inhibitors than bambuterol [34,42]. Besides compound **8**, compounds **4**, **6** and **9** inhibited AChE with inhibition rate constants within the 10^6^ M^−1^ min^−1^ range, compound **17** in the 10^5^ M^−1^ min^−1^ range, eight compounds in 10^4^ and five compounds below the 10^4^ M^−1^ min^−1^ range. No relationship between inhibition potency and structure of tested compounds was observed. The AChE active site did not discriminate binding of structural isomers **10** and **11**.

Detailed inhibition studies revealed that twelve compounds displayed a nonlinear dependence of the first-order rate constant (*k*_obs_) on biscarbamate concentration, while for the rest of the carbamates, *k*_obs_ was a linear function of biscarbamate concentration. According to the values of *K*_i_ constants, it seems that the inhibition potency of the tested carbamates can be related to the AChE’s affinity to the compounds; high inhibition potency of the compounds **8**, **4**, **6** and **9** is due to AChE’s high affinity to these compounds. The inhibitory potential of the rest of the tested carbamates followed changes in the affinity of AChE towards the carbamates.

The majority of the tested carbamates were equally or more potent (up to 2054 times) AChE inhibitors than rivastigmine. The *k*_i_ constant determined for rivastigmine in the study corresponded to that determined earlier [24]. Compounds **4**, **6**, **8** and **9** were about equally potent AChE inhibitors as physostigmine, a carbamate compound that was used as an FDA-approved drug for the treatment of AD, and neostigmine, a carbamate in use for the treatment of myasthenia gravis [43,44,45].

#### 2.2.3. Selectivity of Inhibition

The inhibition selectivity of the tested biscarbamates was evaluated as the ratio of overall inhibition rate constants determined for BChE and AChE (Table 1). Generally, the tested carbamates were selective BChE inhibitors. The most selective were compounds **13** and **16**, which inhibited BChE 1288 and 1087 times faster than AChE, respectively. Seven more compounds were more than 50 times selective to BChE than to AChE. The BChE/AChE selectivity ratio of the compound **17** was 12, and that of the rest of the compounds was lower than 10, whereas compounds **4** and **8** were up to 2.5 times more selective to AChE than to BChE or non-selective as compound **9**. Regarding the groups on carbamoyl nitrogen, groups with cyclic amines as the amine part of the carbamate were more BChE selective than groups having alkyl amines. The lowest selectivity was that of compounds with a diethyl group on carbamoyl nitrogen regardless of the substituent on the hydroxyaminoethyl chain. Generally, none of the compounds reached the selectivity of bambuterol. Selectivity of compounds **13**, **14**, **15**, **16** and **18** was up to 51 times higher than selectivity of rivastigmine [24].

### 2.3. Docking Analysis

**Figure 4:**
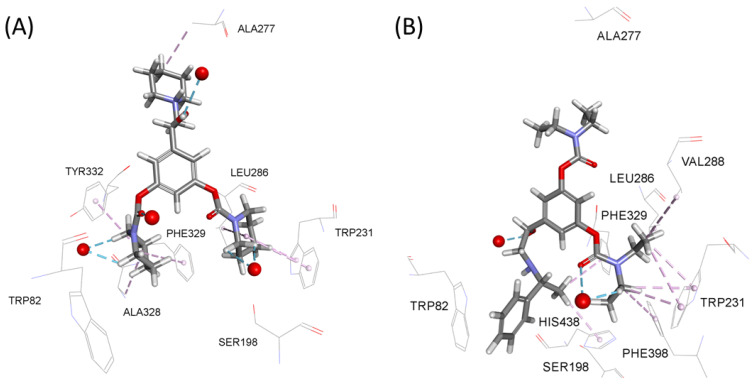
The active site of the model complex between BChE and **16** (**A**) or **10** (**B**). Dashed lines represent different types of non-bonding intermolecular interactions. Red spheres represent conserved water molecules. For clarity, only water molecules predicted to be engaged in non-bonding interactions are shown.

Docking studies were conducted to gain insight into the structural features governing the observed differences in inhibition potency among the tested compounds. As rate of carbamylation of BChE depends on the entry of carbamate into the BChE binding site and the formation of the reversible BChE-carbamate complex close to the catalytic serine, we analyzed non-bonding interactions contributing to the stabilization of BChE-carbamate complexes and positioning of carbamates in a way that makes it susceptible to the nucleophilic attack of catalytic serine oxygen (O_Ser198_) on carbonyl group (C=O) and facilitates the release of the carbamate leaving group. A flexible docking protocol using the crystal structure of free BChE superimposed onto a network of conserved water molecules as the receptor structure was utilized to predict the binding mode of the tested compounds prior to carbamylation, i.e., the BChE-carbamate Michaelis-like complex. The protocol was applied as reported previously [45]. The predicted model complexes between the selected compounds and free BChE were analyzed for non-bonding interactions. Figure 4A illustrates the active site of the model complex between the most active carbamate from the tested series, **16**, and free BChE. The predicted binding mode of carbamate **16** in the BChE active site showed that it occupied active site gorge with an orientation that fulfils prerequisites for the carbamylation reaction to occur once the carbamate slides deeper into the active site. Namely, both of its carbamate groups are oriented towards the bottom of the active site approximately occupying the choline-binding pocket and acyl-binding pocket, respectively, while the leaving group is oriented towards the gorge entrance. Carbamate **16** is predicted to engage in multiple non-bonding interactions with neighboring residues outlining the BChE active site gorge and conserved water molecules. These include water hydrogen bonds and hydrophobic-alkyl and hydrophobic-π-alkyl interactions with residues from the choline-binding pocket (Ala328, Phe329) and acyl-binding pocket (Trp231, Leu286) (the full list of predicted non-bonding interactions for all compounds can be found in Supplementary Materials). The predicted binding poses are a satisfactory illustration of one of the many transient complexes existing in the dynamic process of carbamate advancement towards the bottom of the active site gorge.

On the other hand, the predicted binding mode of one of the least active carbamates from the series, **10** (Figure 4B), shows it is almost equally involved in non-bonding interactions with neighboring residues and conserved water molecules. These include water hydrogen bonds and hydrophobic-alkyl and hydrophobic-π-alkyl interactions predominantly with residues from the acyl-binding pocket (Trp231, Leu286, Val288). However, its predicted orientation reveals one significant difference. Namely, only one of its two carbamate groups is directed towards the bottom of the active site (the other is directed to the gorge entrance) reducing the probability of a successful carbamylation reaction, which could be the reason for its low activity. Moreover, such a spatial orientation of benzene substituents in **10** inside the BChE active site leads to the formation of an intramolecular carbon hydrogen bond between carbamate carbonyl oxygen and hydrogen attached to the carbon adjacent to cyclohexane moiety. This interaction locks-up carbamate in an unfavorable position for a carbamylation reaction, adding to its low activity.

### 2.4. Decarbamylation Process

Inhibition by biscarbamates occurs by the formation of a covalent bond between the carbamate group of the compound and the catalytic serine of the enzyme during which a carbamylated enzyme is formed. The decomposition of carbamylated enzyme is spontaneous and occurs by the action of water, where a free enzyme and monocarbamate were released. The recovery of enzyme activity, i.e., a spontaneous reactivation named rate of decarbamylation, is characterized by the first-order rate constant (decarbamylation rate constant; *k*_decarb_) determined by following the spontaneous recovery of enzyme activity in time. The rate of decarbamylation depends on the substituents on carbamoyl nitrogen: rate of spontaneous decarbamylation of an enzyme inhibited by *N*-unsubstituted carbamates is faster than by *N*-substituted carbamates, and it also depends on the length and branching of substituents on the nitrogen atom [46]. In terms of drug development, the rate of spontaneous decarbamylation is important because it can be used for the estimation of the time of a drug’s action.

Decarbamylation rate constants were determined for BChE and AChE, for all of the tested compounds (Table S1 in Supplementary Materials). For BChE, *k*_decarb_ ranged from 0.0720 to 0.192 h^−1^ (*k*_decarb_ = 0.136 ± 0.013 h^−1^), and 0.138 to 0.222 h^−1^ (0.173 ± 0.014 h^−1^) for AChE. In general, the rate of decarbamylation of BChE or AChE was very similar for all of the tested compounds, regardless of the substituents on the carbamoyl nitrogen or hydroxyaminoethyl chain. The values of *k*_decarb_ constants were consistent with the literature data for *N*-disubstituted carbamates [40,46,47,48,49]. Moreover, the rates of decarbamylation of BChE and AChE were similar, except for reactivation of enzymes carbamylated with cyclic carbamates, where decarbamylation of BChE was slightly faster than that of AChE.

### 2.5. Metal Chelating Ability

**Figure 5:**
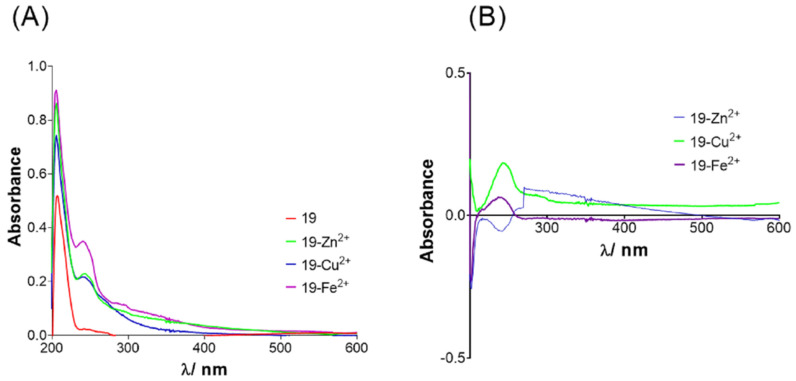
(**A**) The UV–VIS spectra of biscarbamate **13** alone (red) and in the presence of Zn^2+^ (green), Cu^2+^ (blue) and Fe^2+^ (purple) recorded after 30 min of incubation. (**B**) The differential spectra of **13**−Zn^2+^ (green), **13**−Cu^2+^ (blue) and **13**−Fe^2+^ complexes (purple).

All of the synthesized biscarbamates were tested for the ability to chelate biometal ions Fe^2+^, Zn^2+^ and Cu^2+^. The absorbance spectra of biscarbamates and metal mixture were recorded following 1, 30 and 60 min after mixing them and all showed changes in the spectra compared to the spectra of biscarbamates, which has been previously reported as evidence of formation of metal-compound complex: changes in absorbance intensity, bathochromic shifts of peaks or appearance of additional peaks [50,51]. Spectra recorded after a 30 min incubation period were chosen for differential spectra analysis, as after 30 min the spectra of biscarbamate-metal mixture did not change with time. Differential spectra (Figure 5) confirmed the existence of a biscarbamate-metal complex, i.e., the biscarbamate chelating ability.

**Figure 6:**
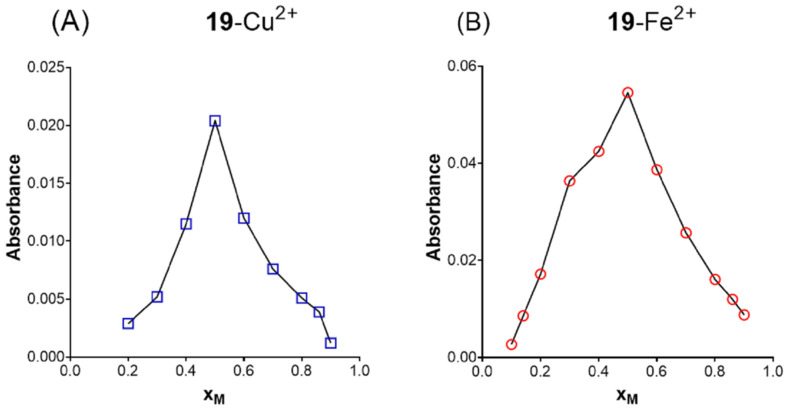
Determination of the stoichiometry of (**A**) **13**–Cu^2+^ complex and (**B**) **13**–Fe^2+^ complex with a breakpoint observed at biscarbamate:metal ratio = 1:1.

Furthermore, for the most selective BChE inhibitors **1**, **2**, **4**, **5, 13**, **14**, **15**, **16**, and for most selective AChE inhibitor **8**, the stoichiometry of the biscarbamate-metal complexes was evaluated using the mole fraction method. The changes of absorbance were recorded at 245 nm and plotted against the metal mole fraction (x_M_, Figure 6). Formation of biscarbamate-metal complexes in a ratio 1:1 with all three biometals was determined for biscarbamates **1** and **15**, while biscarbamates **13** and **16** formed complexes with two biometals (compound **13** with Cu^2+^ and Fe^2+^, and compound **16** with Zn^2+^ and Cu^2+^). Biscarbamates **2**, **4** and **5** chelated one biometal in ratio 1:1 (complexes **2**−Fe^2+^, **4**−Cu^2+^ and **5**−Fe^2+^). The observed stoichiometry of complexes for biscarbamates **2**, **12** and **14** with two biometals (**2** with Zn^2+^ and Cu^2+^, **12** with Cu^2+^ and Fe^2+^, and **14** with Zn^2+^ and Fe^2+^) differed from 1:1, indicating that chelation probably involved more than one molecule of biscarbamate and one biometal ion, as is the case for complex **12**−Cu^2+^ in which the metal-biscarbamate ratio was 3:2 or complex **14**−Cu^2+^ with a ratio 7:3. A similar situation was determined for biscarbamate **8**, which formed complexes with Cu^2+^. For three biscarbmates, the stoichiometry of biscarbamate-metal complexes with two out of three biometals (**4** with Zn^2+^ and Fe^2+^, **5** with Zn^2+^ and Cu^2+^, and compound **8** with Zn^2+^ and Fe^2+^) could not be determined using mole fraction method, indicating that those biscarbamates had a lower capacity to form complex metals and formed complexes only in excess concentrations of metals. The same was the case with three biscarbamates in combination with one of the biometals (**12** with Zn^2+^, **13** with Zn^2+^, and **16** with Fe^2+^). As an example of stoichiometry determination, the stoichiometry of the most selective BChE inhibitor, compound **13**, is presented (Figure 6). For the complex of **13**-Cu^2+^, a sharp increase in absorbance followed by a sharp decline resulted in two straight lines for both absorption changes intersected at the metal mole fraction of 0.5 (Figure 6), pointing to the fact that Cu^2+^and compound **13** bonded stoichiometrically in a 1:1 ratio. The same trend was observed when the **13**-Fe^2+^ complex was analyzed. However, for the **13**−Zn^2+^ complex, although spectral changes and differential spectrum Figure 5B (green line) indicated that **13** could chelate Zn^2+^, the stoichiometry could not be determined.

### 2.6. The BBB Penetration Ability of Biscarbamates

The ability of biscarbamates to cross the BBB was estimated by comparing the calculated values of six physicochemical descriptors of compounds with the recommended values obtained for known CNS-active drugs [52,53,54,55]. CNS-active drugs generally have a molecular weight lower than 500 g mol^−1^, moderate hydrophobicity (logP < 5), less than five hydrogen bonds donors (HBD), less than ten hydrogen bond acceptors (HBA), and less than ten rotatable bonds (RB) and are less polar (polar surface area (PSA) < 90 Å^2^) than drugs that are not active in the CNS. The calculated values of molecular descriptors for the biscarbamates and recommended values [54,55,56] for CNS active drugs are shown in Table S2 in Supplementary Materials.

For the two compounds with the same amine substituent in the hydroxyaminoethyl chain, piperidine, i.e., compounds **2** (bisdimethyl carbamate) and **23** (bispiridinyl carbamate), in silico determined physicochemical descriptors were in the range of the upper recommended values, based on which it was estimated that they should be able to pass the BBB. For five biscarbamates **1**, **3**, **4**, **12**, and **13**, the PSA value showed a minimal deviation from the upper recommended value (Δ = 0.134 Å^2^), and it can be expected that these compounds could be able to pass the BBB since, according to the literature, some compounds with an even higher PSA can penetrate the BBB [52,56]. The physicochemical properties of the rest of the compounds showed a deviation of two or three physicochemical descriptors and are unlikely to be able to cross the BBB.

The values of the physicochemical properties for the tested biscarbamates were compared to those calculated for rivastigmine, carbamate currently in use for the treatment of AD and bambuterol. It seems that all of the tested carbamates are more likely to penetrate through the BBB than bambuterol, whose low BBB penetration can be accounted to its low logP value, lower than recommended for CNS active drugs [57]. All of the biscarbamates had higher values of molecular descriptors than rivastigmine.

### 2.7. Cytotoxicity

The cytotoxic effect of carbamates was evaluated on HepG2, HEK293 and SH-SY5Y in a 24 h exposure period. The concentration range was selected to correspond to the concentration range of carbamates used in in vitro kinetic experiments. The obtained results are given in Table 2.

Fourteen out of eighteen biscarbamates did not exhibit hepatotoxicity, nephrotoxicity or neurotoxicity in the concentration range in which they displayed BChE or AChE inhibition activity. Four biscarbamates (**10**, **14**, **17** and **18**) were toxic to all of the tested cell lines at the concentrations in which they showed inhibition activity.

## 3. General Discussion

This study has shown that biscarbamates with a meta disposition of carbamate groups on the benzene ring are a promising structural base for the design of novel AD drugs aimed to alleviate the symptoms of the disease. This is particularly true for agents aimed to be used in the middle and late stages of the disease as the majority of tested carbamates were more than 10 times more selective to BChE than AChE (two of them more than 1000 times more selective), a feature that is considered beneficial due to the fact that ACh is mainly hydrolyzed by AChE in the early stage and by BChE in the late stage. Beside selectivity, the tested biscarbamates are very fast BChE inhibitors whose inhibition potency is equally or up to 690 times more potent than rivastigmine’s, an ethylmethyl carbamate currently in use for treatment of AD and approved by the FDA. Generally, structure-activity analysis did not detect clear correlation between the length and/or the size of groups on carbamoyl nitrogen and inhibition potency of compounds. The size of aliphatic substituents did not affect the inhibition potency of the compounds, but the increase in inhibition potency can be expected when cyclic substituents were introduced. That observation is in line with that of Wu et al., who synthesized biscarbamates with aliphatic substituents on carbamoyl nitrogen [36].

The additional beneficial feature of the here presented compounds is their ability to chelate Fe^2^+, Cu^2+^ and Zn^2+^, biometals whose dishomeostasis is related to the pathophysiology of AD through the production of free radicals or formation of toxic metal-Aβ plaques. In this respect, this study has identified the tested compounds as potential multi-target-directed ligands in AD treatment able to inhibit cholinesterases and chelate at least one of the biometals. The ability to chelate biometals is especially interesting in terms of ferroptosis, an iron-dependent mechanism of regulated cell death associated with an increase in oxidative stress generated by free radicals formed via the Fenton reaction. Due to its correlation to the etiopathology of AD, ferroptosis is proposed as a promising a new target for the treatment of AD [58]. The non-toxicity of most of them towards neural, liver and kidney cells in the concentration range in which they displayed BChE or AChE inhibition activity speaks in favor of the possibility of using the investigated biscarbamates as drugs. For two compounds, it was estimated that they should be able to pass the BBB by passive transport, and for five more, this ability is expected to be slightly reduced.

Development of biscarbamates as AD drugs provides the possibility of developing long-acting drugs in two ways. First, a slow decarbamylation process assures a prolonged action per se, and second, the product of the first decarbamylation process is a monocarbamate that can also inhibit one or both cholinesterases, as was earlier determined for the monocarbamate of bambuterol [22,37].

Considering all of the beneficial features, this study has singled out compounds **2** and **16** as the most promising compounds for the treatment of AD. They strongly and preferentially inhibit BChE, are non-toxic, have the potential to cross the BBB and, compared to rivastigmine, possess the ability to chelate biometals.

## 4. Materials and Methods

### 4.1. Synthesis of Compounds

All chemicals, reagents and solvents for the synthesis of biscarbamates were purchased from commercial sources and were used without further purification. Biscarbamates were synthesized starting from 3, 5-dihroxyacethophenone using a slightly modified protocol as previously described in Wu [37]. For the synthesis, two methods were applied: Method 1 without hydrochloride formation and Method 2 with hydrochloride formation, described in detail in Supplementary Materials. The reactions were monitored using thin-layer chromatography on Silica Gel aluminum sheets and visualized with a UV lamp. NMR spectra were recorded on Bruker AV 600 MHz and 300 MHz spectrometers, operating at 150.92 or 75.47 MHz for ^13^C and 600.13 or 300.13 MHz for ^1^H nuclei. Chemical shifts are quoted in ppm and are referenced to SiMe_4_ as internal standard unless stated otherwise. Multiplets are abbreviated as follows: br—broad; s—singlet; d—doublet; t—triplet; q—quartet; m—multiplet. Mass spectra were recorded on a 4800 Plus MALDI-TOF/TOF mass spectrometer (Applied Biosystems Inc., Foster City, CA, USA) equipped with a 200 Hz, 355 nm Nd: YAG laser. Compounds were purified using column chromatography with silica gel as the stationary phase and dichloromethane/methanol mixtures as the eluent system.

The purity of the final compounds was determined using high-performance liquid chromatography (HPLC) with a Shimadzu 10A VP HPLC system; column: Nucleosil 100-5 C18; column dimensions: 250 mm × 4.6 mm; flow: 1 mL/min; injected volume: 20 μL; UV detection: 200–400 nm; gradient method with mobile phase A (H_2_O: MeOH: H_3_PO_4_ (85%) = 90: 10: 0.5) and mobile phase B (MeOH) with gradient washing: 50/100/100/50% B in time intervals of 0/20/25/27 min.

5-(2-cyclohexylamino)-1-hydroxyethyl)-1,3-phenylen bis(dimethylcarbamate) hydrochloride (**1**)

Compound **1** was synthesized with Method 2. Yield starting from 3,5 dihidroxyacetophenone: 21%. The purity of the compound, determined by HPLC, was 97%. MALDI TOF/TOF for C_20_H_31_N_3_O_5_ ([M+H]^+^): calculated 394.2342, found 394.2346. ^1^H NMR (CDCl_3_, 300 MHz), δH/ppm: 7.06 (d, *J* = 2.2 Hz, 2H); 6.88 (t, *J* = 2.2 Hz, 1H); 5.10 (dd, *J* = 9.7; 2.7 Hz, 1H); 4.64 (br s, 2H); 3.15 (dd, *J* = 12.3.; 3.1 Hz, 1H); 3.07 (s, 6H); 2.98 (s, 6H); 2.90−2.81 (m, 2H); 2.09−2.02 (m, 2H); 1.81−1.76 (m, 2H); 1.63 (d, *J* = 12.1 Hz; 1H,); 1.44−1.33 (m, 2H); 1.28−1.14 (m, 4H). ^13^C NMR (CDCl_3_, 75 MHz) δ/ppm 154.60; 151.90; 143.57; 116.14; 115.23; 69.12; 57.78; 52.50; 36.73; 36.47; 30.71; 30.51; 25.22; 24.64.

5-(1-hydroxy-2-(piperidin-1-yl)-ethyl)-1,3-phenylen bis(dimethylcarbamate) hydrochloride (**2**)

Compound **2** was synthesized with Method 2. Yield starting from 3,5-dihidroxyacetophenone: 35%. The purity of the compound, determined by HPLC, was 97%. MALDI TOF/TOF for C_19_H_29_N_3_O_5_ ([M+Na]^+^): calculated 402.2005, found 402.1991. ^1^H NMR (CDCl_3_, 300 MHz) δH/ppm: 7.07 (d, *J* = 2.2 Hz, 2H); 6.89 (t, *J* = 2.0 Hz, 1H); 5.41 (t, *J* = 6.4 Hz, 1H); 3.11-3.08 (m, 4H); 3.07 (s, 6H); 3.01-2.99 (m, 2H); 2.98 (s, 6H); 2.14-1.88 (m, 4H); 1.74−1.56 (m, 2H). ^13^C NMR (CDCl_3_, 75 MHz) δC/ppm: 154.43; 154.06; 152.20; 152.07; 142.08; 120.20; 116.15; 115.52; 71.18; 67.44; 65.09; 60.40; 36.81; 36.73; 36.53; 36.48; 22.89; 21.94.

5-2-(2-(adamantyl-1-amino-1-hydroxyethyl)-1,3-phenylen bis(dimethylcarbamate) hydrochloride (**3**)

Compound **3** was synthesized with Method 2. Yield starting from 3,5-dihidroxyacetophenone: 30%. The purity of the compound, determined by HPLC, was 99%. MALDI TOF/TOF for C_24_H_35_N_3_O_5_ ([M+H]^+^): calculated 446.2655, found 446.2669. ^1^H NMR (CDCl_3_, 600 MHz) δH/ppm: 6.98 (d, *J* = 2.2 Hz, 2H); 6.89 (t, *J* = 2.2 Hz, 1H); 4.56 (dd, *J* = 8.6, 3.7 Hz, 1H); 3.08 (s, 6H); 3.03 (s, 6H); 2.97 (dd, *J* = 12.1, 3.8 Hz, 1H); 2.61 (dd, *J* = 12.1, 8.6 Hz, 1H); 2.08−2.02 (m, 3H); 1.71−1.54 (m, 12H). ^13^C NMR (CDCl_3_, 151 MHz) δC/ppm: 154.54; 151.80; 145.29; 115.49; 114.43; 71.66; 50.34; 47.95; 43.05; 36.68; 36.64; 36.44; 29.55.

5-(2-(cyclohexylamino)-1-hydroxyethyl)-1,3-phenylen bis(ethyl(methyl)carbamate) hydrochloride (**4**)

Compound **4** was synthesized with Method 2. Yield starting from 3,5-dihidroxyacetophenone: 68%. The purity of the compound, determined by HPLC, was 97%. MALDI TOF/TOF for C_22_H_35_N_3_O_5_ ([M+H]^+^): calculated 422.2655, found 422.2646. ^1^H NMR (CDCl_3_,300 MHz) δH/ppm: 7.10 (d, *J* = 2.5 Hz, 2H); 6.92 (t, *J* = 2.3 Hz, 1H); 5.34 (d, *J* = 9.6 Hz, 1H); 3.47 (q, *J* = 7.1 Hz, 2H); 3.39 (q, *J* = 7.2 Hz, 2H); 3.26 (dd, *J* = 11.5, 2.6 Hz, 1H); 3.06−2.89 (m, 8H); 2.17 (t, *J* = 11.8 Hz, 2H); 1.85 (d, *J* = 12.5 Hz, 2H); 1.64−1.50 (m, 3H); 1.28−1.14 (m, 9H).^13^C NMR (CDCl_3_,300 MHz) δC/ppm: 154.22; 154.07; 151.97; 142.61; 116.18; 115.55; 68.28; 58.41; 52.14; 44.14; 34.25; 33.81; 29.25; 29.16; 24.81; 24.47; 13.19; 12.41.

5-(2-(*tert*-pentylamino)-1-hydroxyethyl)-1,3-phenylen bis(ethyl(methyl)carbamate) hydrochloride (**5**)

Compound **5** was synthesized with Method 2. Yield starting from 3,5-dihidroxyacetophenone: 15%. The purity of the compound, determined by HPLC, was 97%. MALDI TOF/TOF for C_21_H_35_N_3_O_5_ ([M+H]^+^): calculated 410.2655, found 410.2667. ^1^H NMR (CDCl_3_, 300 MHz) δH/ppm: 7.10 (d, *J* = 2.2 Hz, 2H); 6.96 (t, *J* = 2.2 Hz, 1H); 5.41 (d, *J* = 9.5 Hz, 1H); 3.46 (q, *J* = 7.1 Hz, 2H); 3.40 (q, *J* = 7.1 Hz, 2H); 3.25 (d, *J* = 11.9 Hz, 1H); 3.04 (s, 3H); 2.97 (s, 3H); 2.92 (dd, *J* = 12.1, 10.3 Hz, 1H); 1.78 (q, *J* = 7.5, 2H); 1.42 (s, 3H); 1.39 (s, 3H); 1.26−1.15 (m, 6H); 1.01 (t, *J* = 7.4 Hz, 3H). ^13^C NMR (CDCl_3_, 300MHz) δC/ppm: 154.14; 153.98; 151.98; 142.05; 115.96; 115.69; 67.92; 60.91; 49.48; 44.12; 34.25; 33.84; 31.03; 29.70; 23.19; 23.17; 13.19; 12.42; 796.

5-2-(2-(adamantyl-1-amino1-hidroxyethyl)-1,3-phenylen bis(ethyl(methyl) carbamate) hydrochloride (**6**)

Compound **6** was synthesized with Method 2. Yield starting from 3,5-dihidroxyacetophenone: 67%. The purity of the compound, determined by HPLC, was 98%. MALDI TOF/TOF for C_26_H_39_N_3_O_5_ ([M+H]^+^): calculated 474.2968, found 474.2980. ^1^H NMR (CDCl_3_, 300 MHz) δH/ppm: 7.12 (d, *J* = 1.8 Hz, 2H); 6.93 (s, 1H); 5.44 (d, *J* = 7.6 Hz, 1H); 3.50−3.32 (m, 4H); 3.28 (t, *J* = 11.2 Hz, 1H); 3.03 (s, 3H); 2.96 (s, 3H); 2.20−1.95 (m, 11H); 1.85 (s, 4H); 1.26−1.12 (m, 7H). ^13^C NMR (CDCl_3_, 75 MHz) δC/ppm: 154.27; 154.12; 151.92; 142.53; 116.28; 115.71; 67.95; 58.36; 53.25; 47.73; 44.12; 40.17; 38.55; 35.47; 34.25; 33.84; 29.01; 28.97; 13.21; 12.43.

5-(1-hydroxy-2-((2-phenylethyl)amino)ethyl)-1,3-phenylen bis(ethyl(methyl)carbamate) hydrochloride (**7**)

Compound **7** was synthesized with Method 2. Yield starting from 3,5-dihidroxyacetophenone: 10%. The purity of the compound, determined by HPLC, was 96%. MALDI TOF/TOF for C_24_H_33_N_3_O_5_ ([M+K]^+^): calculated 482.2057, found 482.2043. ^1^H NMR (CDCl_3_, 300 MHz) δH/ppm: 7.31-7.29 (m, 2H); 7.25-7.22 (m, 3H); 7.06 (d, *J* = 3.5 Hz, 2H); 6.88-6.82

(m, 1H); 5.16 (d, *J* = 6.3 Hz, 1H); 3.46 (q, *J* = 7.1 Hz, 2H); 3.39 (q, *J* = 7.1 Hz, 2H); 3.27-3.21 (m, 2H); 3.16-3.07 (m, 2H); 3.04 (s, 3H); 3.00−2.97 (m, 3H); 2.96 (s, 2H); 1.27−1.14 (m, 6H).^13^C NMR (CDCl_3_, 75 MHz) δC/ppm: 154.35; 154.20; 151.91; 136.64; 128.85; 128.77; 128.54; 126.95; 116.36; 115.60; 68.20; 54.65; 49.76; 44.17; 34.29; 33.83; 32.40; 13.18; 12.41.

5-(1-hydroxy-2-(*tert*-pentylamino)-ethyl)-1,3-phenylen bis (diethylcarbamate) hydrochloride (**8**)

Compound **8** was synthesized with Method 2. Yield starting from 3,5-dihidroxyacetophenone: 25%. The purity of the compound, determined by HPLC, was 97%. MALDI TOF/TOF for C_23_H_39_N_3_O_5_ ([M+H]^+^): calculated 438.2968, found 438.2971. ^1^H NMR (CDCl_3_, 300 MHz) δH/ppm: 7.11 (d, *J* = 2.1 Hz, 2H); 6.87 (t, *J* = 2.1 Hz, 1H); 5.36 (d, *J* = 10.4 Hz, 1H); 3.45−3.29 (m, 8H); 3.21 (d, *J* = 11.0 Hz, 1H); 2.92 (t, *J* = 11.2 Hz, 1H); 1.75−1.64 (m, 2H); 1.38 (d, *J* = 7.0 Hz, 6H); 1.28−1.12 (m, 12H); 0.96 (t, *J* = 7.4 Hz, 3H).^13^C NMR (CDCl_3_, 300MHz) δC/ppm: 153.86; 151.92; 142.66; 116.03; 115.55; 68.51; 59.61; 49.39; 42.26; 41.91; 31.50; 23.66; 14.20; 13.33; 8.06.

5-(2-(adamantyl-1-amino)-1-hydroxyethyl)-1,3-phenylen bis (diethylcarbamate) hydrochloride (**9**)

Compound **9** was synthesized with Method 2. Yield starting from 3,5-dihidroxyacetophenone: 70%. The purity of the compound, determined by HPLC, was 95%. MALDI TOF/TOF for C_28_H_43_N_3_O_5_ ([M+H]^+^): calculated 502.3281, found 502.3279. ^1^H NMR (CDCl_3_, 300 MHz) δH/ppm: 7.14 (d, *J* = 2.1, 2H); 6.89 (t, *J* = 2.1 Hz, 1H); 5.44 (d, *J* = 9.1 Hz, 1H); 3.47−3.32 (m, 8H); 3.27 (d, *J* = 3.3 Hz, 1H); 3.06 (t, *J* = 11.1 Hz, 1H); 2.20−1.96 (m, 12H); 1.70 (br s, 5H); 1.28−1.14 (m, 12H). ^13^C NMR (CDCl_3_, 75 MHz) δC/ppm: 154.18; 151.83; 143.08; 116.72; 115.92; 67.98; 58.32; 53.27; 47.31; 42.42; 42.01; 40.24; 38.70; 35.52; 29.03; 28.98; 14.17; 13.34.

5-(1-hydroxy-2-((1-phenylethyl) amino) ethyl)-1,3-phenylen bis (diethylcarbamate) hydrochloride (**10**)

Compound **10** was synthesized with Method 2. Yield starting from 3,5-dihidroxyacetophenone: 49%. The purity of the compound, determined by HPLC, was 97%. MALDI TOF/TOF for C_26_H_37_N_3_O_5_ ([M+H]^+^): calculated 472.2811, found 472.2820. ^1^H NMR (CDCl_3_, 300 MHz) δH/ppm: 7.36−7.20 (m, 5H); 6.94−6.90 (m, 2H); 6.89−6.87 (m, 1H); 4.63 (ddd, *J* = 43.5, 8.4, 3.7 Hz, 1H); 3.86−3.69 (m, 1H); 3.45−3.21 (m, 8H); 2.85−2.73 (m, 1H); 2.69-2.53 (m, 1H); 1.40 (dd, *J* = 6.8, 3.2 Hz, 3H); 1.25−1.14 (m, 12H). ^13^C NMR (CDCl_3_, 75 MHz) δC/ppm: 153.82; 151.79; 145.16; 144.89; 144.72; 144.60; 128.58; 127.10; 127.07; 126.52; 126.48; 115.67; 114.69; 71.54; 71.29; 58.46; 57.67; 54.82; 54.40; 42.22; 41.88; 24.24; 24.14; 14.21; 13.36.

5-(1-hydroxy-2-phenylethyl)amino)ethyl)-1,3-phenylen bis (diethylcarbamate) hydrochloride (**11**)

Compound **11** was synthesized with Method 2. Yield starting from 3,5-dihidroxyacetophenone: 47%. The purity of the compound, determined by HPLC, was 98%. MALDI TOF/TOF for C_26_H_37_N_3_O_5_ ([M+H]^+^): calculated 472.2811, found 472.2815. ^1^H NMR (CDCl_3_, 300 MHz) δH/ppm: 7.34−7.28 (m, 2H); 7.24−7.15 (m, 2H); 7.00 (d, *J* = 2.2 Hz, 2H); 6.89 (t, *J* = 2.2 Hz, 1H); 4.83 (dd, *J* = 9.1; 3.4 Hz, 1H); 3.48-3.26 (m, 8H); 3.07−2.73 (m, 8H); 1.31-1.08 (m, 12H). ^13^C NMR (CDCl_3_, 75 MHz) δC/ppm: 161.86; 153.89; 151.85; 144.19; 138.77; 128.75; 128.60; 126.46; 11.590; 114.96; 70.11; 56.03; 50.29; 42.27; 41.91; 35.14; 14.21; 13.35.

5-(2-cyclohexylamino)-1-hydroxyethyl)-1,3-phenylen bis (pyrolidin-1-carboxyilate)hydrochloride (**12**)

Compound **12** was synthesized with Method 2. Yield starting from 3,5-dihidroxyacetophenone: 35%. The purity of the compound, determined by HPLC, was 95%. MALDI TOF/TOF for C_24_H_35_N_3_O_5_ ([M+H]^+^): calculated 446.2655, found 446.2661. ^1^H NMR (CDCl_3_, 300 MHz) δH/ppm: 7.08 (d, *J* = 2.1 Hz, 2H); 6.95 (t, *J* = 2.1 Hz, 1H) 5.23 (d, *J* = 9.0 Hz, 1H); 3.55−3.50 (m, 4H); 3.45−3.40 (m, 4H); 3.21 (dd, *J* = 12.3; 2.8 Hz, 1H); 3.00−2.88 (m, 2H); 2.20−2.10 (m, 2H); 1.98−1.88 (m, 8H); 1.85−1.78 (m, 2H); 1.55−1.42 (m, 2H); 1.28−1.15 (m, 4H).^13^C NMR (CDCl_3_, 75 MHz) δC/ppm: 152.78; 151.86; 142.82; 115.98; 115.28; 68.73; 58.09; 52.61; 46.48; 46.37; 29.76; 29.69; 25.78; 24.96; 24.54.

5-(1-hydroxy-2-(*tert*-pentylamino)-ethyl)-1,3-phenylen bis (pyrolidin-1-carboxyilate) hydrochloride (**13**)

Compound **13** was synthesized with Method 2. Yield starting from 3,5-dihidroxyacetophenone: 55%. The purity of the compound, determined by HPLC, was 98%. MALDI TOF/TOF for C_23_H_35_N_3_O_5_ ([M+H]^+^): calculated 434.2655, found 434.2667. ^1^H NMR (CDCl_3_, 300 MHz) δH/ppm: 7.11 (s, 2H); 6.96 (s, 1H); 5.41 (d, *J* = 9.0 Hz, 1H); 3.62-3.34 (m, 8H); 3.28 (d, *J* = 11.9 Hz, 1H); 2.97 (t, *J* = 11.2 Hz, 1H); 2.04−1.84 (m, 8H); 1.84−1.66 (m, 2H); 1.53−1.29 (m, 6H); 1.03 (t, *J* = 7.4 Hz, 3H). ^13^C NMR (CDCl_3_, 75 MHz) δC/ppm: 152.76; 151.87; 142.12; 115.98; 115.58; 67.91; 60.92; 49.51; 46.48; 46.39; 31.07; 25.77; 24.95; 23.13; 7.99.

5-(2-(adamantyl-1-amino-1-hidroxyethyl)-1,3-phenylen bis (pyrolidin-1-carboxyilate) hydrochloride (**14**)

Compound **14** was synthesized with Method 2. Yield starting from 3,5-dihidroxyacetophenone: 58%. The purity of the compound, determined by HPLC, was 98%. MALDI TOF/TOF for C_28_H_39_N_3_O_5_ ([M+H]^+^): calculated 498.2968, found 498.2991. ^1^H NMR (CDCl_3_, 300MHz) δH/ppm: 7.12 (d, *J* = 2.1 Hz, 2H); 6.96 (t, *J* = 2.3 Hz, 1H); 5.37 (d, *J* = 9.3 Hz, 1H); 3.55 (t, *J* = 6.5 Hz, 4H); 3.47 (t, *J* = 6.3 Hz, 4H); 3.25 (d, *J* = 11.3 Hz, 1H); 3.02 (t, *J* = 11.3 Hz, 1H); 2.18−1.87 (m, 19H); 1.73−1.59 (m, 6H).^13^C NMR (CDCl_3_, 300MHz) δC/ppm: 152.78; 151.87; 142.33; 116.06; 115.60; 68.08; 58.25; 53.43; 47.76; 46.48; 46.37; 38.59; 35.48; 29.08; 25.77; 24.97.

5-(2-cyclolohexylamino)-1-hydroxyethyl)-1,3-phenylen bis(piperidine-1)-carboxyilate hydrochloride (**15**)

Compound **15** was synthesized with Method 2. Yield starting from 3,5-dihidroxyacetophenone: 28%. The purity of the compound, determined by HPLC, was 95%. MALDI TOF/TOF for C_26_H_39_N_3_O_5_ ([M+H]^+^): calculated 474.2968, found 474.2970. ^1^H NMR (CDCl_3_, 300 MHz) δC/ppm: 7.06 (d, *J* = 2.0 Hz, 2H); 6.91 (t, *J* = 2.2 Hz, 1H); 5.34 (d, *J* = 9.4 Hz, 1H); 3.60−3.44 (m, 8H); 3.27 (d, *J* = 11.3 Hz, 1H); 3.03−2.97 (m, 2H); 2.19−2.12 (m, 2H); 1.85 (d, *J* = 11.2 Hz, 2H); 1.67-1.49 (m, 14H) 1.28-1.20 (m, 4H). ^13^C NMR (CDCl_3_, 75 MHz) δC/ppm: 153.28; 152.04; 142.34; 116.03; 115.59; 68.37; 58.46; 52.54; 45.52; 45.12; 29.69; 29.17; 25.86; 25.50; 24.79; 24.44; 24.28; 24.25.

5-(1-hidroxy-2-(piperidin-1-il)ethyl)-1,3-phenylen bis(piperidine-1)-carboxyilate hydrochloride (**16**)

Compound **16** was synthesized with Method 2. Yield starting from 3,5-dihidroxyacetophenone: 50%. The purity of the compound, determined by HPLC, was 95%. MALDI TOF/TOF for C_25_H_37_N_3_O_5_ ([M+H]^+^): calculated 460.2811, found 460.2817. ^1^H NMR (CDCl_3_, 600 MHz) δH/ppm: 7.05 (d, *J* = 2.4 Hz, 2H); 6.90 (t, *J* = 2.4 Hz, 1H); 5.42 (dd, *J* = 8.2; 4.5 Hz, 1H); 3.62-3.40 (m, 12H); 3.14-3.11 (m, 2H); 1.71−1.53 (m, 18H). ^13^C NMR (CDCl_3_, 150 MHz) δC/ppm: 153.20; 152.09; 142.01; 116.10; 115.46; 67.73; 64.90; 54.96; 45.58; 45.13; 25.84; 25.49; 24.22; 22.72; 21.79.

5-(2-(1-adamantylamino)-1-hydroxyethyl)-1,3-phenylen bis(piperidine-1)-carboxylate hydrochloride (**17**)

Compound **17** was synthesized with Method 2. Yield starting from 3,5-dihidroxyacetophenone: 20%. The purity of the compound, determined by HPLC, was 96%. MALDI TOF/TOF for C_30_H_43_N_3_O_5_ ([M+H]^+^): calculated 526.3281, found 526.3300. ^1^H NMR (CDCl_3_, 300 MHz) δH/ppm: 7.12 (d, *J* = 2.1 Hz, 2H); 6.96 (t, *J* = 2.3 Hz, 1H); 5.40 (d, *J* = 9.7 Hz, 1H) 3.62−3.41 (m, 8H); 3.29−3.17 (m, 1H); 2.98 (t, *J* = 11.3 Hz, 1H); 2.20−1.94 (m, 10H); 1.74−1.54 (m, 18H).^13^C NMR (CDCl_3_, 75 MHz) δC/ppm: 153.21; 152.09; 142.02; 116.12; 115.64; 67.33; 64.09; 54.95; 45.55; 45.14; 25.85 25.50; 24.23; 22.72; 21.79.

5-(2-(cyclohexyilamino)-1-hidroxyethyl)-1,3-phenyen bis(methyl(phenyl)carbamate) hydrochloride) (**18)**

Compound **18** was synthesized with Method 2. Yield starting from 3,5-dihidroxyacetophenone: 38%. The purity of the compound, determined by HPLC, was 97%. MALDI TOF/TOF for C_30_H_35_N_3_O_5_ ([M+H]^+^): calculated 518.2655, found 518.2632. ^1^H NMR (CDCl_3_, 600 MHz) δH/ppm: 7.37 (t, *J* = 7.8 Hz, 4H), 7.31 (d, *J* = 8.0 Hz, 4H), 7.26−7.20 (m, 3H), 7.11−7.00 (m, 2H), 5.30 (d, *J* = 5.6 Hz, 1H), 3.37 (br s, 6H), 3.20 (d, *J* = 12.3 Hz, 1H), 3.00−2.90 (m, 2H), 2.11 (t, *J* = 14.5 Hz, 2H), 1.83−1.75 (m, 2H), 1.65−1.44 (m, 4H), 1.30−1.10 (m, 4H). ^13^C NMR (CDCl_3_, 151 MHz) δC/ppm: 153.54; 151.74; 142.50; 129.10; 126.82; 126.10; 116.20; 115.28; 68.28; 58.43; 52.43; 38.32; 29.17; 24.74; 24.40.

### 4.2. Cholineseterase Inhibition

#### 4.2.1. Enzyme Activity Measurements

Enzyme activities were determined using the Ellman spectrophotometric method [59]. Enzyme substrates acetylthiocholine iodide (ATCh) and propionylthiocholine iodide (PTCh) were purchased from Sigma-Aldrich, Steinheim, Germany, and thiole reagent 5.5′-dithiobis-2-nitrobenzoic acid (DTNB) from Sigma-Aldrich, St. Louis, MO, USA; ATCh and PTCh were dissolved in water and DTNB in 0.1 M sodium phosphate buffer (pH 7.4). Biscarbamates were dissolved in water, and all further dilutions were made in water.

Sources of AChE and BChE were native human erythrocytes and native human plasma, respectively.

Final concentrations of carbamates were in the range of 0.01–200 µM, while substrates were 1.0 mM and 4.0 mM for ATCh and PTCh, respectively. The final dilution of AChE and BChE was 500 and 300 times, respectively.

#### 4.2.2. Inhibition by Biscarbamates

In inhibition experiments, enzyme samples were incubated for up to 30 min with biscarbamates in the absence of substrate using 3–5 inhibitor concentrations ranging from 0.5–200 μM. An inhibitor was added to the reaction mixture containing DTNB, buffer and enzyme. After designated period of time, the inhibition reaction was stopped by the addition of substrate (4.0 mM PTCh for BChE, or 1.0 mM ATCh for AChE). The extent of inhibition was determined by measuring the residual activity of the enzyme. To measure the enzyme activity at zero time of inhibition, the enzyme was added to a reaction mixture containing DTNB, buffer, inhibitor and substrate immediately before the start of the measurement. With the inhibited probes, the activities of the control probes, which did not contain an inhibitor, were also measured. For each carbamate and enzyme, at least three independent experiments were performed.

Enzyme inhibition proceeds according to the following scheme:


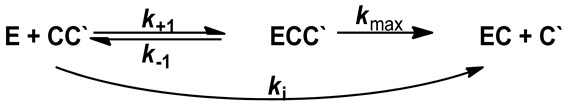


where E, CC′, ECC′, EC and C′ stand for free enzyme, inhibitor, Michaelis type complex between enzyme and inhibitor, carbamylated enzyme and monocarbamate, respectively. *k*_+1_, *k*_−1_, and *k*_max_ are rate constants of the respective reactions, while *k*_i_ is the overall second-order inhibition rate constant.

The first-order rate constants (*k*_obs_) were calculated by linear regression analysis at any given inhibitor concentration [CC′]:

(1) $$ln\frac{v_{0}}{v_{i}}=k_{obs}\cdot t$$

where *v*_0_ and *v*_i_ stand for the enzyme activity in the absence and in the presence of inhibitor at time *t*.

When the *k*_obs_ was a linear function of [CC′], the slope represented the second-order inhibition rate constant (*k*_i_)

When dependence of *k*_obs_ vs. [CC′] was not linear, indicating the presence of reversible enzyme–inhibitor complex, the maximum first-order inhibition rate constant (*k*_max_) and the dissociation constants of enzyme–inhibitor complex (*K*_i_) were determined from:

(2) $$\;k_{obs}=\frac{k_{max}\cdot \left[{\mathrm{CC}}^{\prime }\right]}{K_{i}+\left[{\mathrm{CC}}^{\prime }\right]}$$

Then the *k*_i_ constant was determined as the ratio:

(3) $$k_{i}=\frac{k_{max}}{K_{i}}$$

All kinetic parameters were calculated using the statistical package GraphPadPrism (Graph Pad Inc. San Diego, CA, USA).

### 4.3. Spontaneous Decarbamylation

The rate of spontaneous recovery of activity of cholinesterase inhibited by biscarbamates, i.e., the rate of spontaneous decarbamylation, was determined by monitoring the time course of return of cholinesterase activity.

The spontaneous decarbamylation proceeds according to scheme:


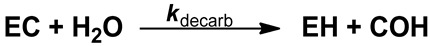


where the carbamylated enzyme reacts with water to form the free enzyme (EH) and corresponding alcohol (COH).

Before spontaneous decarbamylation measurements, BChE or AChE were incubated with 100 µM biscarbamates (incubation mixture) for 60 min to obtain the 90–100% inhibition of enzyme activity [48,49]. To avoid the possible reinhibition of the free enzyme released during the decarbamylation process, excess of biscarbamate was removed by filtering the reaction mixture through a filtration column (Strata^®^ C18-E, Phenomenex, Torrance, CA, USA). The time immediately after filtration was denoted as “zero” time of the onset of spontaneous decarbamylation. After a designated period of time (up to 8 h), aliquots of incubation mixture were added to a reaction mixture (buffer and 0.3 mM DTNB), and measurement of activity was started by the addition of substrate (ATCh for AChE and PTCh for BChE). An identical incubation mixture was prepared with enzyme and buffer instead of inhibitor to measure the control values of enzyme activity.

The decarbamylation rate constant (*k*_decarb_) was calculated according to the equation:

(4) $$ln\left[1-\frac{v_{t}}{v_{0}}\right]=-k_{decarb}$$

where *v*_0_ denotes the activity of the enzyme in the absence of inhibitor and *v*_t_ the activity of the enzyme incubated by the inhibitor in time *t*. The -*k*_decarb_ is represented by the slope of the line [60,61,62,63].

All kinetic parameters were calculated using the statistical package GraphPadPrism (Graph Pad Inc. San Diego, CA, USA).

### 4.4. Docking Studies

For docking ligands into the enzyme receptors, the Flexible Docking protocol was used [64]. Ligands to be docked in the enzyme structures were created with ChemBio3D Ultra 13.0 (PerkinElmer, Inc., Waltham, MA, USA) and minimized using the CHARMm force field and Smart Minimizer minimization method of Minimize Ligands protocol implemented in Biovia Discovery Studio Client v18.1. (Dassault Systèmes, Vélizy-Villacoublay, France). Before starting molecular docking protocol, Prepare Ligands protocol was used to prepare ligands with regards to possible different protonation states, isomers and tautomers at pH 7.4.

The enzyme structures were prepared starting from the crystal structures of free BChE (PDB ID: 1P0I) [65]. The binding site within BChE was defined by the sphere surrounding the residues that outline the active sites gorge including those that were selected as flexible: Asn68, Asp70, Trp82, Gln119, Trp128, Glu197, Ser198, Trp231, Pro285, Leu286, Ser287, Glu325, Phe329, Phe398 and His438. The representative pose of each of the docked ligands was selected based on the highest Consensus score calculated from the scoring functions used to estimate binding affinity, as implemented in the Biovia Discovery Studio Client v18.1. Score Ligand Poses protocol. Among those with highest Consensus score, the ones closest to fulfilling prerequisites for successful carbamylation reaction in terms of O_Ser198_–C=O distance and alignment were finally chosen as representative poses. The parameters used in in silico protocols were configured as reported in a previously published work [45].

### 4.5. Metal Chelation Studies

The ability of carbamates to chelate biometals was tested using metal salts: zinc (II) chloride (ZnCl_2_), copper chloride dihydrate (CuCl_2_·2H_2_O) and iron dichloride tetrahydrate (FeCl_2_·4H_2_O). All metal salts and biscarbamates were dissolved in methanol. Briefly, a fixed amount of biscarbamate (30 µM) was mixed with a fixed amount of metal salt (60 µM). The absorbance spectrum (200 to 600 nm) of such a mixture was measured in the first minute and 30 and 60 min after mixing. The spectra of metal salt and of biscarbamate were also measured [50,66].

Interaction between biscarbamates and metal was indicated by the changes of spectra of biscarbamates compared to that of biscarbamate-metal mixture and confirmed by the differential UV–VIS spectra. Differential UV–VIS spectra were obtained by numerical subtraction of the spectra of the metal and the biscarbamate from the spectra of the mixture of the metal and biscarbamate. By visual inspection of the differential spectra, 245 nm was denoted as the wavelength of maximum absorption corresponding to the formation of the biscarbamate-metal complexes.

The stoichiometry of the biscarbamate-metal complexes was determined using the mole fraction method [50,67]. The changes of absorbance (ΔA) were recorded at 245 nm and plotted against the metal mole fraction (x_M_). The intersection point in the plot corresponds to the mole fraction of metal in the biscarbamate-metal complex [50,51,66,67].

The study of metal chelation was performed in 1 cm quartz cuvette (final volume 1 mL) at 25 °C using UV–VIS spectrophotometer (Cary 300 spectrophotometer Varian, Inc., Melbourne, Australia). All presentations of the spectrum and stoichiometry of binding were carried out in GraphPad Prism, GraphPad Software.

### 4.6. In Silico Prediction of Blood–Brain Barrier (BBB) Penetration

The ability of synthesized biscarbamates to penetrate the BBB was estimated by calculating molecular descriptors important for passive transport [53,54] (optimal values of lipophilicity (log P), molecular weight (MW), a polar surface area (PSA), optimal numbers of H-bond donors (HBD) and H-bond acceptors (HBA)) and molecular flexibility characterized by the number of rotatable bonds (RB) using the Chemicalize protocol [68].The obtained results were compared to the upper and lower recommended values obtained for known CNS-active drugs [52,53,54,55,56,57].

### 4.7. Cytotoxicity of Biscarbamates

All cell growth and cell culture supplements (EMEM and DMEM F12 medium, fetal bovine serum (FBS), penicillin/streptomycin (PenStrep), glutamine and non-essential amino acids (NEAA)) were purchased from Sigma-Aldrich, Steinheim, Germany. Cell cultivation was performed according to a previously described protocol [69].

The human Caucasian hepatocyte carcinoma HepG2 (ECACC 85011430), human embryo kidney HEK293 (ECACC 85120602) and human neuroblastoma SH-SY5Y (ECACC 94030304) cell lines were obtained from certified cell-bank European Collection of Authenticated Cell Cultures (ECACC) through Sigma-Aldrich (Steinheim, Germany). All cell lines were grown and maintained according to standard protocol [69,70,71].

The cytotoxic profiles of tested carbamates were determined by measuring the succinate dehydrogenase mitochondrial activity of cells exposed to them [69]. The commercially available MTS detection reagent assay was used (CellTiter 96^®^ AQueous One Solution Cell Proliferation Assay, Promega, Madison, WI, USA). The procedure followed slightly modified manufacturer protocol described previously [72]. Data were presented as percentage of the inhibited cells to control untreated cells, i.e., percentage of cytotoxicity. Staurosporine was used as positive control.

## Figures and Tables

**Table 1:** Inhibition of acetylcholinesterase (AChE) and butyrylcholinesterase (BChE) by biscarbamates.

Compound	BChE			AChE			*k*_i(BChE)_ */k*_i(AChE)_
	*k*_i_∙10^6^ (M^−1^ min^−1^)	*K*_i_ (µM)	*k_max_* (min^−1^)	*k*_i_∙10^6^ (M^−1^ min^−1^)	*K*_i_ (µM)	*k_max_* (min^−1^)	
**1**	1.54 ± 0.40	0.116 ± 0.027	0.180 ± 0.021	0.0261 ± 0.0102	14.9 ± 4.8	0.389 ± 0.086	59
**2**	1.74 ± 0.48	0.0754 ± 0.0188	0.131 ± 0.015	0.0312 ± 0.0032	5.16 ± 1.11	0.161 ± 0.014	56
**3**	0.0192 ± 0.0021	27.0 ± 6.5	0.582 ± 0.091	0.00330 ± 0.0013	80.5 ± 22.7	0.269 ± 0.044	6
**4**	1.47 ± 0.193	0.485 ± 0.049	0.739 ± 0.057	1.73 ± 0.44	0.383 ± 0.081	0.661 ± 0.090	0.88
**5**	2.28 ± 0.42	0.0485 ± 0.0083	0.110 ± 0.007	0.0402 ± 0.0063	4.57 ± 0.66	0.184 ± 0.013	56
**6**	3.68 ± 1.23	0.115 ± 0.038	0.423 ± 0.293	2.00 ± 0.70	-	-	1.8
**7**	0.205 ± 0.052	1.42 ± 0.30	0.292 ± 0.034	0.0310 ± 0.0132	3.29 ± 0.52	0.102 ± 0.007	6.6
**8**	1.64 ± 0.37	-	-	4.56 ± 1.18	0.0439 ± 0.0191	0.200 ± 0.141	0.36
**9**	2.18 ± 0.52	0.236 ± 0.045	0.514 ± 0.061	2.02 ± 0.65	0.181 ± 0.053	0.364 ± 0.161	1
**10**	0.0145 ± 0.0049	-	-	0.00693 ± 0.00210	-	-	2
**11**	0.0144 ± 0.0053	-	-	0.00679 ± 0.000217	-	-	2
**12**	1.05 ± 0.45	0.212 ± 0.078	0.224 ± 0.050	0.0193 ± 0.0096	41.4 ± 35.6	0.801 ± 0.576	55
**13**	4.51 ± 152	0.127 ± 0.025	0.571 ± 0.057	0.00360 ± 0.000303	-	-	1288
**14**	1.80 ± 0.37	0.378 ± 0.049	0.679 ± 0.062	0.00841 ± 0.00053	-	-	209
**15**	2.62 ± 0.97	0.155 ± 0.052	0.399 ± 0.073	0.0136 ± 0.0050	-	-	184
**16**	38.0 ± 6.7	0.0119 ± 0.0030	0.453 ± 0.065	0.0382 ± 0.0131	6.93 ± 2.04	0.264 ± 0.040	1087
**17**	1.89 ± 0.59	0.0910 ± 0.0229	0.171 ± 0.019	0.165 ± 0.074	2.23 ± 0.95	0.369 ± 0.116	12
**18**	1.63 ± 0.57	0.0622 ± 0.0129	0.101 ± 0.008	0.0182 ± 0.0063	15.8 ± 3.6	0.288 ± 0.044	91
Bambuterol	4.4 ± 0.2 *	-	-	0.000220 ± 0.000070 **	1900 ± 590	0.42 ± 0.007	19,600
Rivastigmine	0.0551 ± 0.0022			0.00222 ± 0.00046			25

* Reference [41]. ** Reference [34].

**Table 2:** The cytotoxicity of the tested compounds evaluated using MTS assay after 24 h cell exposure expressed as IC_50_ values ± SE (µM).

Compound	HEK293	HepG2	SH-SY5Y
**1**	≥800	≥800	≥800
**2**	≥800	≥800	≥800
**3**	240 ± 26	324 ± 30	197 ± 38
**4**	347 ± 8	398 ± 22	525 ± 32
**5**	≥800	≥800	≥800
**6**	112 ± 6	47.9 ± 1.3	89.1 ± 3.0
**7**	42.7 ± 9.2	83.2 ± 4.8	214 ± 15
**8**	234 ± 17	252 ± 10	240 ± 29
**9**	20.9 ± 2.6	14.8 ± 2.1	19.1 ± 1.1
**10**	11.5 ± 2.9	76.7 ± 13.0	17.0 ± 4.2
**11**	36.3 ± 1.2	33.7 ± 4.3	38.9 ± 3.6
**12**	251 ± 25	248 ± 5.7	178 ± 26
**13**	281 ± 9	457 ± 53	≥800
**14**	32.4 ± 6.5	41.7 ± 5.4	46.8 ± 4.3
**15**	38.0 ± 2.1	27.5 ± 1.7	30.2 ± 3.2
**16**	288 ± 32	295 ± 47	126 ± 25
**17**	6.76 ± 0.31	7.59 ± 0.26	8.19 ± 0.72
**18**	8.32 ± 0.95	15.3 ± 0.3	7.41 ± 0.09
Bambuterol	≥800	≥800	≥800

## Data Availability

Data is contained within the article and supplementary material.

## Supplementary Materials

The following supporting information can be downloaded at: https://www.mdpi.com/article/10.3390/ph15101220/s1, Synthesis scheme, Detailed description of synthesis method I and II, Figure S1: Structures of biscarbamates, Table S1: Decarbamylation rate constants, Table S2: Physiochemical properties of biscarbamates, 1H and 13C spectra of the compounds, HRMS spectra of the compounds, Tables S3–S20: The predicted non-bonding interactions between biscarbamtes and hBChE.
