# Supplementary material for: Design, Synthesis and Biological Evaluation of Biscarbamates as Potential Selective Butyrylcholinesterase Inhibitors for the Treatment of Alzheimer’s Disease
Source: Pharmaceuticals (Basel). 2022 Sep 30;15(10):1220. doi: 10.3390/ph15101220 (PMC9609992; doi:10.3390/ph15101220)
Supplement: Supplementary file 1 [file pharmaceuticals-15-01220-s001.zip › pharmaceuticals-1923858-supplementary.pdf]

# Supplementary Material

## Design, synthesis and biological evaluation of biscarbamates as potential selective butyrylcholinesetrase inhibitors for the treatment of Alzheimer`s disease

A. Matošević[1], A. Knežević[2], A. Zandona[1], N. Maraković[1], Z. Kovarik[1], A. Bosak[1*]

[1] *Institute for Medical Research and Occupational Health, Biochemistry and Organic Analytical Chemistry Unit, Zagreb, Croatia*

[2] *Ruđer Bošković Institute, Division of Organic Chemistry and Biochemistry, Zagreb, Croatia*

*Corresponding author: Anita Bosak, e-mail: abosak@imi.hr

Content:

Synthesis scheme

Detailed description of synthesis method I and II

Figure S1: Structures of biscarbamates

Table S1: Decarbamylation rate constants

Table S2: Physiochemical properties of biscarbamates

$^{1}H$ and $^{13}C$ spectra of the compounds

HRMS spectra of the compounds

Tables S3–S20: The predicted non-bonding interactions between biscarbamtes and hBChE

Biscarbamates were synthesized starting from 3,5-dihroxyacethophenone using slightly modified protocol as previously described in the Wu et all, 2017. For the synthesis two methods were applied: Method 1 without hydrochloride formation and Method 2 which includs hydrochloride formation.

Method 1: A mixture of 3,5-dihydroxyacetophenone (2 g, 16 mmol), triethylamine (4.5 mL) and 4-dimethylamino pyridine (0.25 g, 2 mmol) with R-carbamoyl chloride (3.8 g, 32 mmol) in 1,2- dichloroethane (40 mL) were stirred at 60 ˚C in an argon atmosphere for 24 h. The reaction mixture was cooled down to room temperature, dichloromethane (50 mL) was added, and the layers separated. The organic phase was washed twice with water and with 1M hydrochloric acid (10 mL), dried over $Na_2SO_4$ and filtered. The filtrate was concentrated to give the product A, which was used in the next step without further purification.

$CuBr_2$ (4.6 g; 20 mmol) was mixed with A (10 mmol) in an argon atmosphere in ethyl acetate (40 mL) and chloroform (40 mL) and stirred at 65 ˚C for 4.5 h. After cooling down, the mixture was filtered and washed with ethyl-acetate. Organic phase was washed twice with water, dried over $Na_2SO_4$ and filtered. The filtrate was evaporated and the crude product purified over a flash silica gel column as the stationary phase and dichloromethane /methanol mixtures as eluent system and concentrated to give intermediate B.

The compound B (1.25 mmol) was dissolved in methanol at room temperature and $NaBH_4$ (0.12 g, 3.12 mmol) was added to the solution. Dichloromethane (10 mL) was added to the suspension and the mixture was stirred for 2.5 hours at room temperature. The mixture was concentrated, redissolved in dichloromethane, and washed with the saturated solution of ammonium chloride. The organic phase was dried over $Na_2SO_4$, filtered and concentrated to produce oil C. The oil C was used in the next step of synthesis without further purification.

The oil C (1 mmol) was mixed with amine (2 mmol) in isopropanol. The mixture was stirred for 24 hours at 60-80˚C. After cooling down to room temperature, the resulting solution was evaporated to dryness. The oil product D was purified over a flash silica gel column chromatography (DCM:MeOH= 90:1 to 9:1) and concentrated to give a pure product.

Method 2: The oil D was dissolved in isopropanol and the pH of the solution was adjusted to 1 using hydrochloric acid in isopropanol. The mixture was concentrated

until it precipitated to produce final product E. The product E was purified over a flash silica gel column chromatography (DCM:MeOH= 90:1 to 9:1) and concentrated to give a pure product.

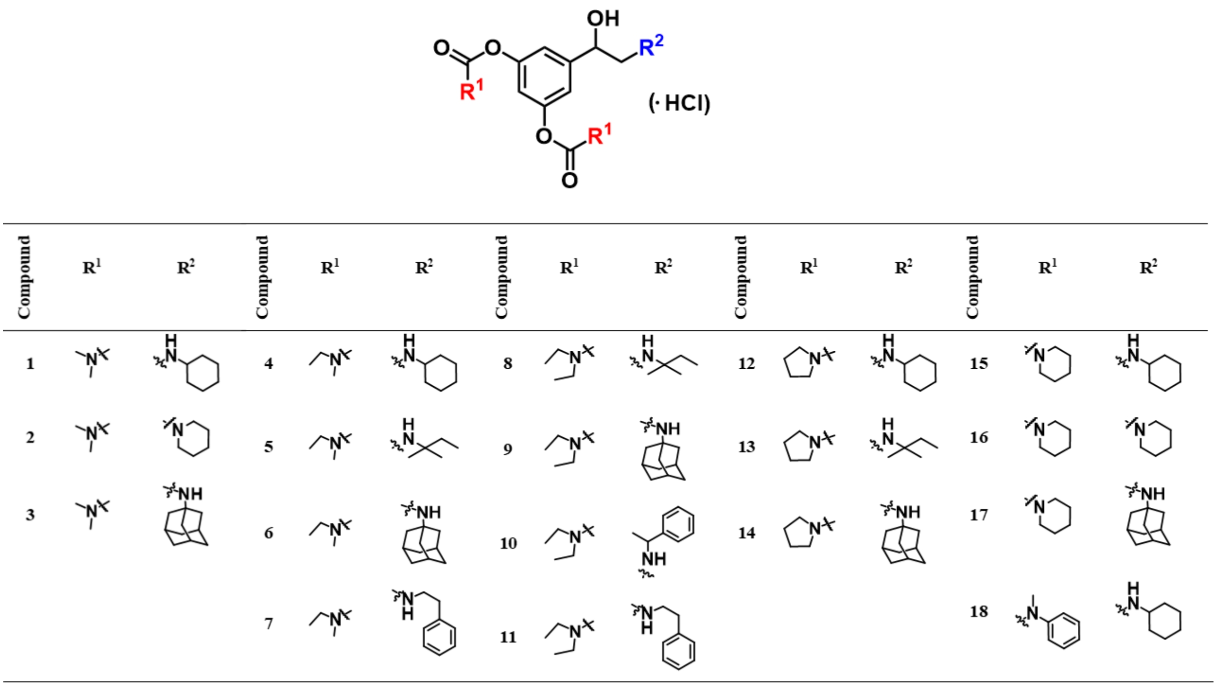


Figure S1. Structures of synthesized biscarbamates

**Table S1.** Decarbamylation rate constants ($k_{decarb}$) for BChE and AChE.

| Compound | $k_{decarb}$ [$h^{-1}$] | |
|---|---|---|
| | BChE | AChE |
| **1** | 0.162 ± 0.006 | 0.174 ± 0.018 |
| **2** | 0.132 ± 0.012 | 0.180±0.018 |
| **3** | 0.150 ± 0.012 | 0.138 ± 0.012 |
| **4** | 0.0960 ± 0.006 | 0.180 ± 0.018 |
| **5** | 0.192 ± 0.018 | 0.174 ± 0.012 |
| **6** | 0.0960 ± 0.0060 | 0.150 ± 0.012 |
| **7** | 0.126 ± 0.006 | 0.150 ± 0.012 |
| **8** | 0.0720 ±0.006 | 0.216 ± 0.018 |
| **9** | 0.0720 ± 0.006 | 0.180 ± 0.018 |
| **10** | 0.114 ± 0.006 | 0.222 ± 0.018 |
| **11** | 0.126 ± 0.018 | 0.204 ± 0.012 |
| **12** | 0.174 ± 0.006 | 0.204 ± 0.06 |
| **13** | 0.108 ± 0.006 | 0.180 ±0.012 |
| **14** | 0.102 ± 0.006 | 0.174 ± 0.024 |
| **15** | 0.162 ± 0.012 | 0.210 ± 0.018 |
| **16** | 0.126 ± 0.018 | 0.168 ± 0.012 |
| **17** | 0.168 ± 0.012 | 0.156 ± 0.012 |
| **18** | 0.120 ± 0.012 | 0.162 ± 0.018 |

**Table S2.** Physiochemical properties (molecular weight, MW; lipophilicity coefficient, logP; number of hydrogen bond donors, HBD, and acceptors, HBA; rotable bonds, RB; polar surface area, PSA) of biscarbamates

| Compound | MW/100 | logP | HBD | HBA | RB | PSA/10 |
|---|---|---|---|---|---|---|
| **1** | 4.299 | 2.143 | 2 | 4 | 8 | 9.134 |
| **2** | 4.159 | 1.577 | 1 | 4 | 7 | 8.255 |
| **3** | 4.820 | 2.435 | 2 | 4 | 8 | 9.134 |
| **4** | 4.580 | 2.856 | 2 | 4 | 10 | 9.134 |
| **5** | 4.460 | 2.633 | 2 | 4 | 11 | 9.134 |
| **6** | 5.107 | 3.148 | 2 | 4 | 10 | 9.134 |
| **7** | 4.800 | 3.070 | 2 | 4 | 12 | 9.134 |
| **8** | 4.740 | 3.347 | 2 | 4 | 13 | 9.134 |
| **9** | 5.381 | 3.862 | 2 | 4 | 12 | 9.134 |
| **10** | 5.081 | 3.912 | 2 | 4 | 13 | 9.134 |
| **11** | 5.081 | 3.784 | 2 | 4 | 14 | 9.134 |
| **12** | 4,820 | 2.954 | 2 | 4 | 8 | 9.134 |
| **13** | 4.701 | 2.731 | 2 | 4 | 9 | 9.134 |
| **14** | 5.341 | 3.246 | 2 | 4 | 8 | 9.134 |
| **15** | 5.101 | 3.843 | 2 | 4 | 8 | 9.134 |
| **16** | 4.961 | 3.278 | 1 | 4 | 7 | 8.255 |
| **17** | 5.622 | 4.136 | 2 | 4 | 8 | 9.134 |
| **18** | 5.541 | 5.459 | 2 | 6 | 10 | 9.134 |
| Bambuterol | 4.039 | 1.397 | 2 | 4 | 8 | 9.134 |
| Rivastigmine | 2.500 | 2.400 | 0 | 2 | 5 | 3.270 |
| Recommended values | 5 | 5 | 5 | 10 | 10 | 9 |

**1**

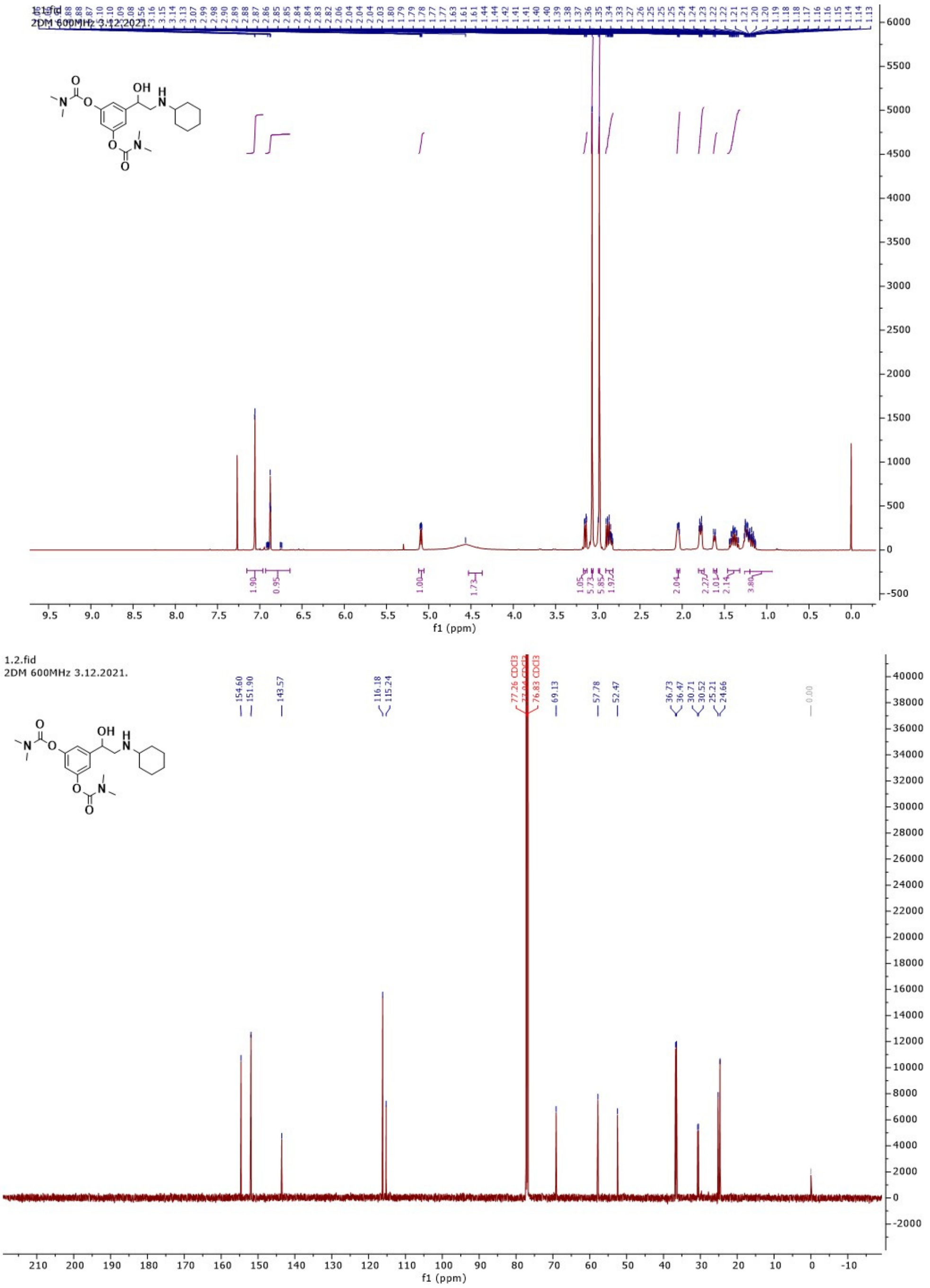


**Figure S2**. $^{1}$H and $^{13}$C spectra of the compound **1**

**2**

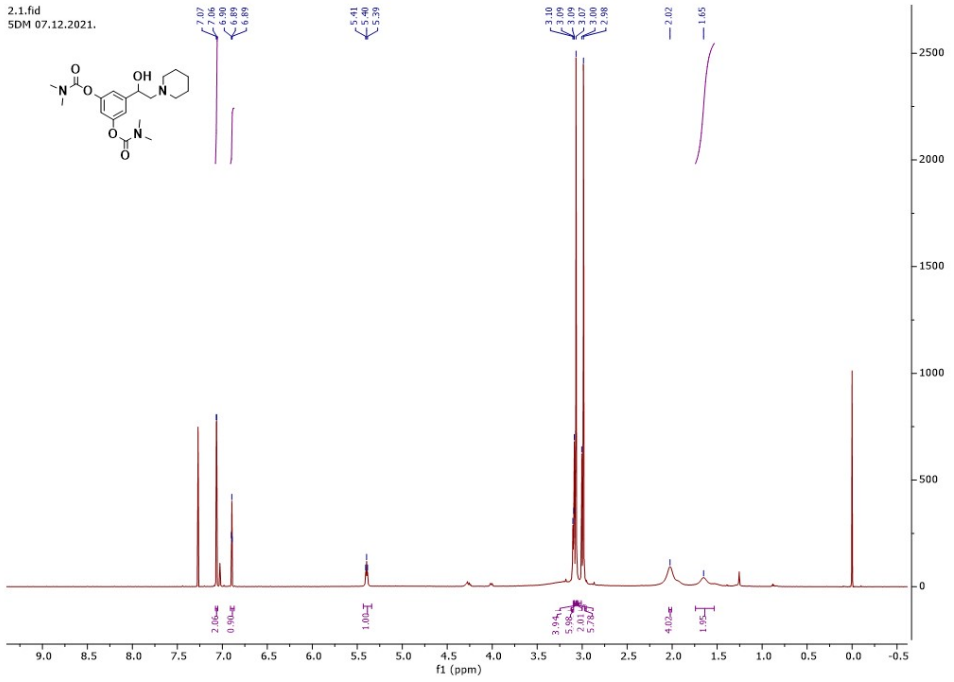


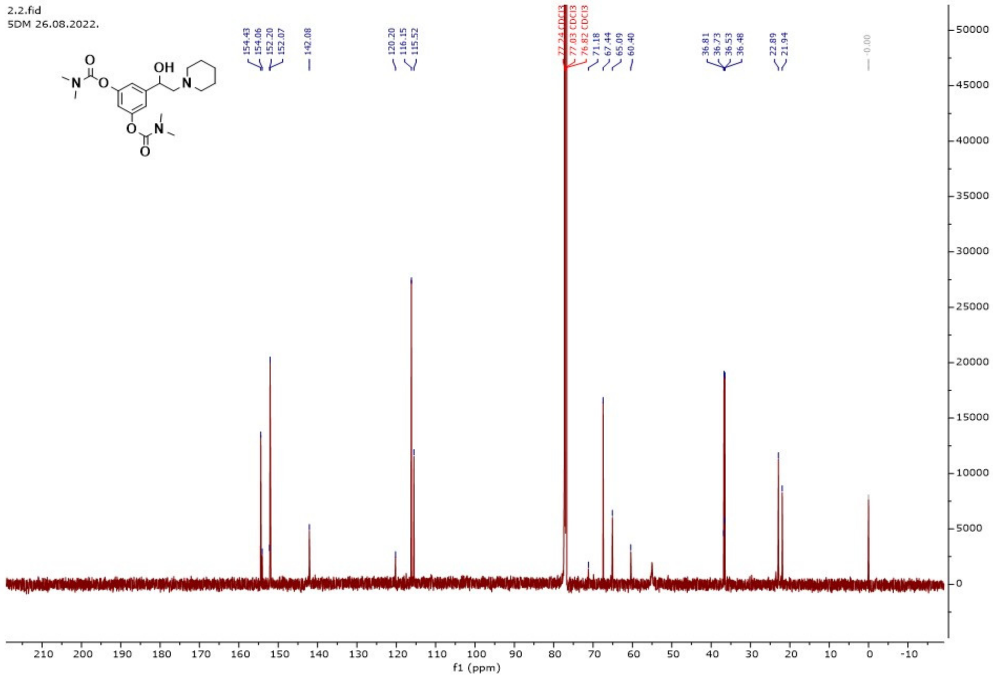


**Figure S3**. $^{1}$H and $^{13}$C spectra of the compound **2**

**3**

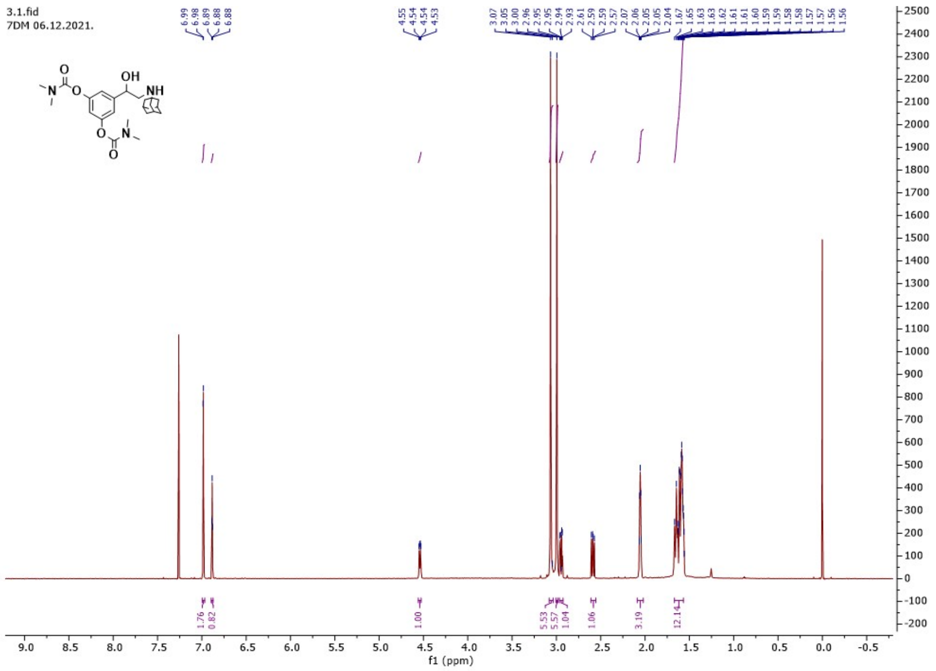


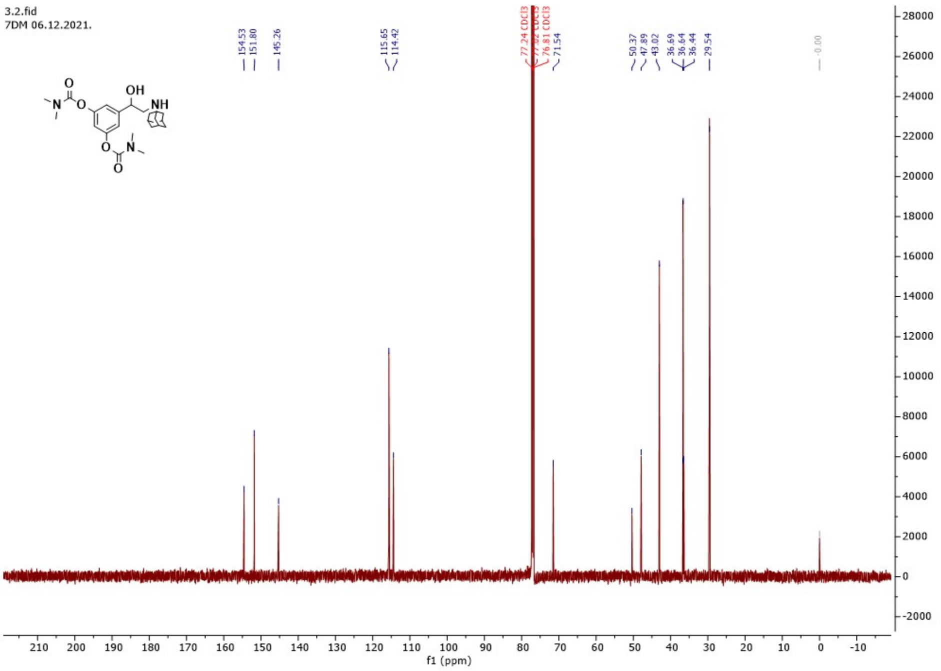


**Figure S4**. $^{1}$H and $^{13}$C spectra of the compound **3**

**4**

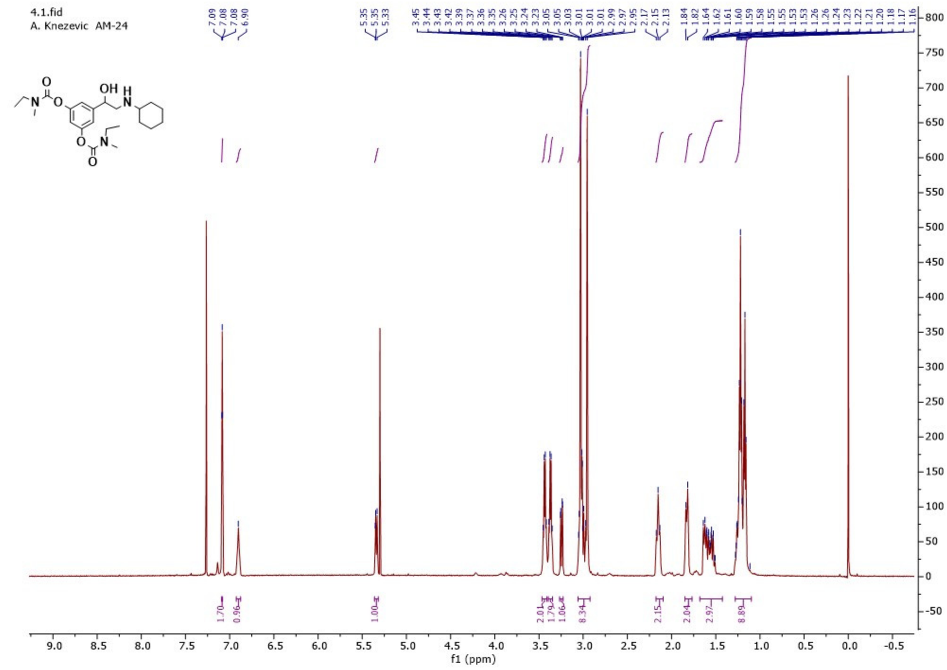


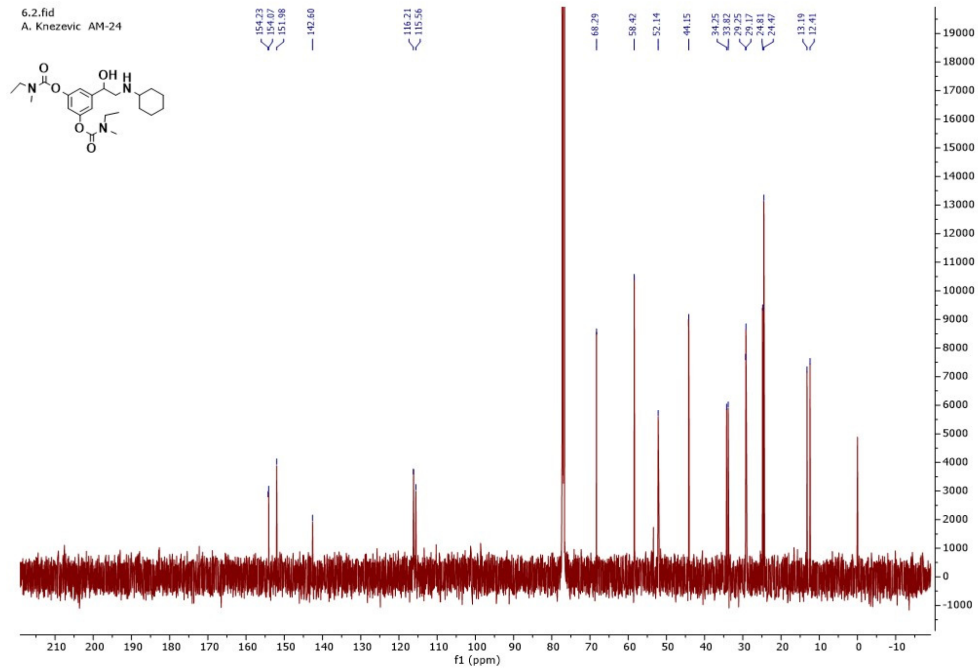


**Figure S5**. $^{1}$H and $^{13}$C spectra of the compound **4**

**5**

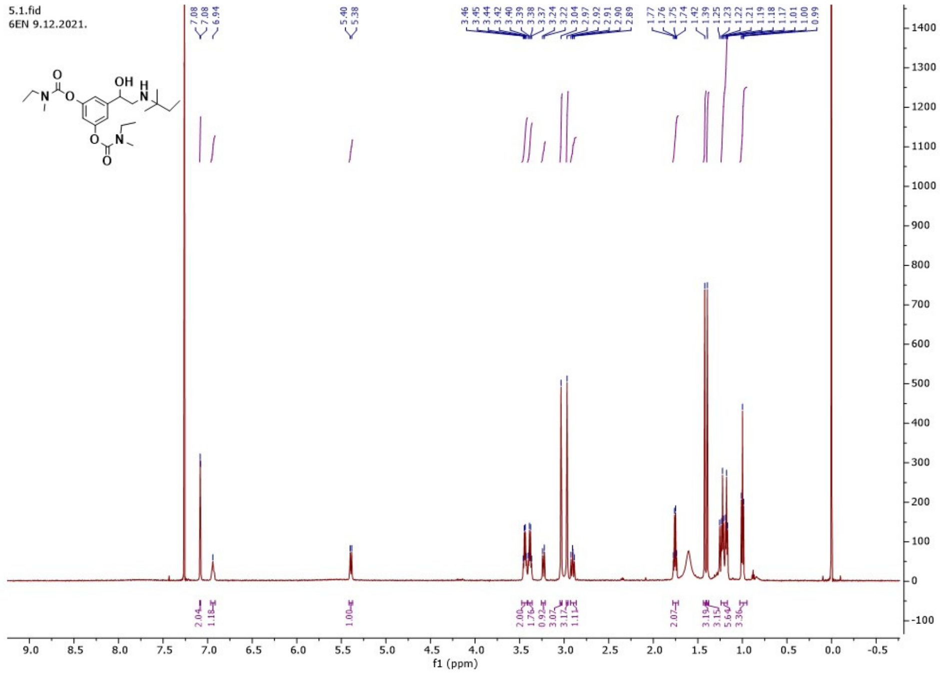

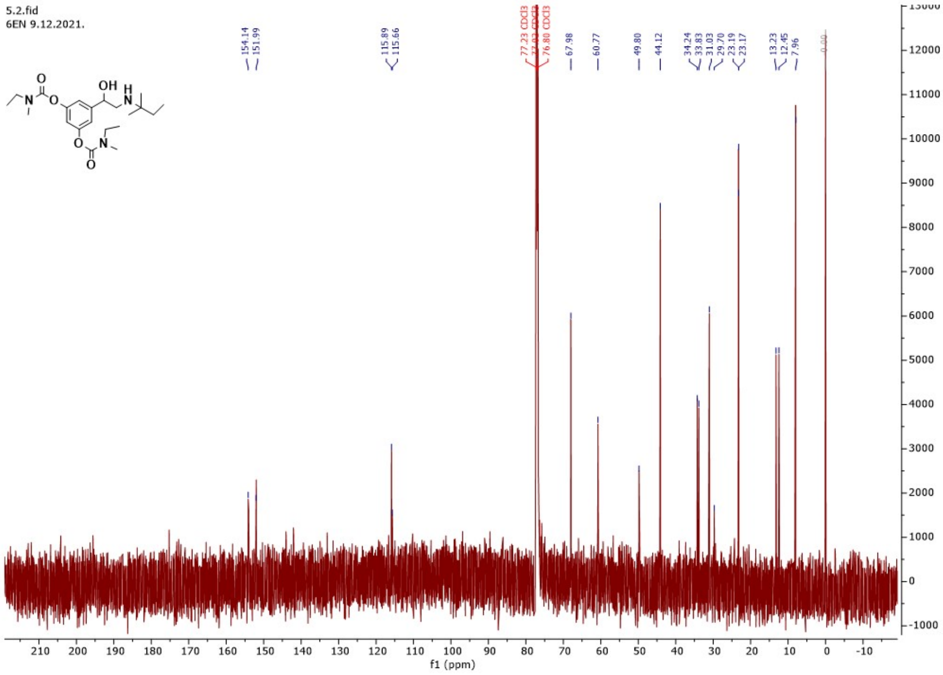

**Figure S6**. $^{1}$H and $^{13}$C spectra of the compound **5**

**6**

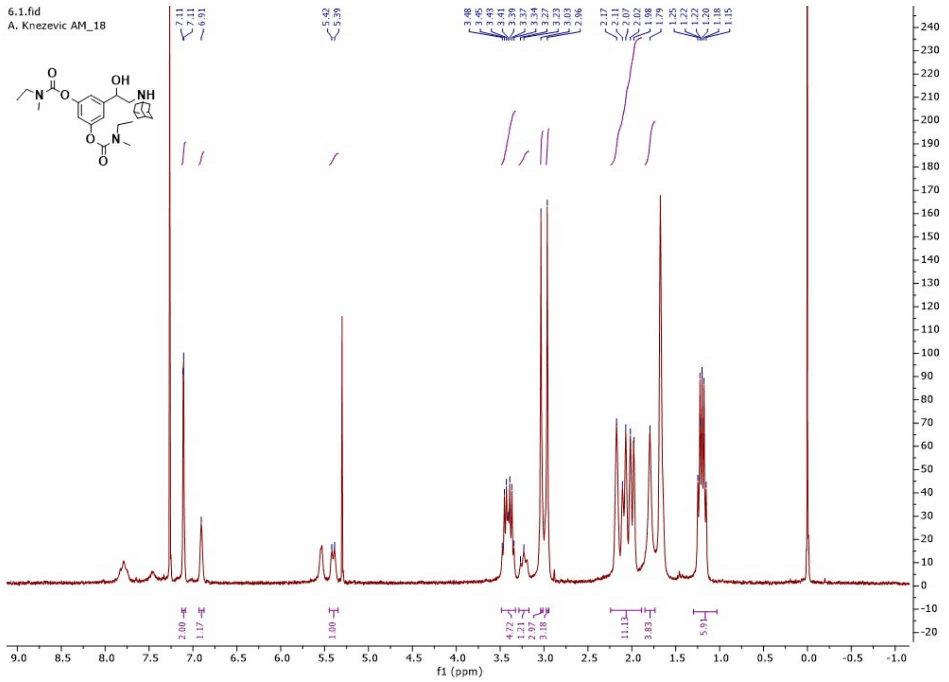


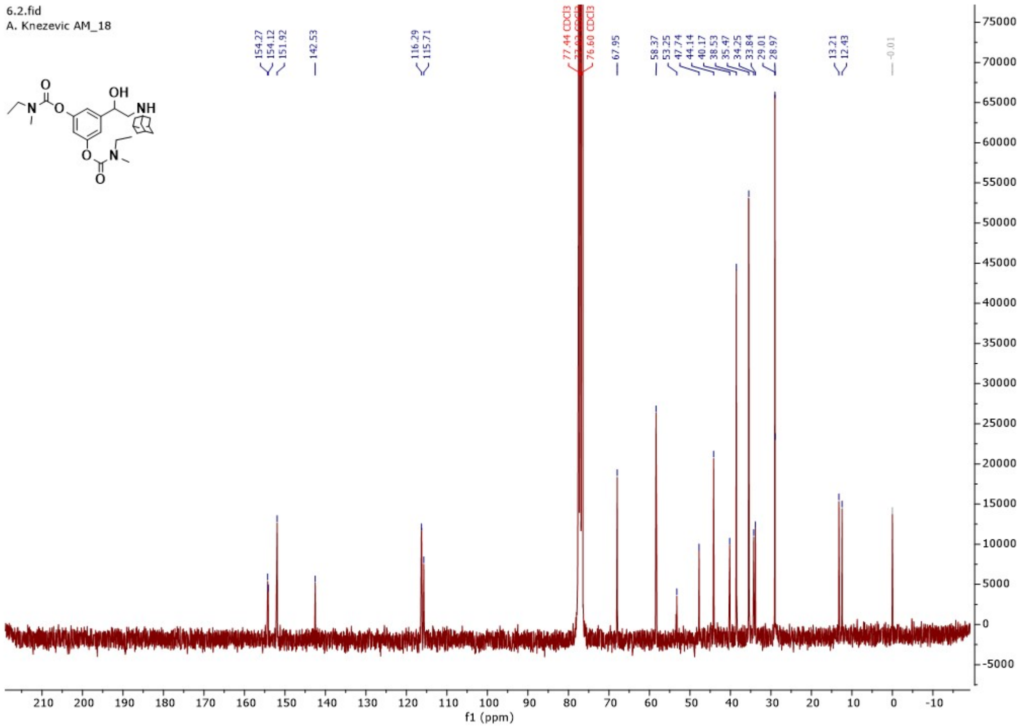


**Figure S7**. $^{1}H$ and $^{13}C$ spectra of the compound **6**

**7**

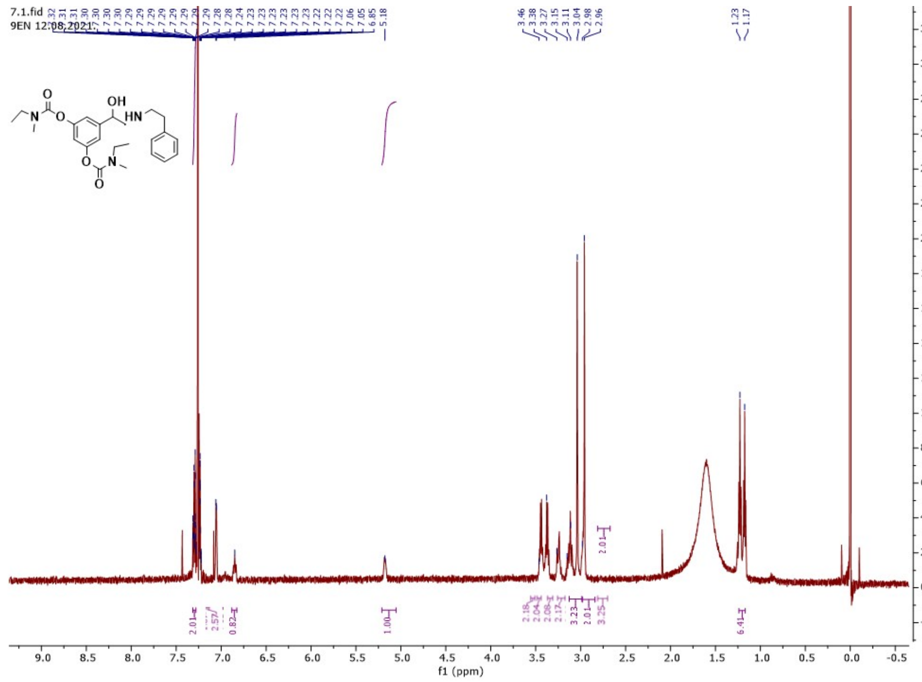


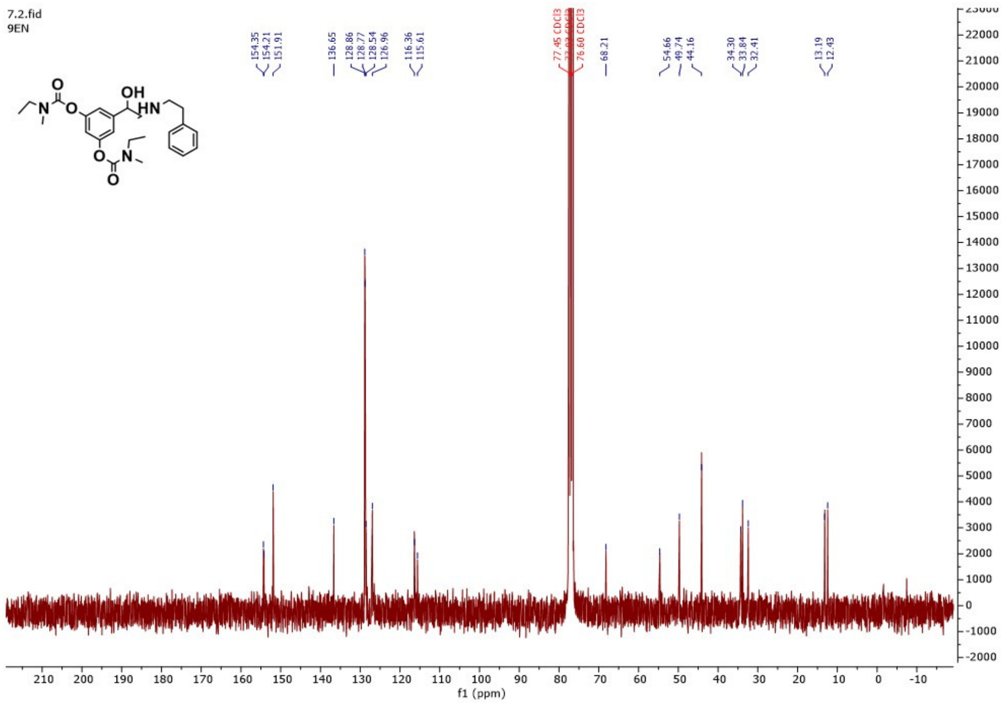


**Figure S8**. $^{1}H$ and $^{13}C$ spectra of the compound **7**

**8**

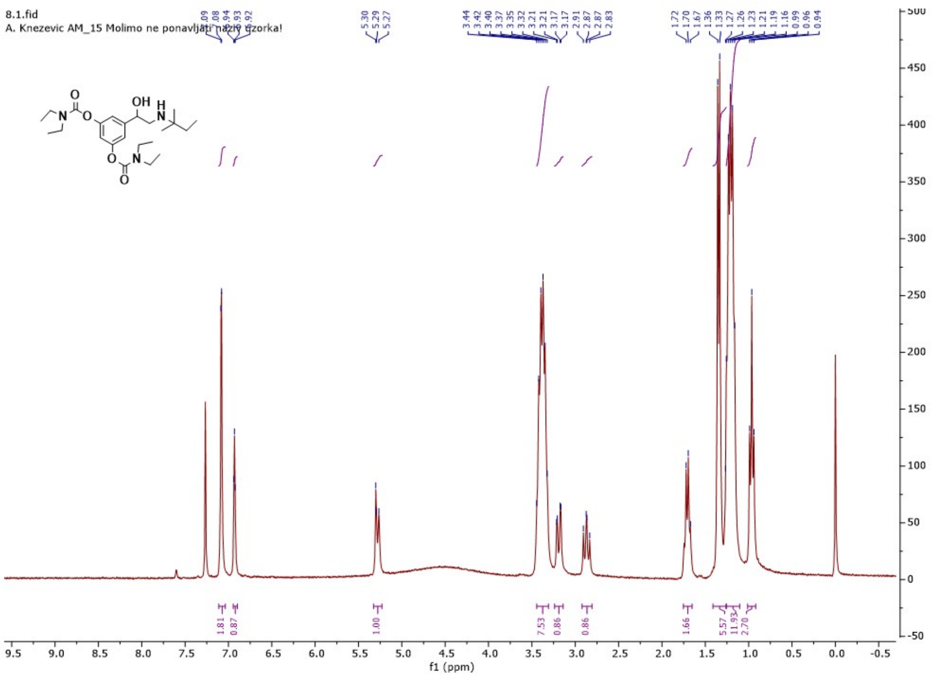


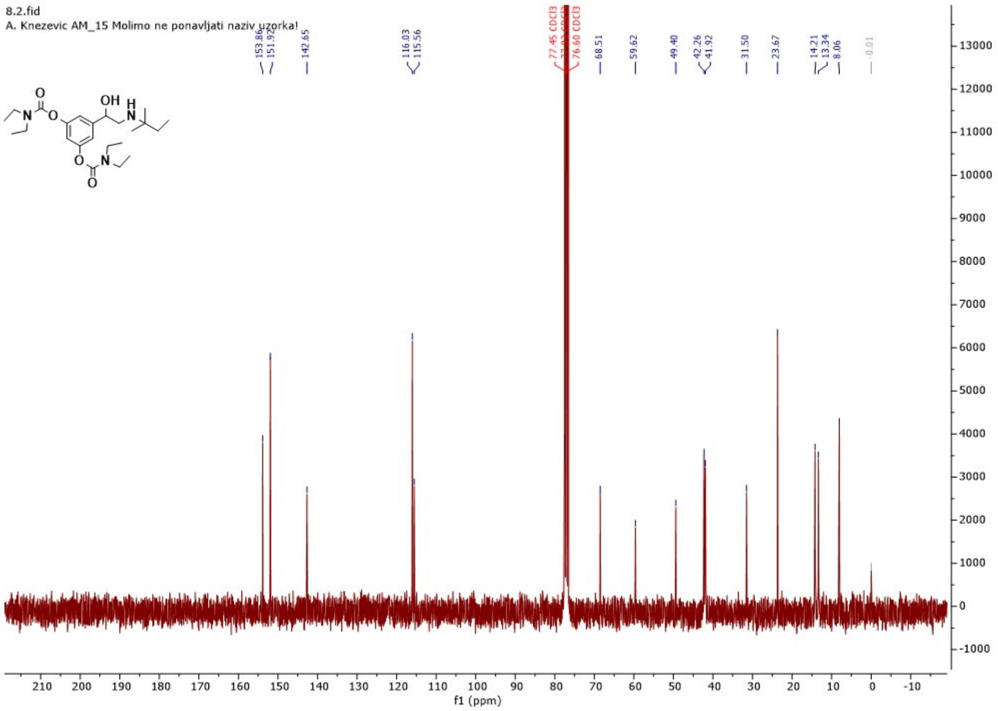


**Figure S9**. $^{1}$H and $^{13}$C spectra of the compound **8**

**9**

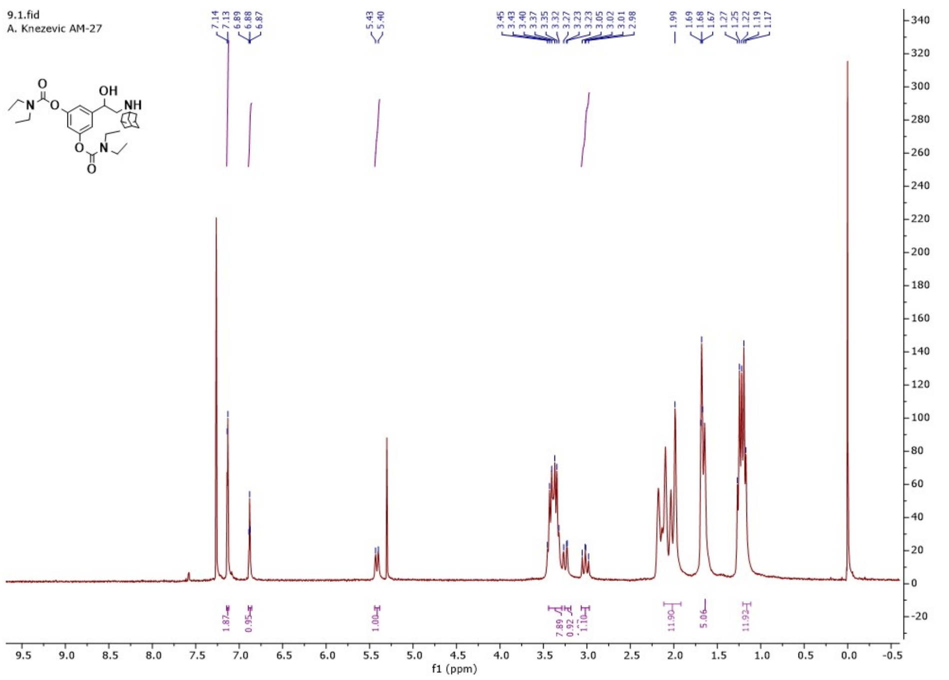

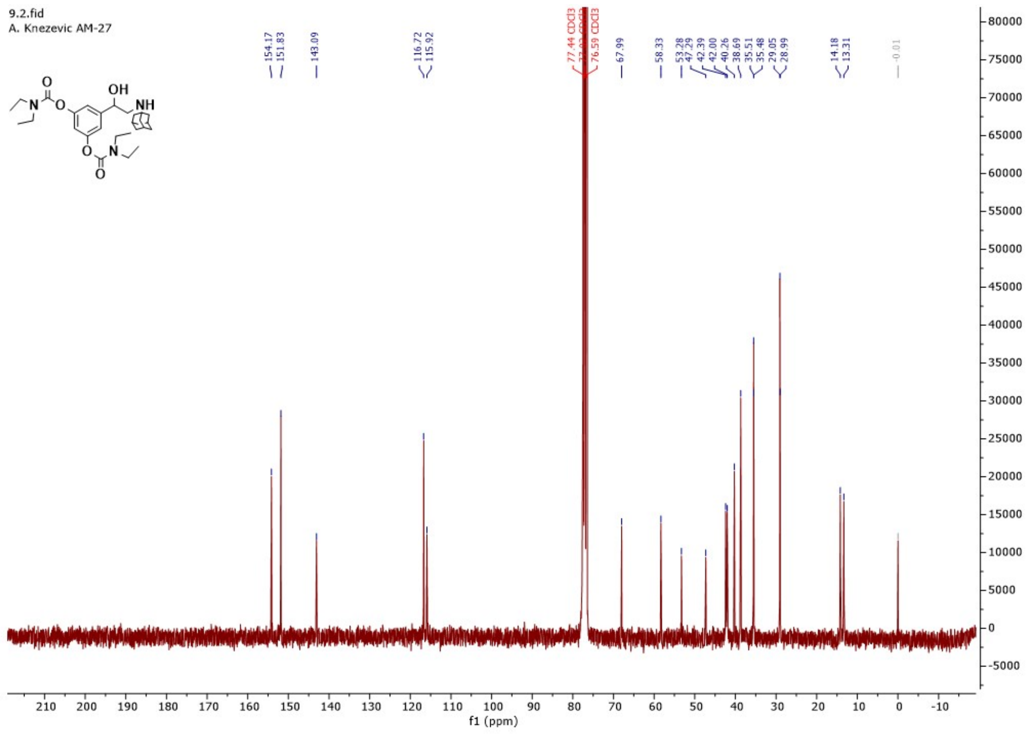

**Figure S10**. $^{1}$H and $^{13}$C spectra of the compound **9**

**10**

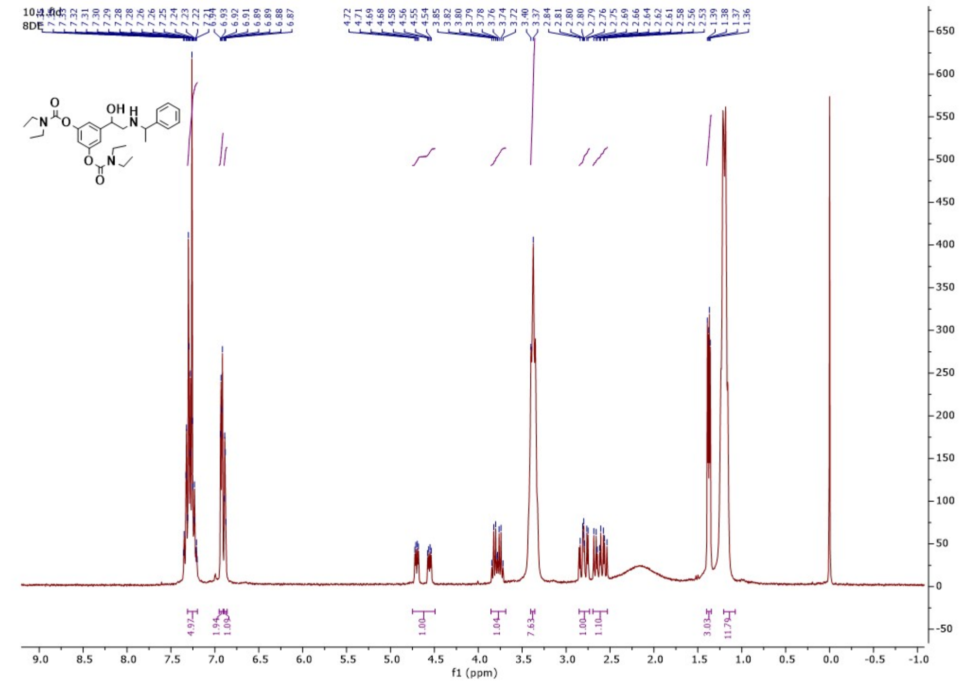

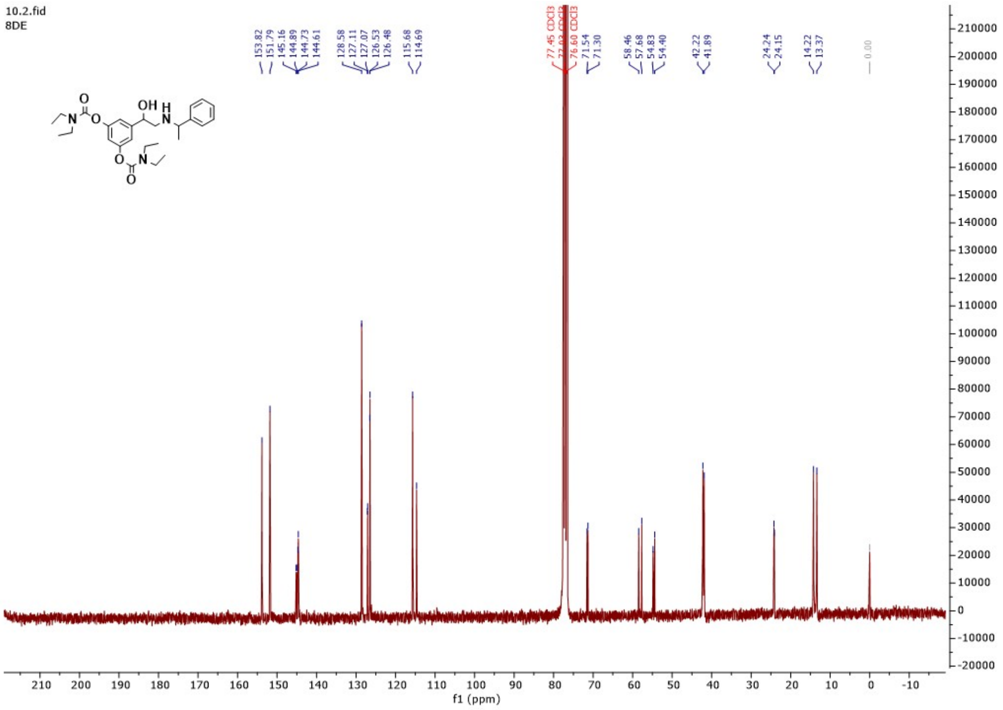

**Figure S11**. $^{1}$H and $^{13}$C spectra of the compound **10**

**11**

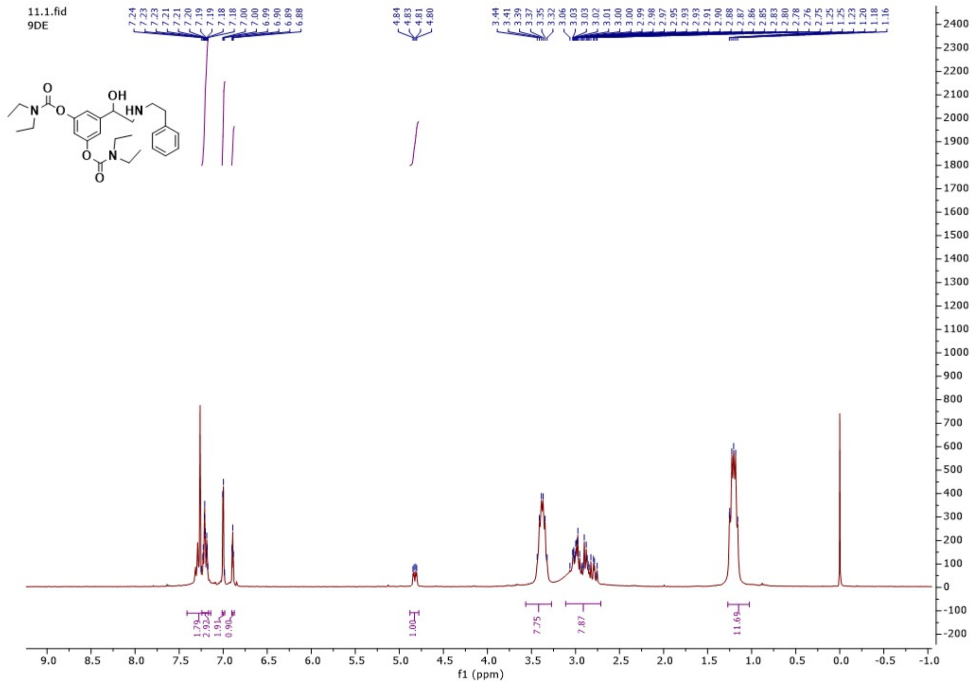


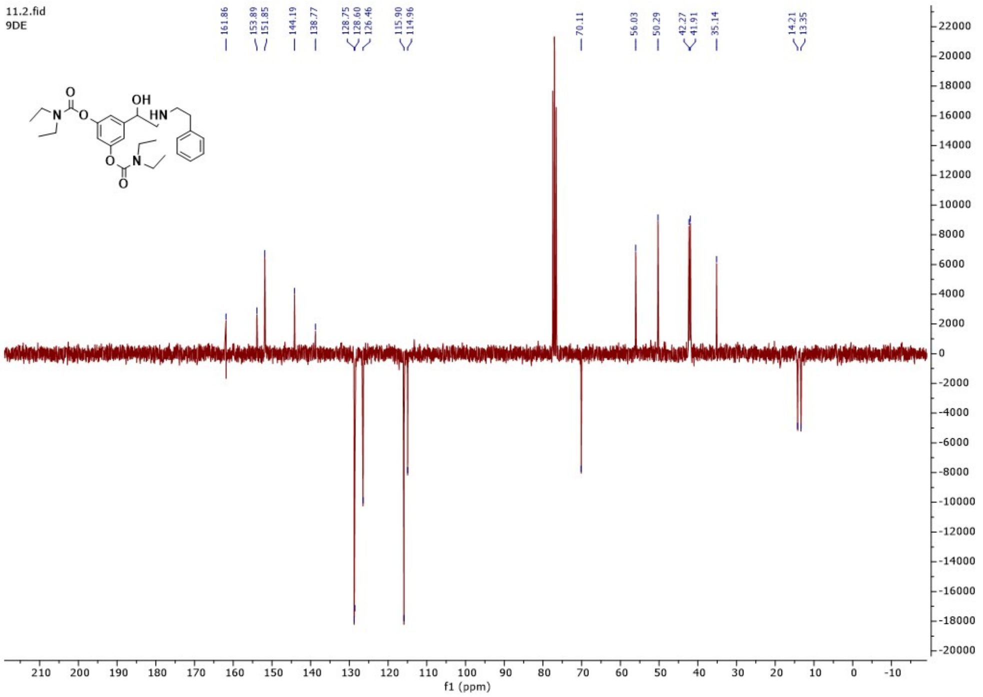


**Figure S12**. $^{1}$H and $^{13}$C spectra of the compound **11**

**12**

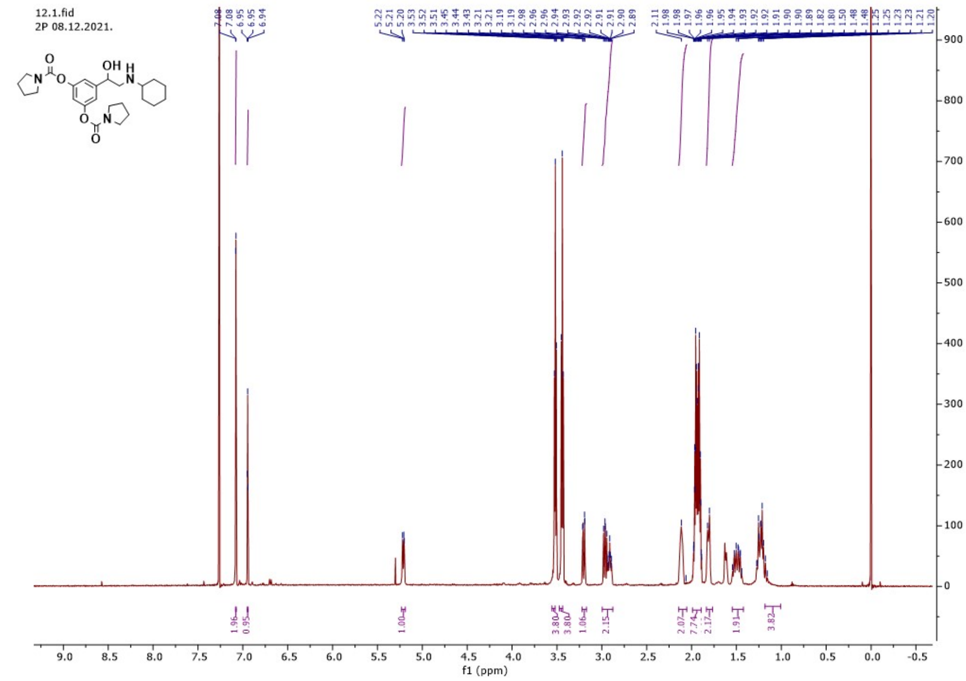


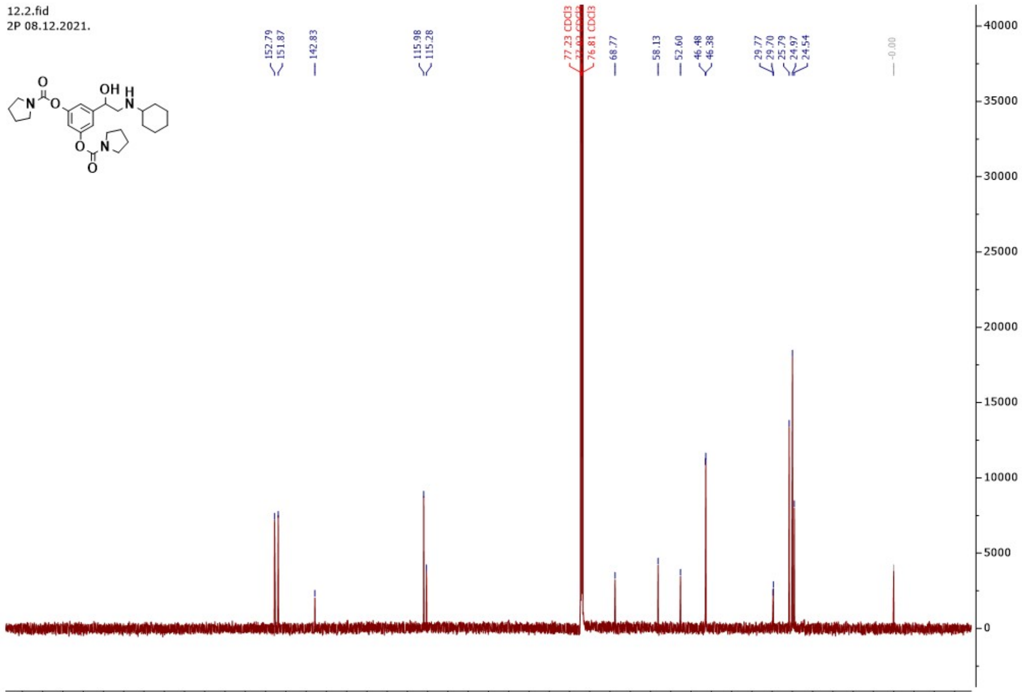


**Figure S13**. $^{1}H$ and $^{13}C$ spectra of the compound **12**

**13**

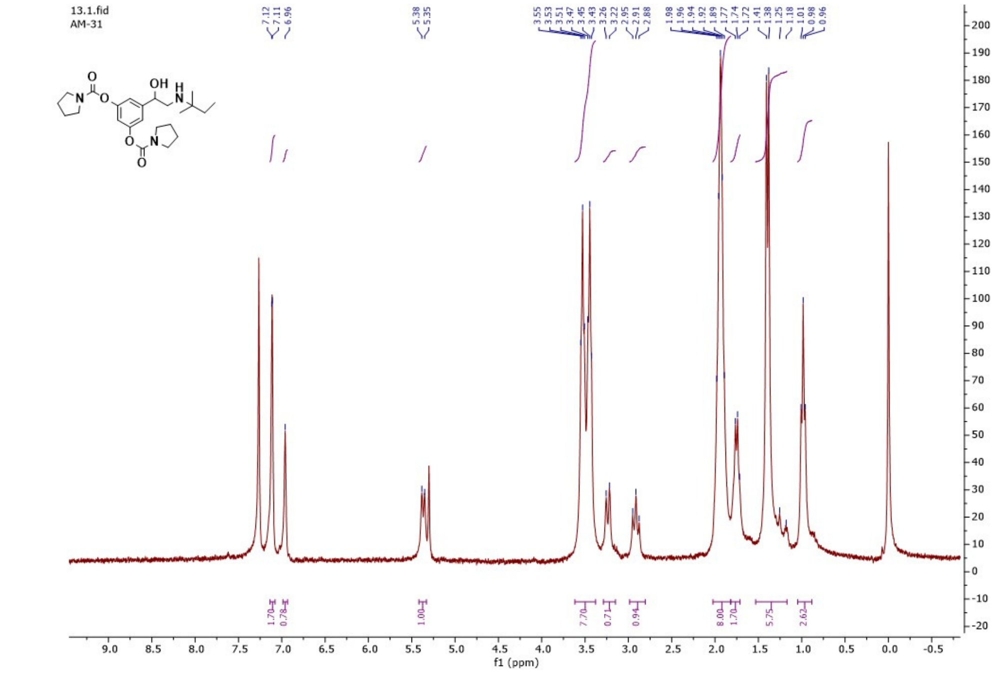


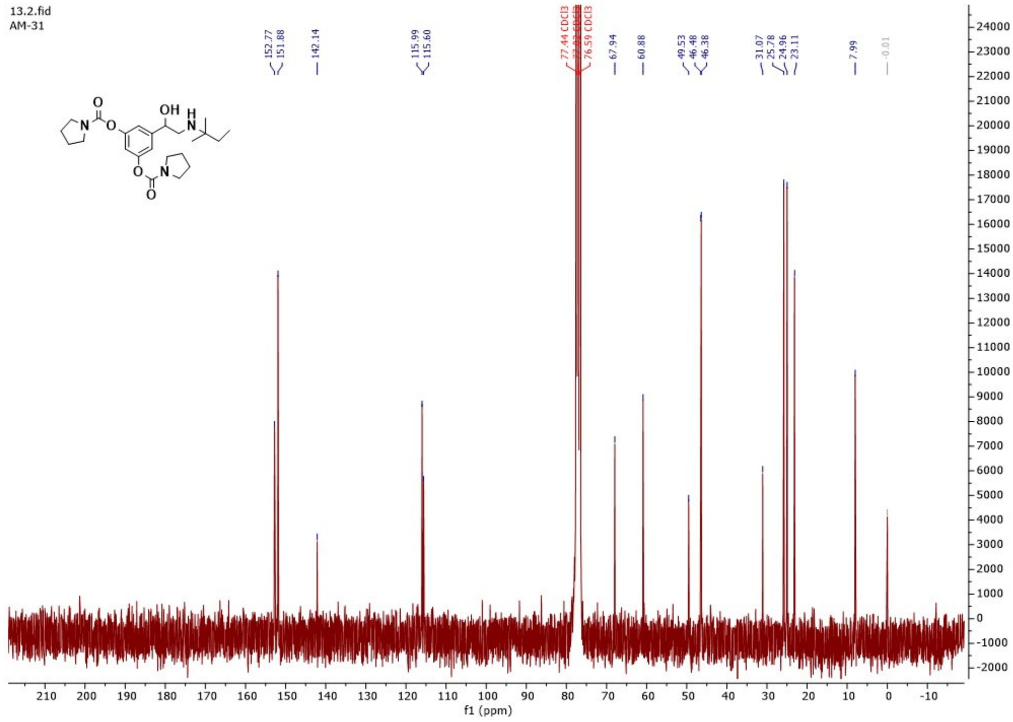


**Figure S14**. $^{1}H$ and $^{13}C$ spectra of the compound **13**

**14**

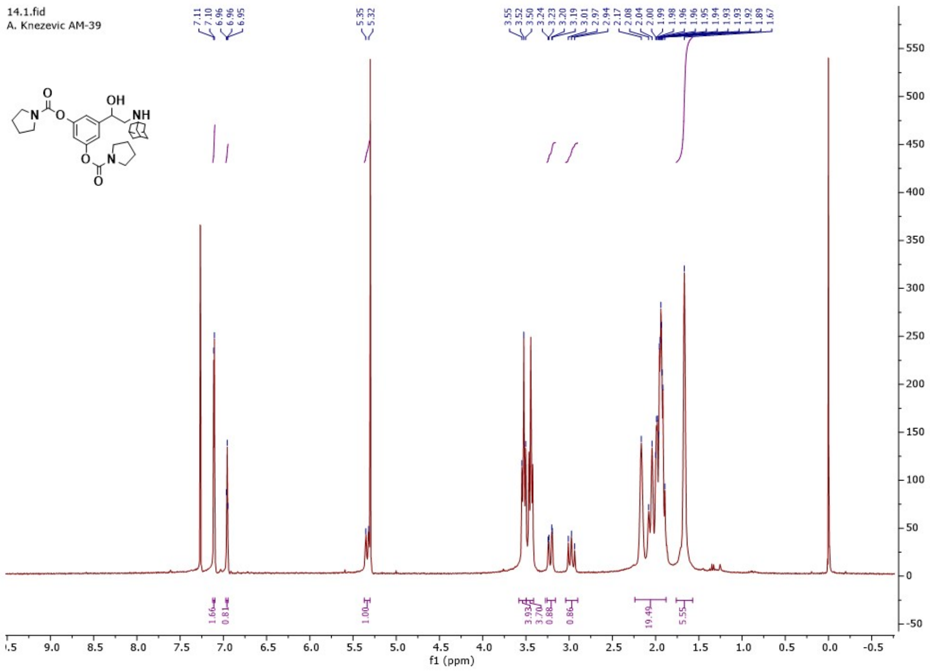


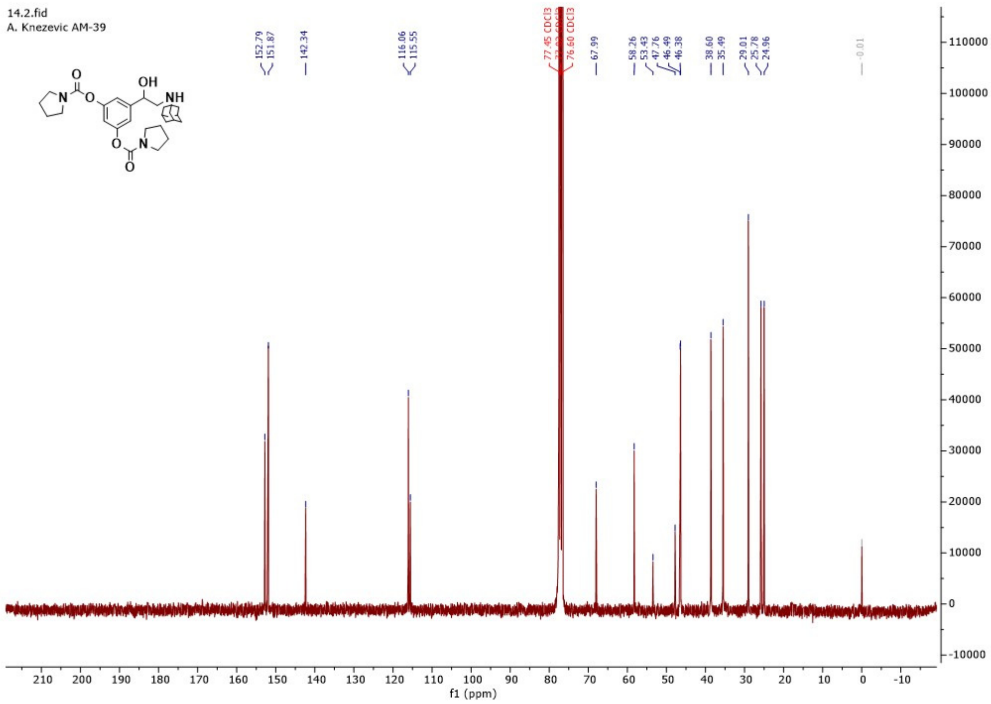


**Figure S15**. $^{1}H$ and $^{13}C$ spectra of the compound **14**

**15**

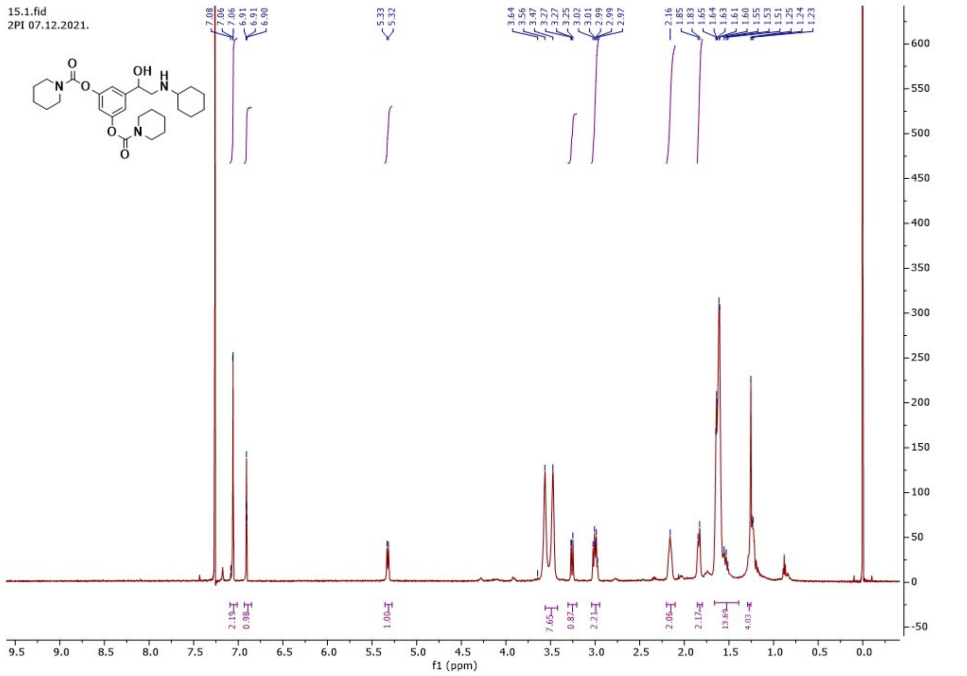

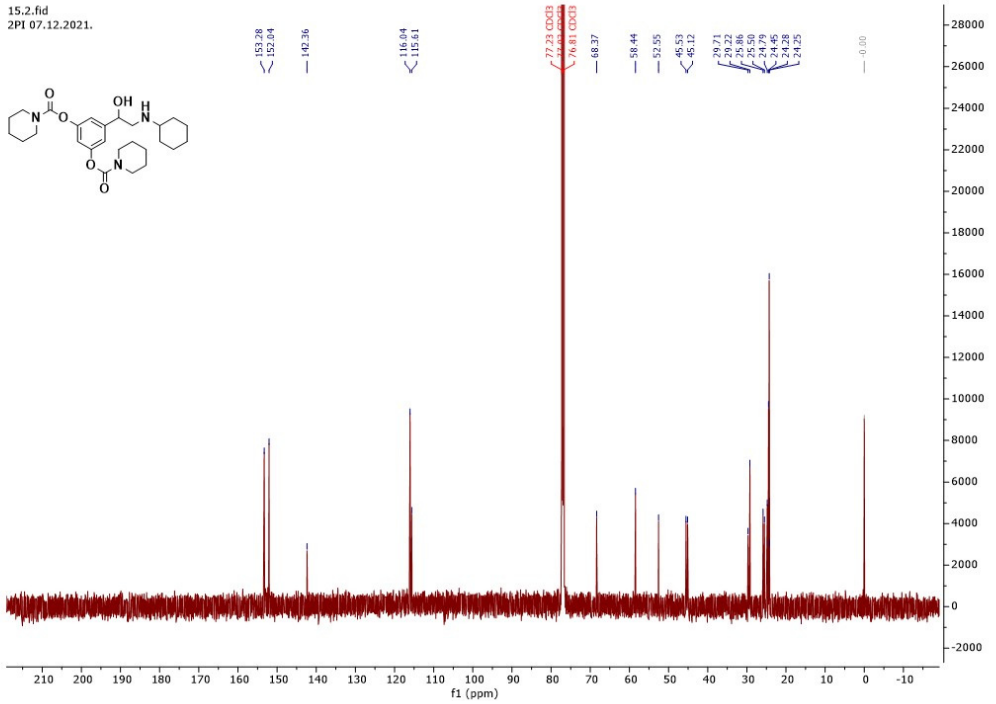

**Figure S16**. $^{1}$H and $^{13}$C spectra of the compound **15**

**16**

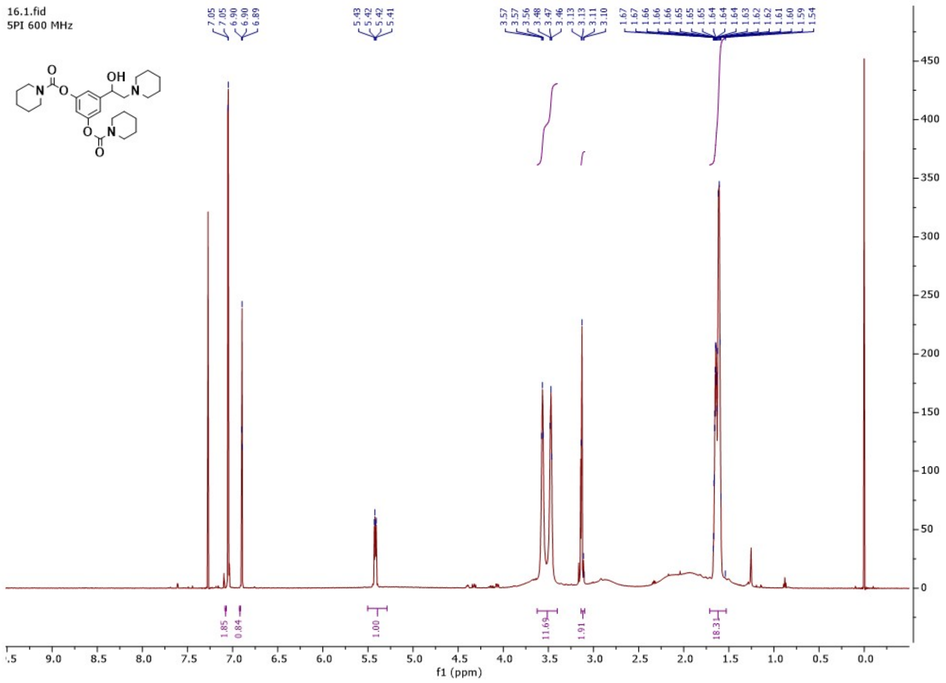


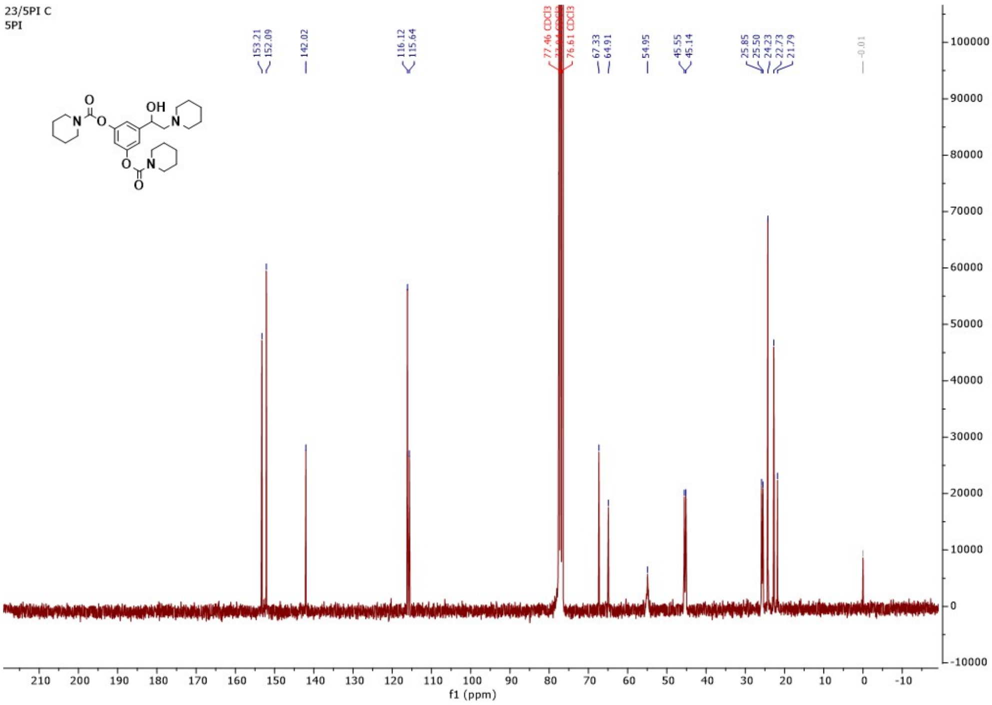


**Figure 17**. $^{1}H$ and $^{13}C$ spectra of the compound **16**

**17**

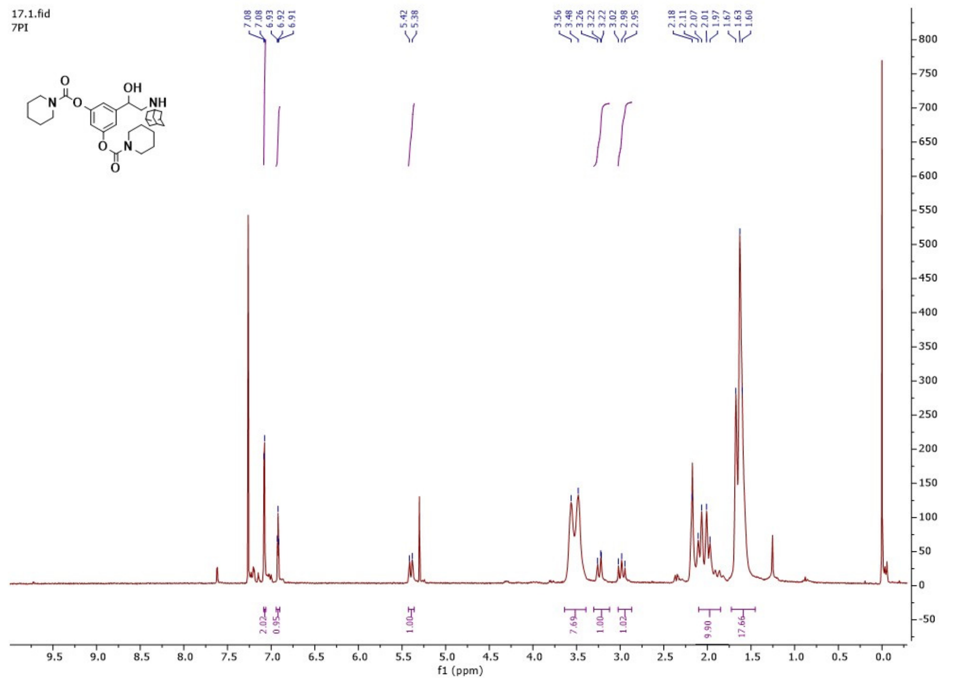


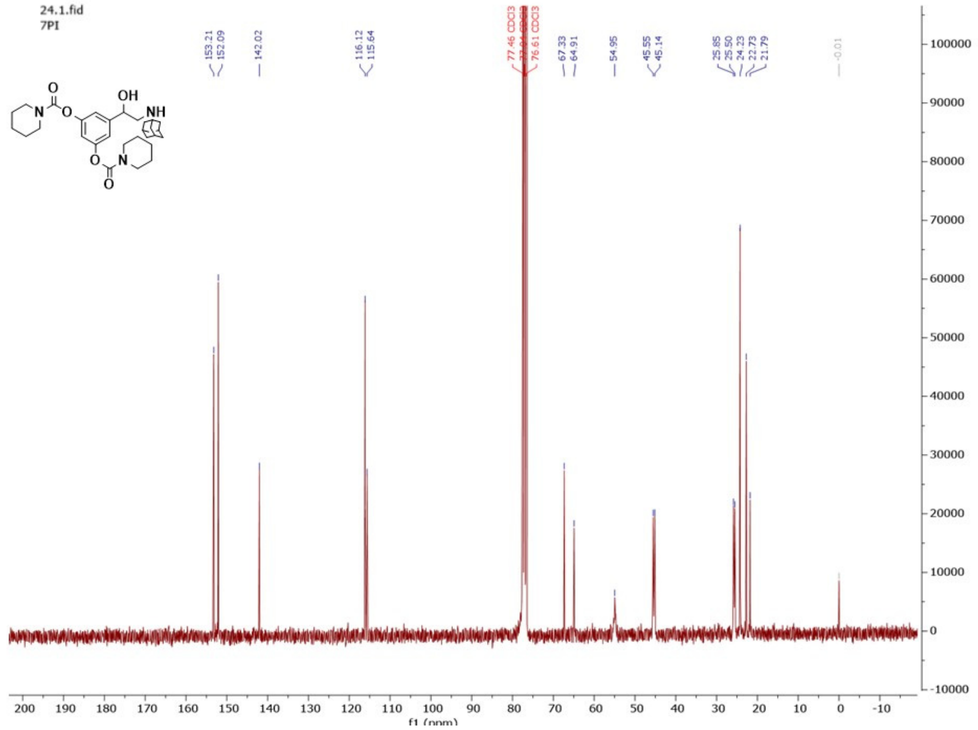


**Figure S17**. $^{1}$H and $^{13}$C spectra of the compound **18**

**18**

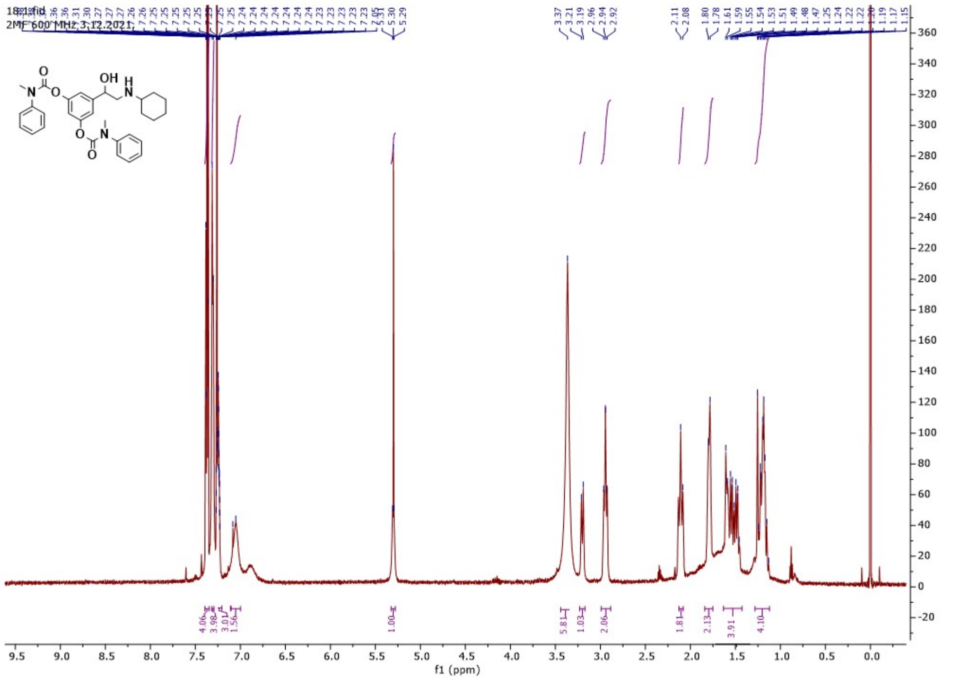

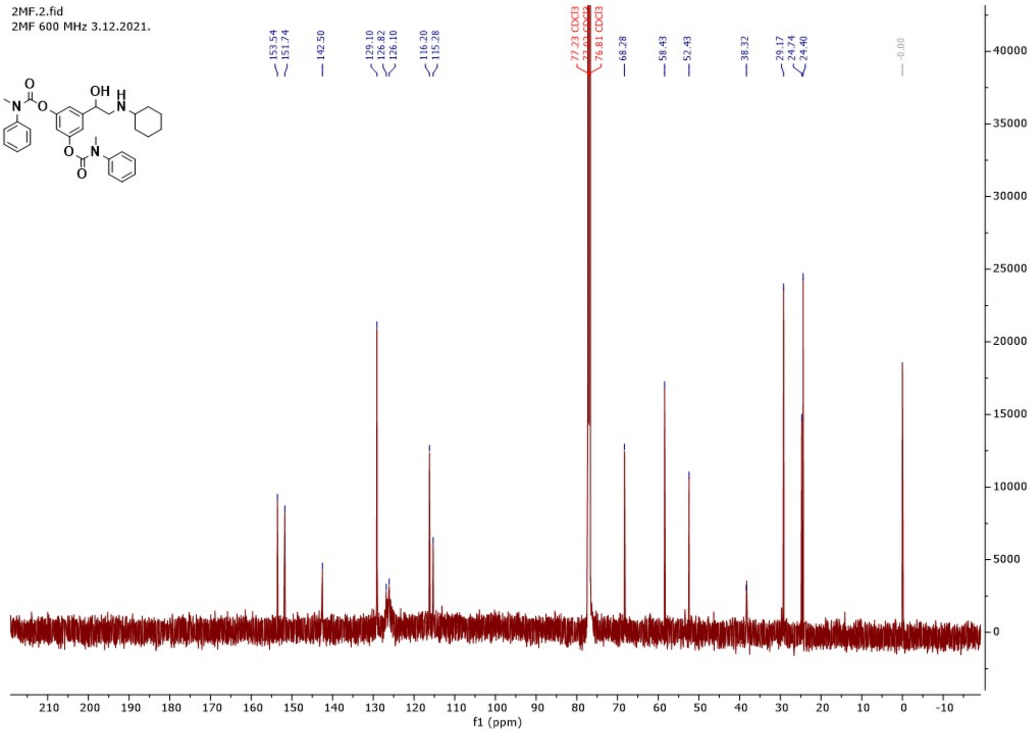


**Figure S19**. $^{1}H$ and $^{13}C$ spectra of the compound **18**

**1**

Molecular formula: $C_{20}H_{31}N_3O_5$

Theoretical monoisotropic mass $[M+H]^+$: 394.2342

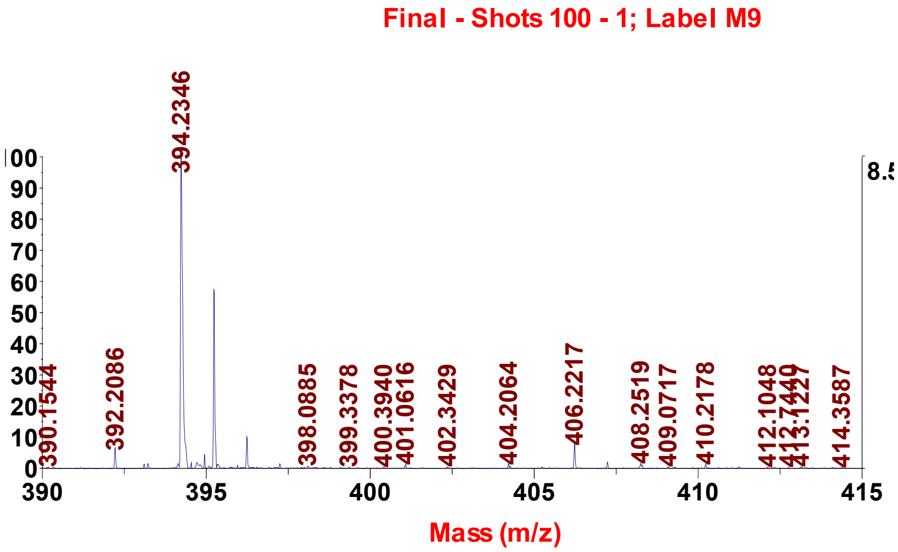


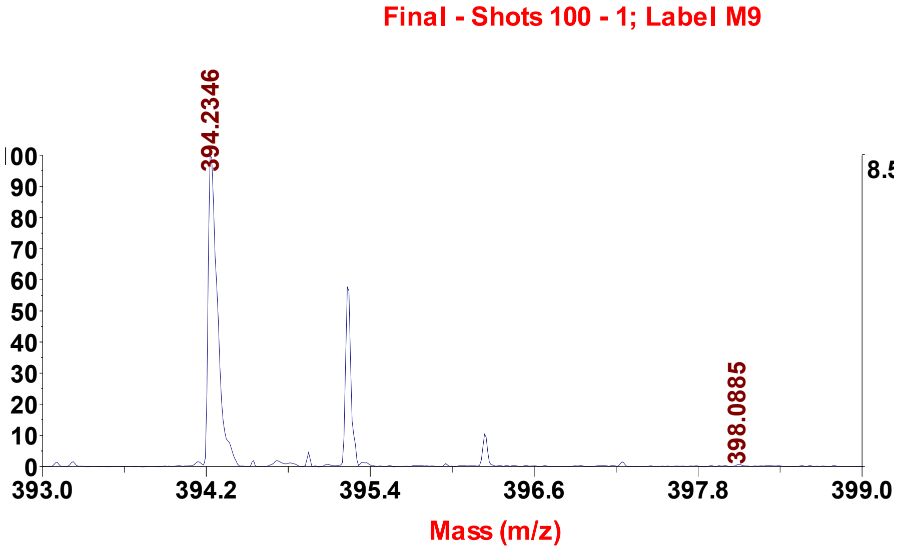


Figure S20. Mass spectar of **1** in the area *m/z* 390 – 415 (upper spectrum) and its enlarged view in the area *m/z* 393 – 399 (lower spectrum).

**2**

Molecular formula: $C_{19}H_{29}N_3O_5$

Theoretical monoisotropic mass $[M+Na]^+$: 402.2005

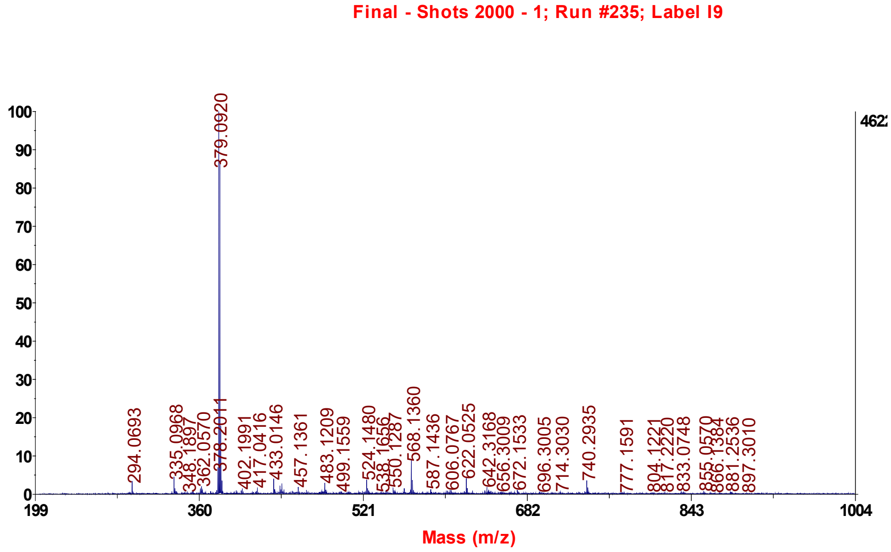


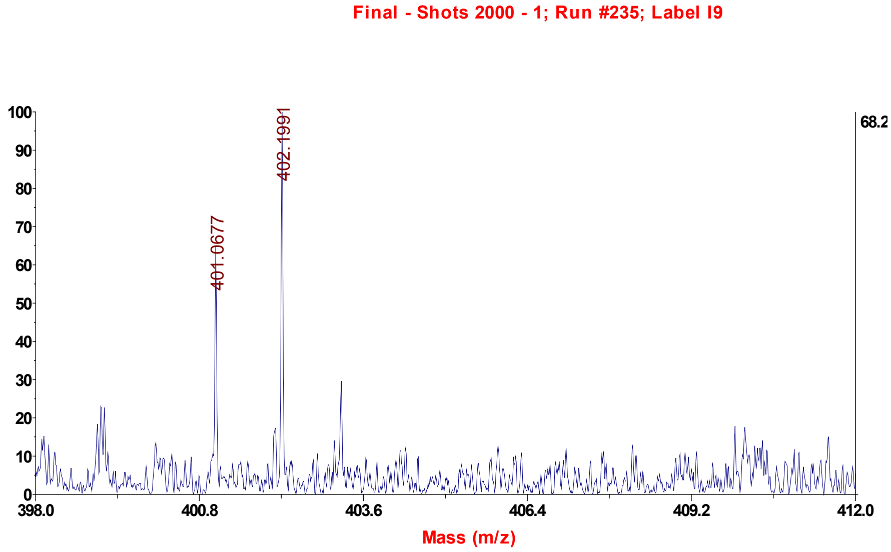


Figure S21. Mass spectar of **2** in the area *m/z* 199 – 1004 (upper spectrum) and its enlarged view in the area *m/z* 398 – 412 (lower spectrum).

**3**

Molecular formula: $C_{24}H_{35}N_3O_5$

Theoretical monoisotropic mass $[M+H]^+$: 446.2655

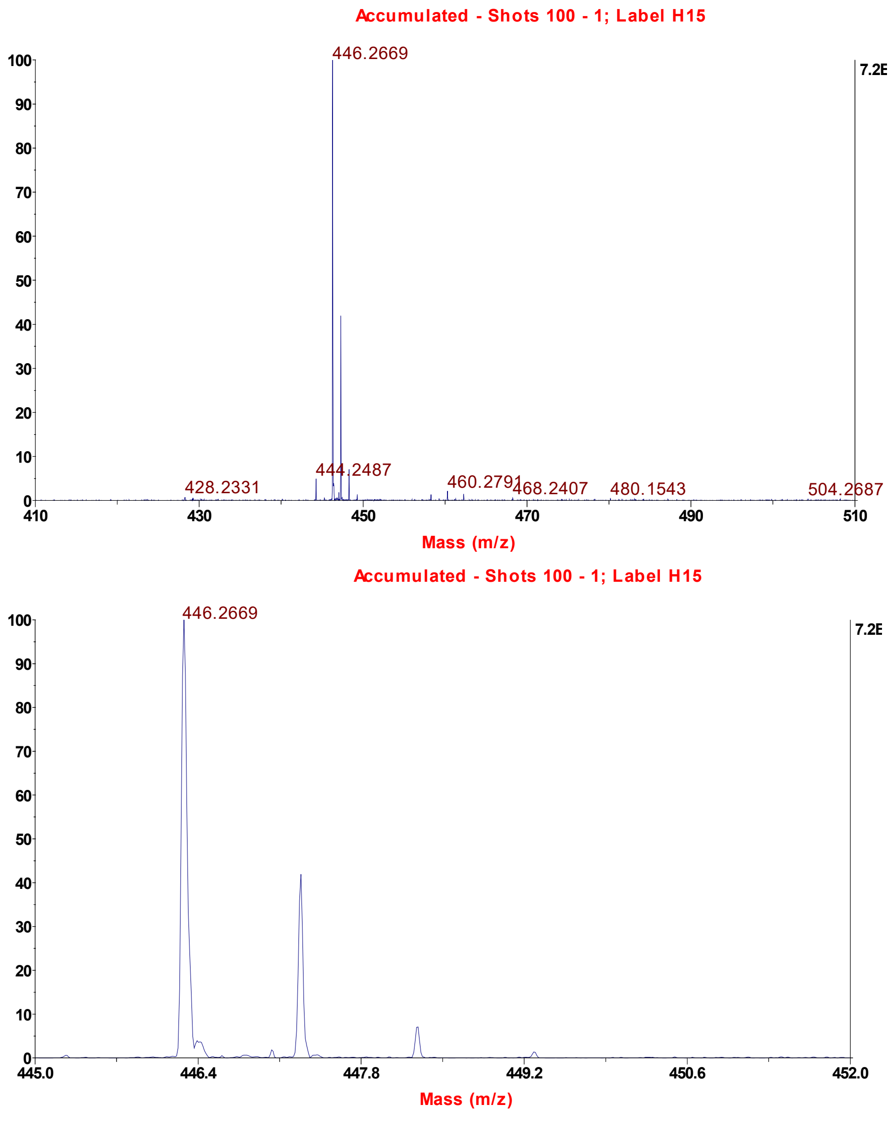


Figure S22. Mass spectar of **3** in the area *m/z* 410 – 510 (upper spectrum) and its enlarged view in the area *m/z* 445 – 452 (lower spectrum).

**4**

Molecular formula: $C_{22}H_{35}N_3O_5$

Theoretical monoisotropic mass $[M+H]^+$: 422.2655

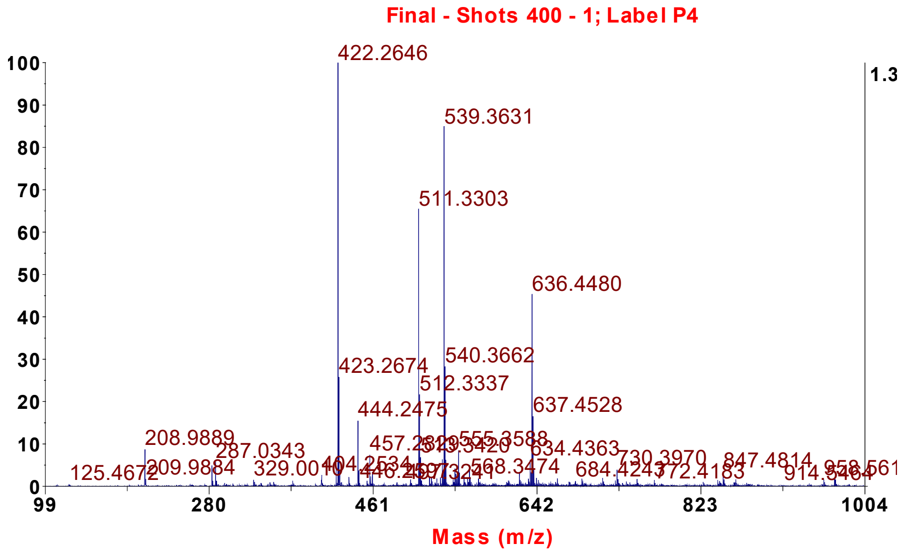


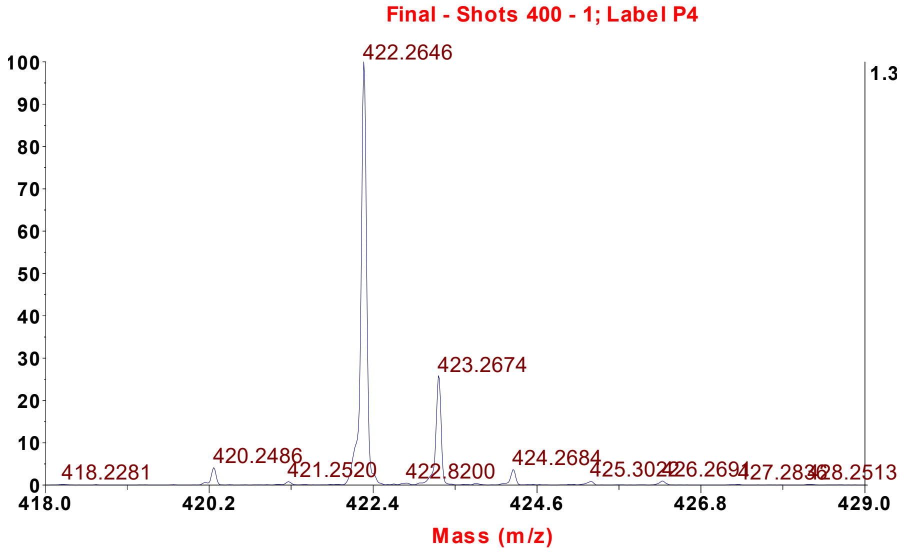


Figure S23. Mass spectar of **4** in the area *m/z* 99 – 1004 (upper spectrum) and its enlarged view in the area *m/z* 418 – 429 (lower spectrum).

5

Molecular formula: $C_{21}H_{36}N_3O_5$

Theoretical monoisotropic mass $[M+H]^+$: 410.2655

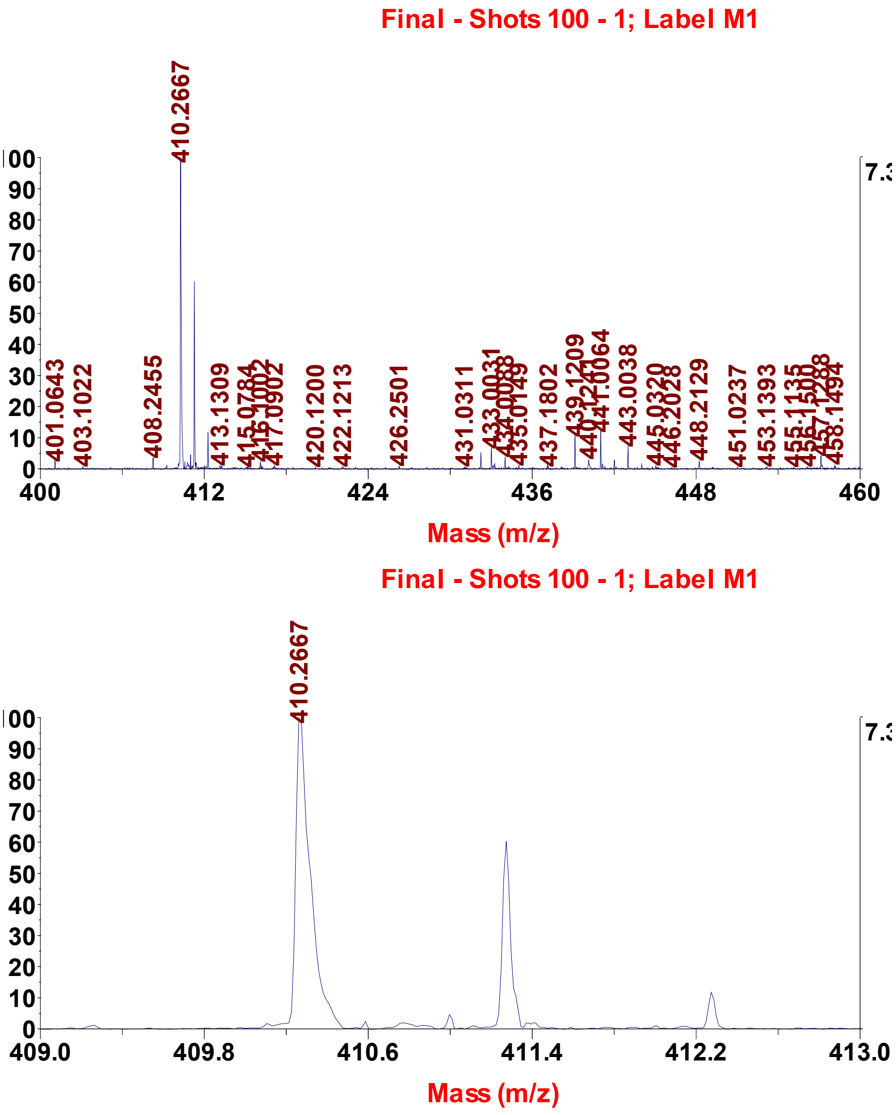


Figure S24. Mass spectar of **5** in the area *m/z* 400 – 460 (upper spectrum) and its enlarged view in the area *m/z* 409 – 413 (lower spectrum).

**6**

Molecular formula: $C_{26}H_{39}N_3O_5$

Theoretical monoisotropic mass $[M+H]^+$: 474.2968

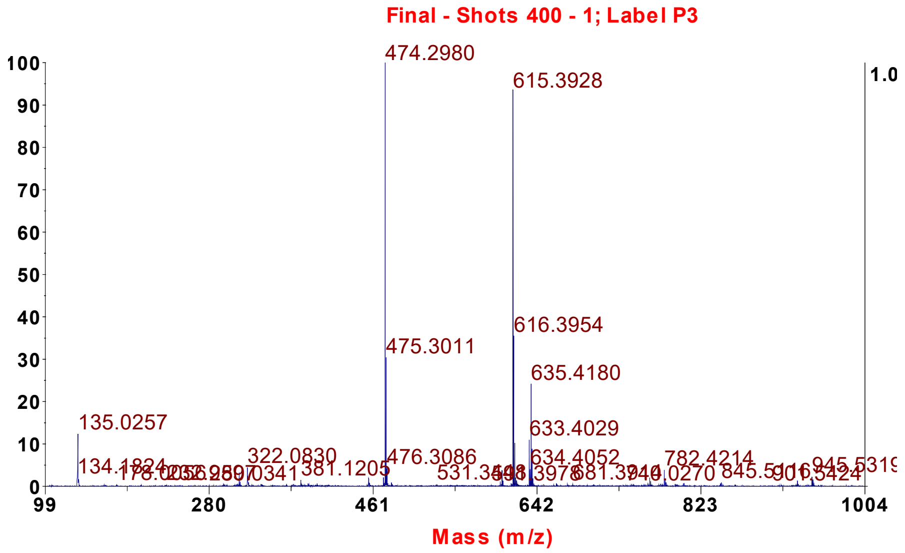


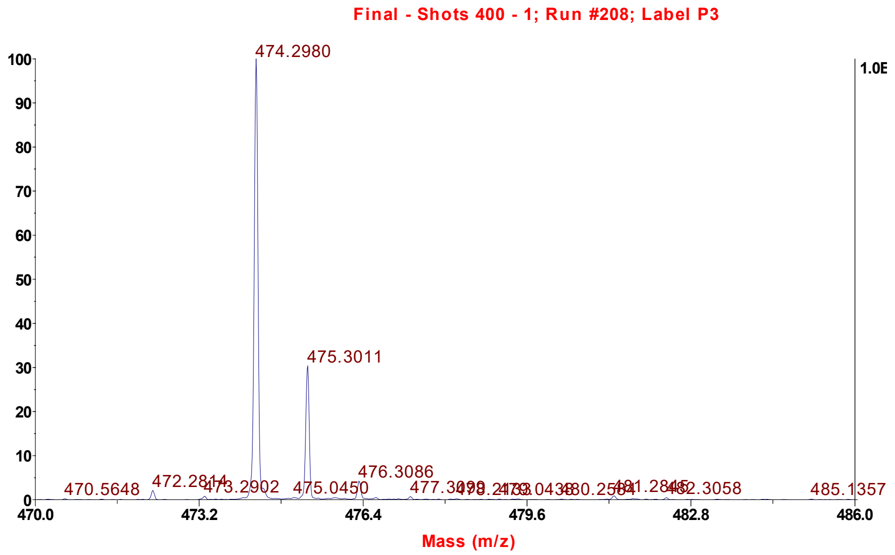


Figure S25. Mass spectar of **6** in the area m/z 99 – 1004 (upper spectrum) and its enlarged view in the area m/z 470 – 486 (lower spectrum).

**7**

Molecular formula: $C_{24}H_{33}N_3O_5$

Theoretical monoisotropic mass $[M+K]^+$: 482.2057

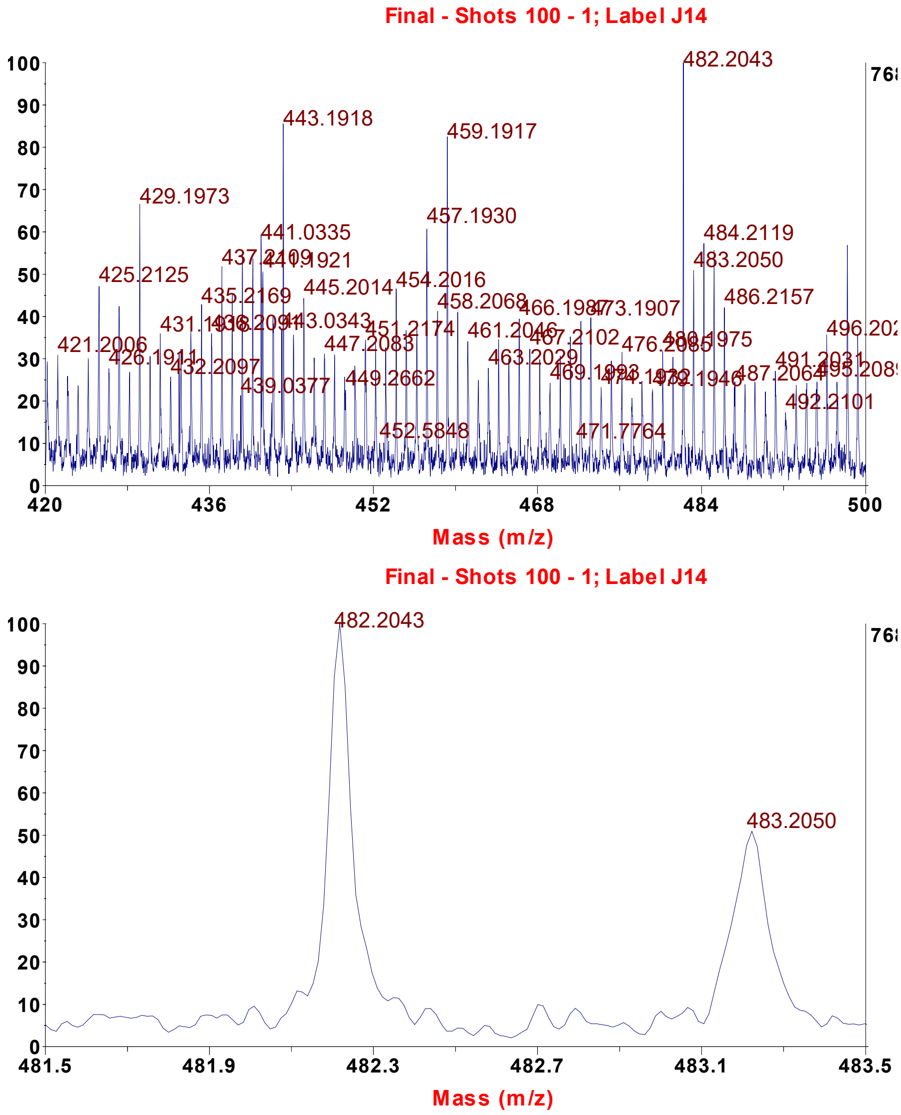


Figure S26. Mass spectar of **7** in the area m/z 420 – 500 (upper spectrum) and its enlarged view in the area m/z 481.5 – 483.5 (lower spectrum).

**8**

Molecular formula: $C_{23}H_{39}N_3O_5$

Theoretical monoisotropic mass $[M+H]^+$: 438.2968

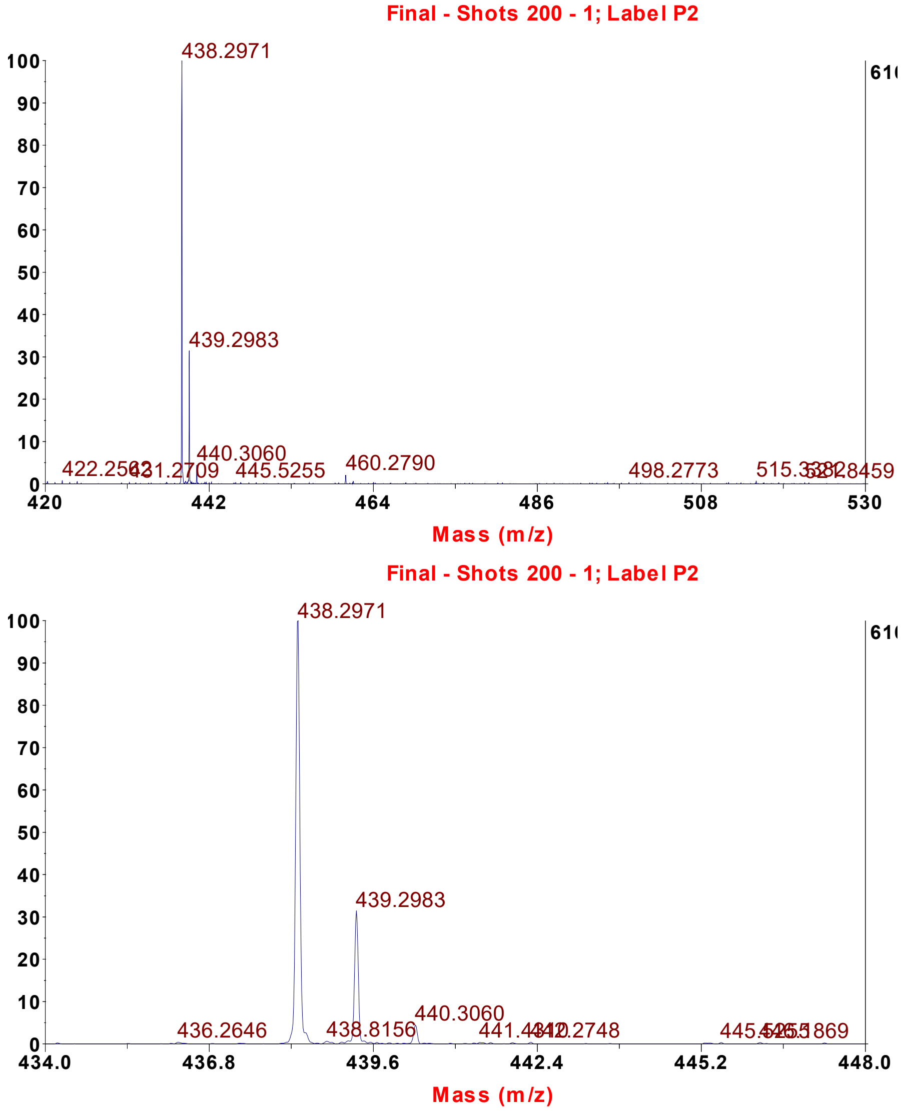


Figure S27. Mass spectar of **8** in the area m/z 420 – 530 (upper spectrum) and its enlarged view in the area m/z 434 – 448 (lower spectrum).

**9**

Molecular formula: $C_{28}H_{43}N_3O_5$

Theoretical monoisotropic mass $[M+H]^+$: 502.3281

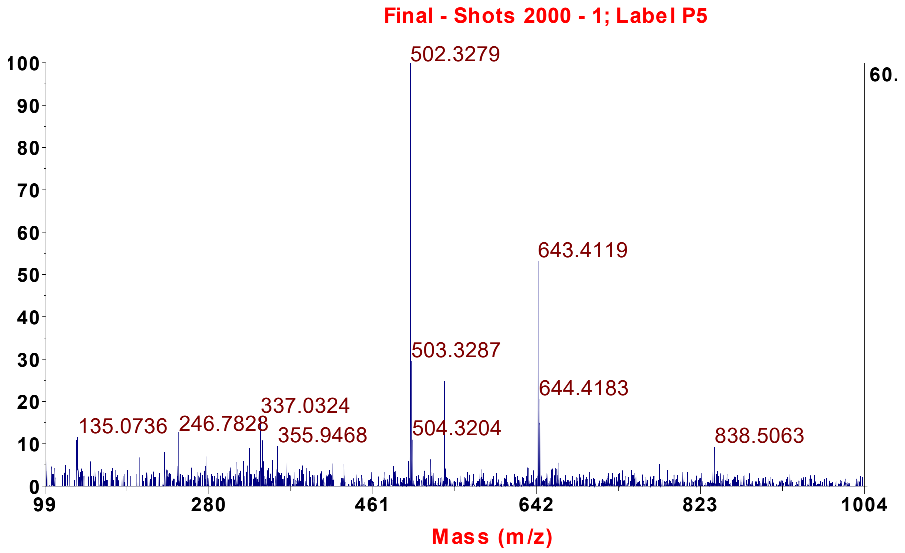


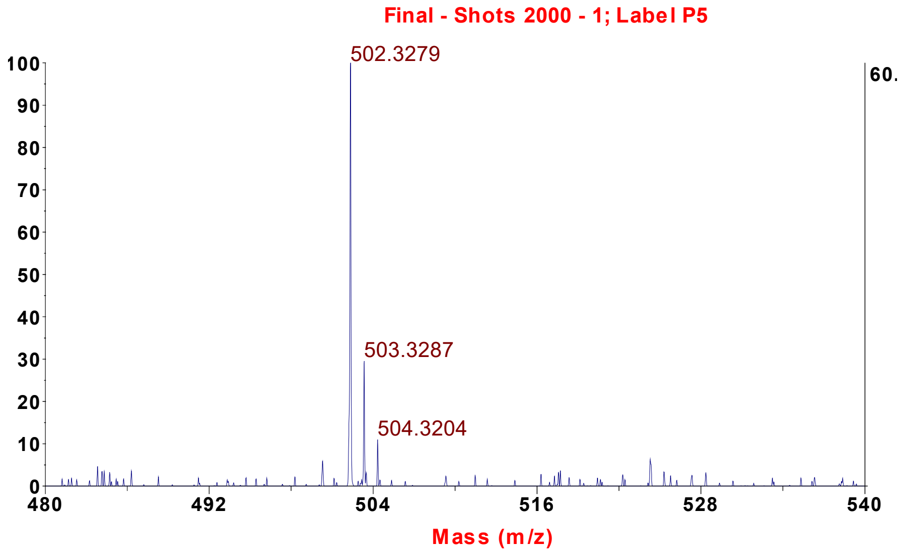


Figure S28. Mass spectar of **9** in the area m/z 99 – 1004 (upper spectrum) and its enlarged view in the area m/z 480 – 540 (lower spectrum).

**10**

Molecular formula: $C_{26}H_{37}N_3O_5$

Theoretical monoisotropic mass [M+H]$^+$: 472.2811

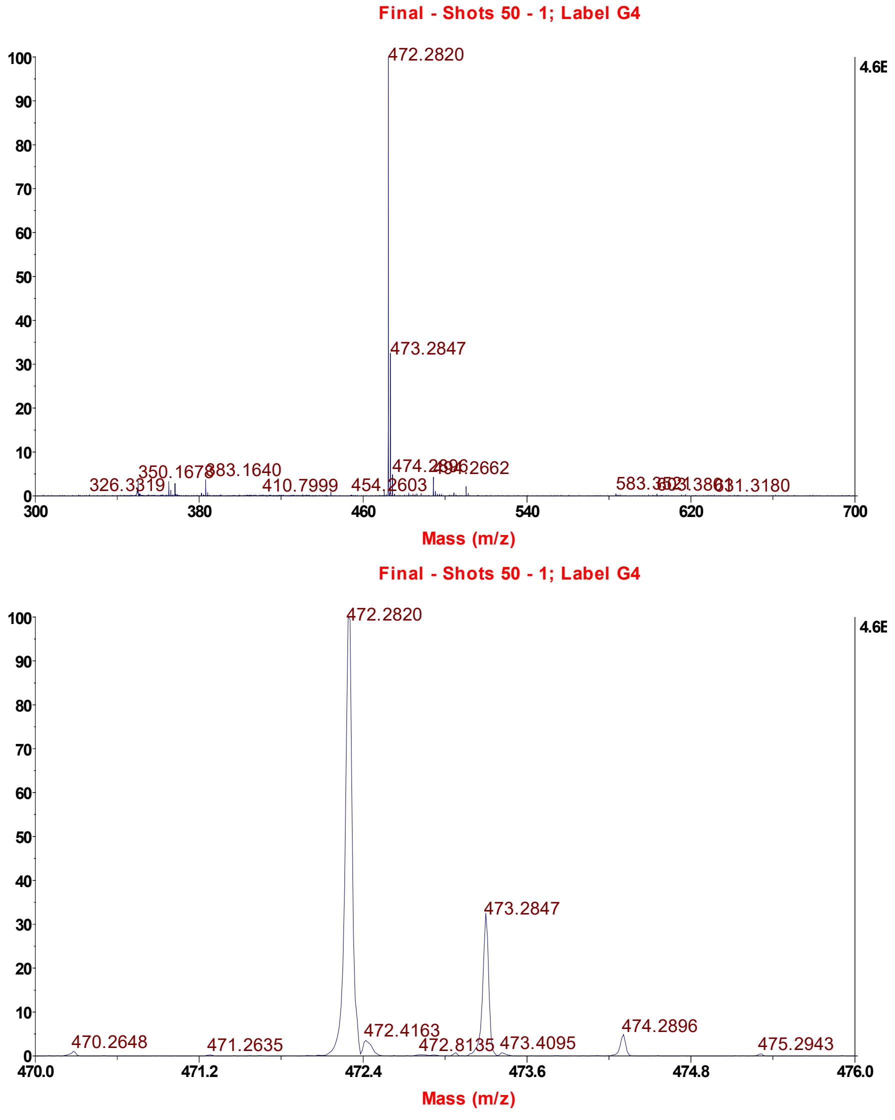


Figure S29. Mass spectar of **10** in the area m/z 300 – 700 (upper spectrum) and its enlarged view in the area m/z 470 – 476 (lower spectrum).

## 11

Molecular formula: $C_{26}H_{37}N_3O_5$

Theoretical monoisotopic mass [M+H]$^+$: 472.2811

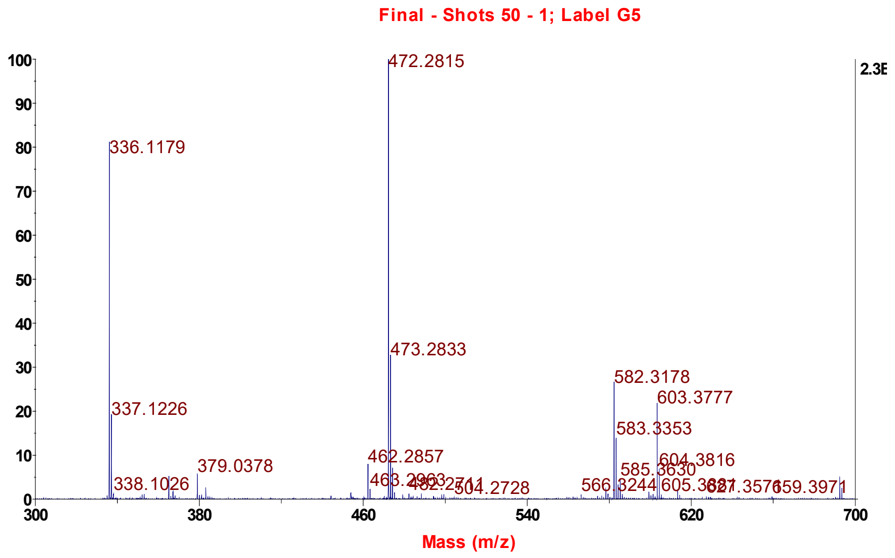


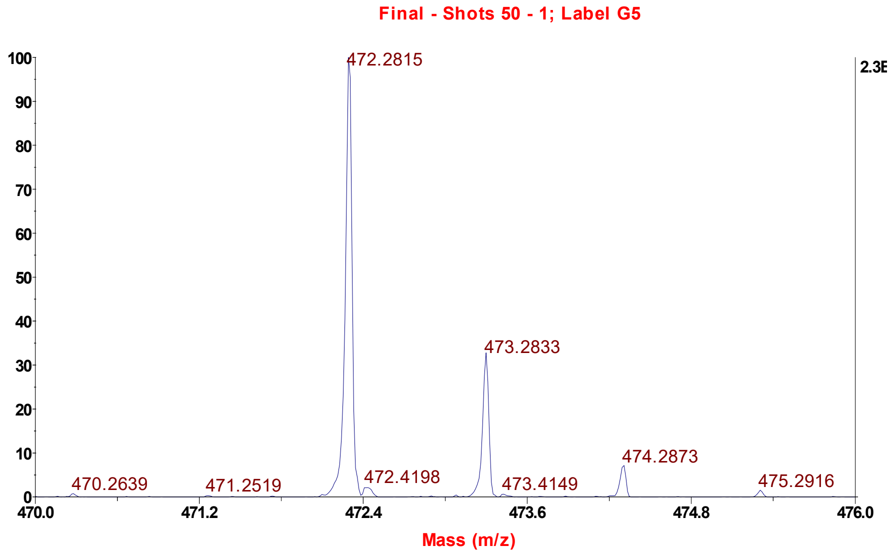


Figure S30. Mass spectar of **11** in the area m/z 300 – 700 (upper spectrum) and its enlarged view in the area m/z 470 – 476 (lower spectrum).

**12**

Molecular formula: $C_{24}H_{36}N_3O_5$

Theoretical monoisotropic mass $[M+H]^+$: 446.2655

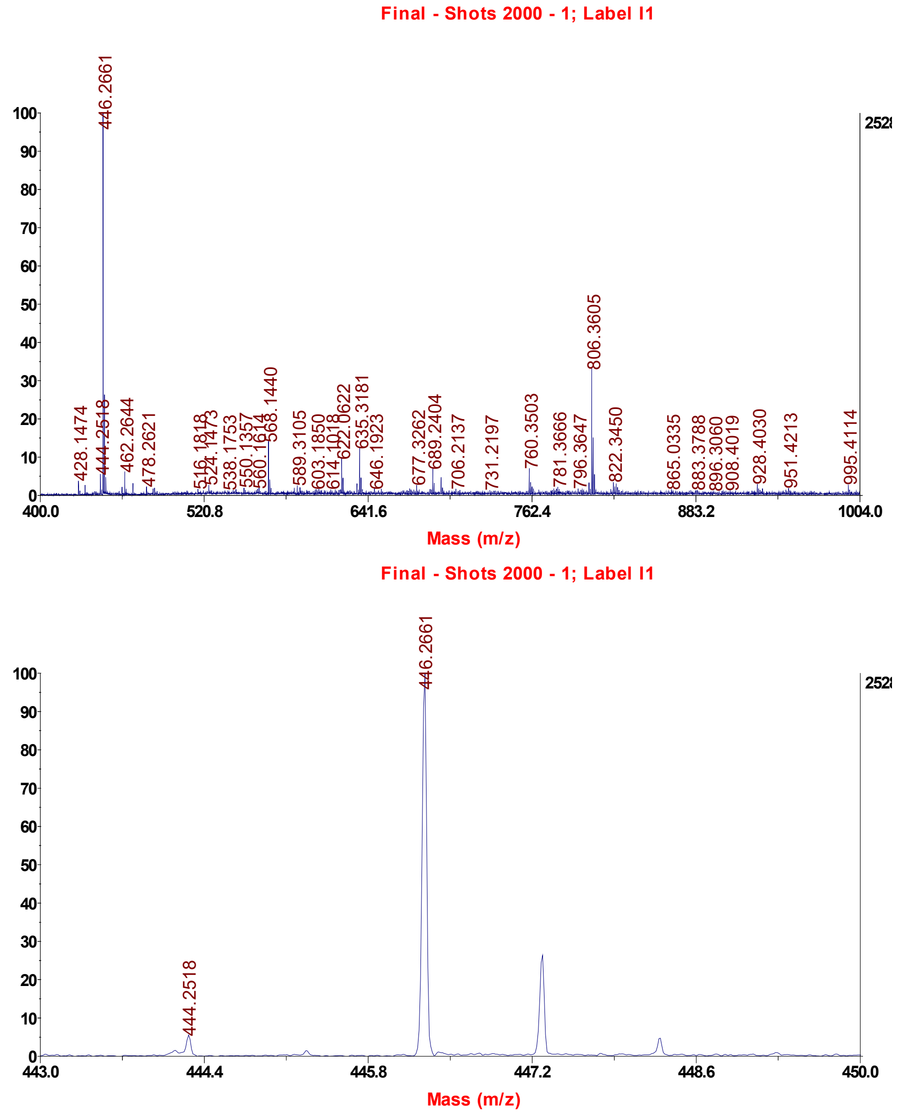


Figure S31. Mass spectar of **12** in the area m/z 400 – 1004 (upper spectrum) and its enlarged view in the area m/z 443 – 450 (lower spectrum).

**13**

Molecular formula: $C_{23}H_{36}ClN_3O_5$

Theoretical monoisotropic mass $[M+H]^+$: 434.2655

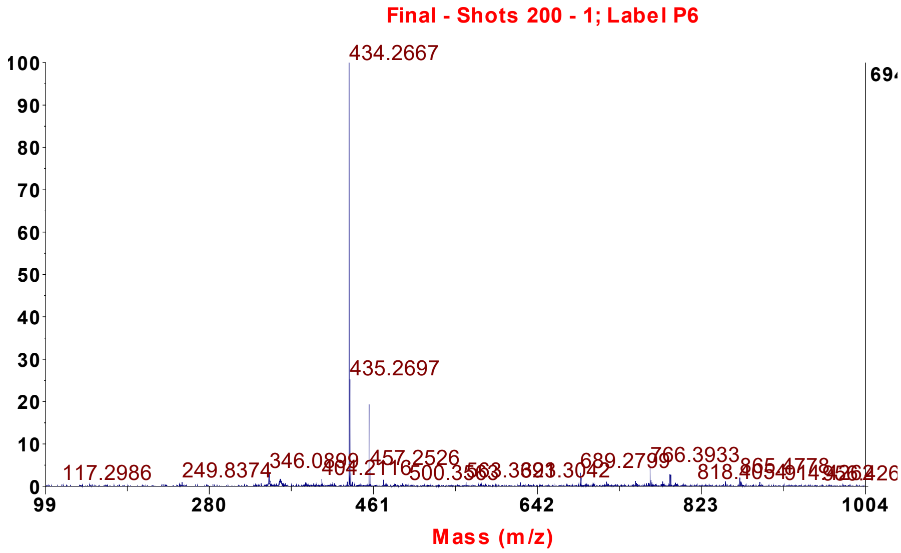


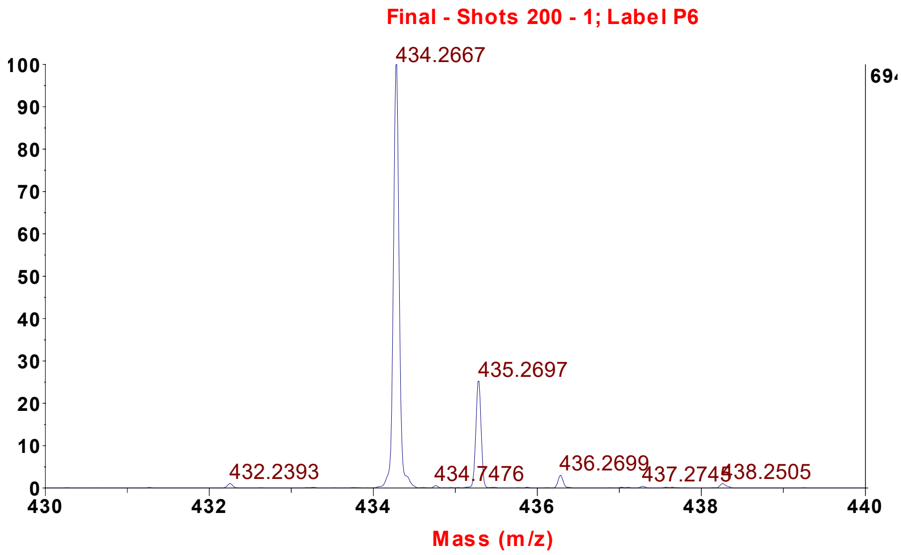


Figure S32. Mass spectar of **13** in the area m/z 99 – 1004 (upper spectrum) and its enlarged view in the area m/z 430– 440 (lower spectrum).

**14**

Molecular formula: $C_{28}H_{39}ClN_3O_5$

Theoretical monoisotropic mass [M+H]$^+$: 498.2968

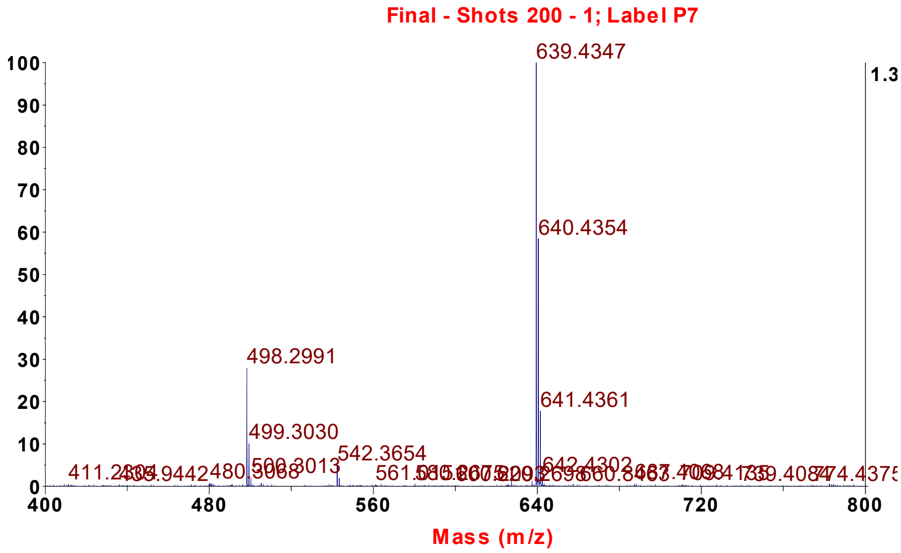


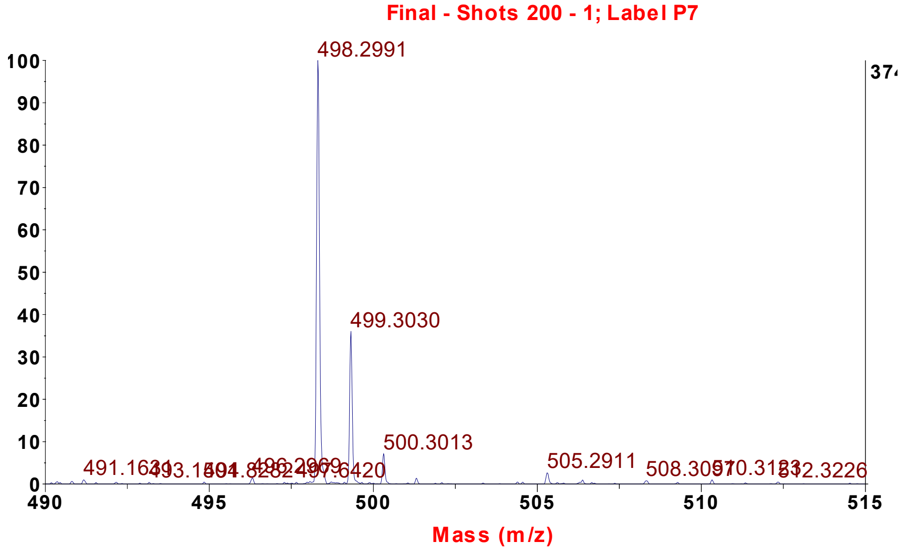


Figure S33. Mass spectar of **14** in the area m/z 400 – 800 (upper spectrum) and its enlarged view in the area m/z 490 – 515 (lower spectrum).

**15**

Molecular formula: $C_{26}H_{39}N_3O_5$

Theoretical monoisotropic mass [M+H]$^+$: 474.2968

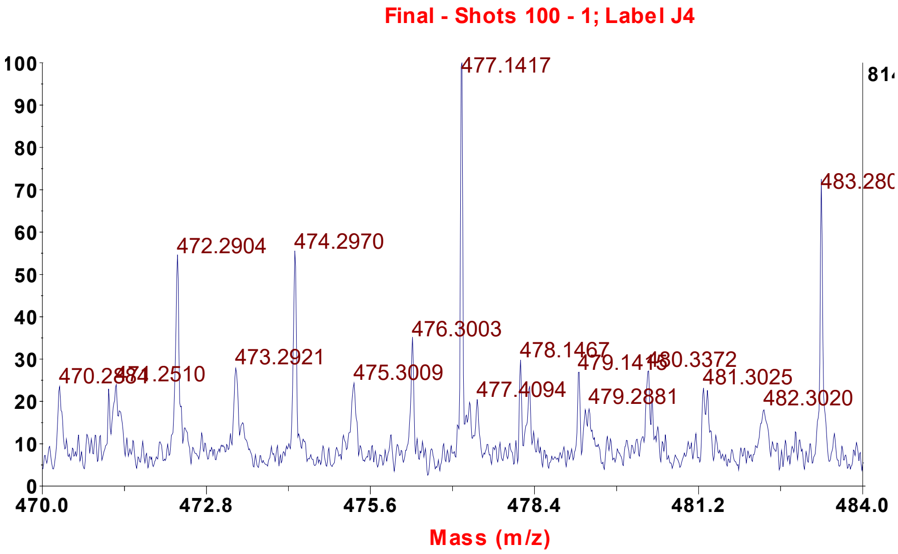


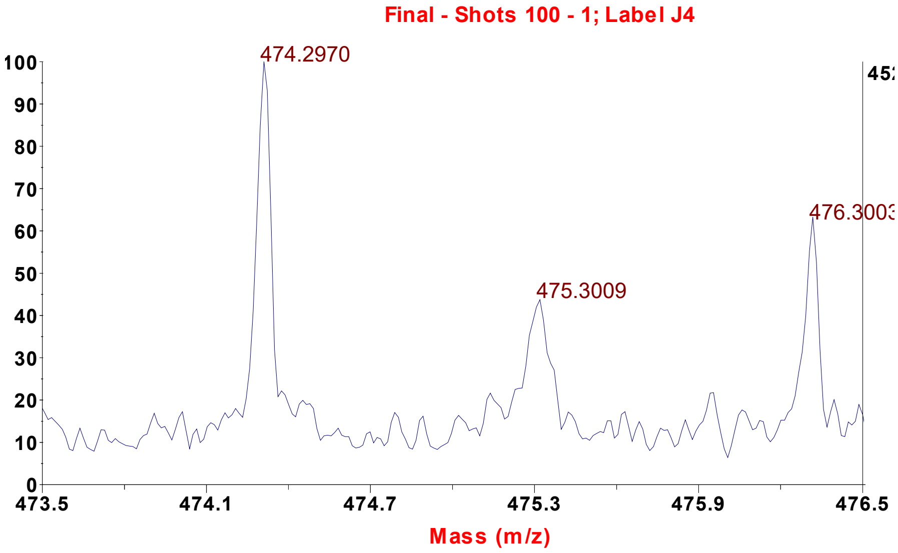


Figure S34. Mass spectar of **15** in the area m/z 470 – 484 (upper spectrum) and its enlarged view in the area m/z 473.5 – 476.5 (lower spectrum).

## 16

Molecular formula: $C_{25}H_{37}N_3O_5$

Theoretical monoisotropic mass $[M+H]^+$: 460.2811

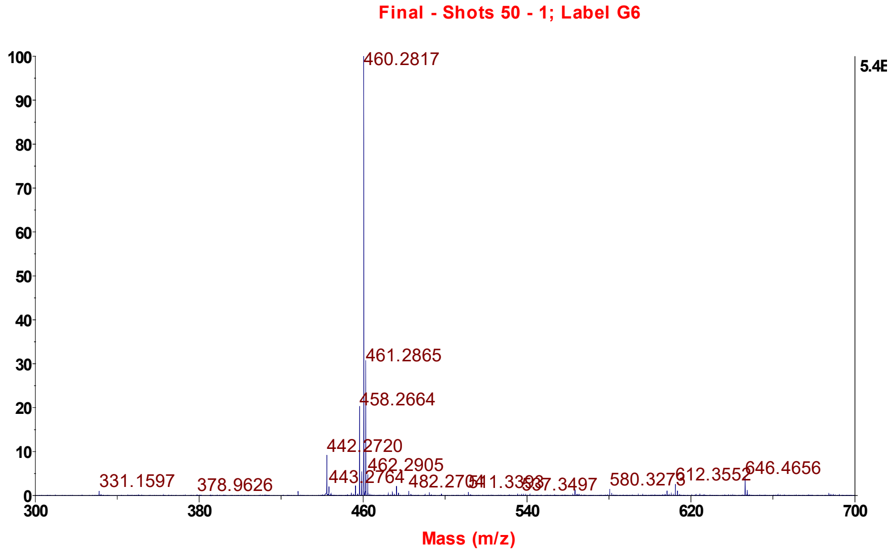


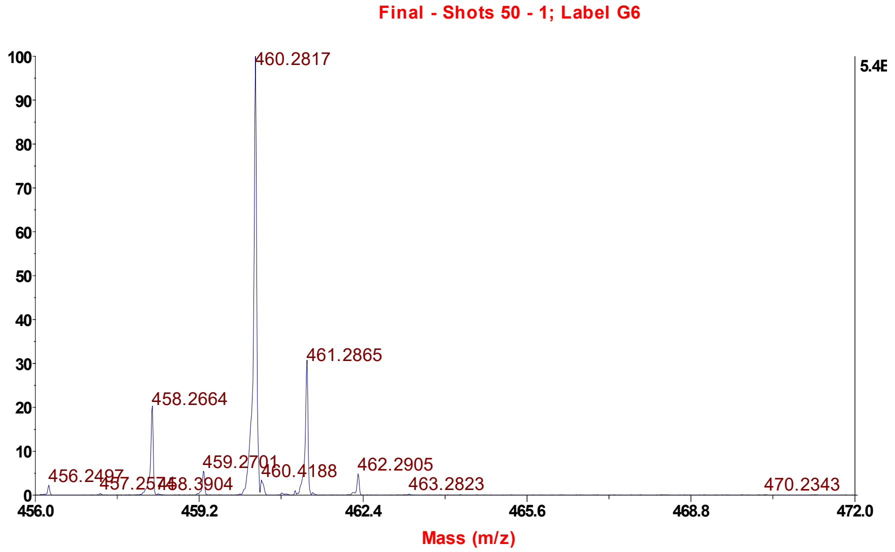


Figure S35. Mass spectar of **16** in the area m/z 300 – 700 (upper spectrum) and its enlarged view in the area m/z 456– 472 (lower spectrum).

**17**

Molecular formula: $C_{30}H_{43}N_3O_5$

Theoretical monoisotropic mass $[M+H]^+$: 526.3281

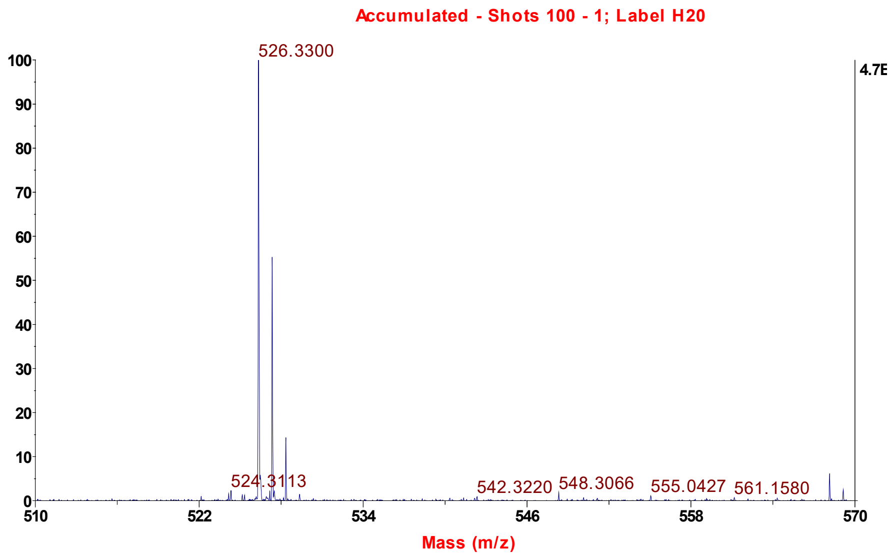


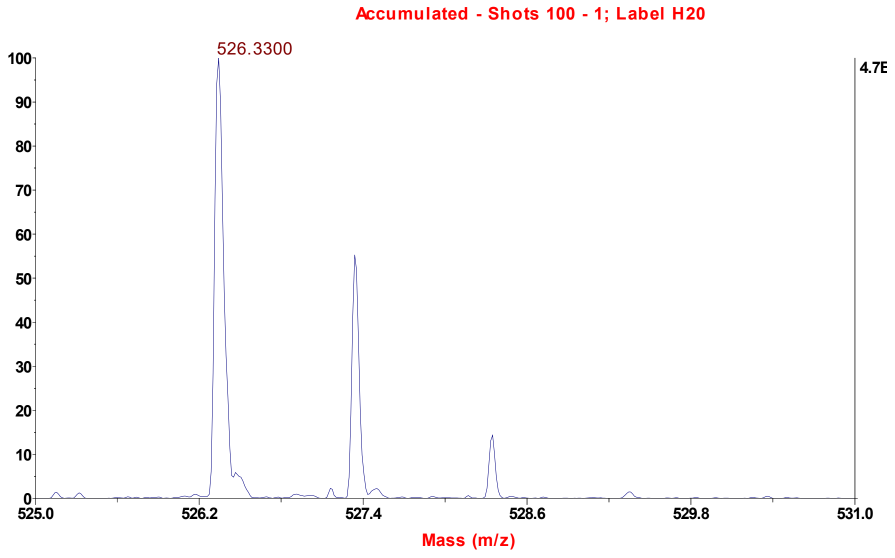


Figure S36. Mass spectar of **17** in the area m/z 510 – 570 (upper spectrum) and its enlarged view in the area m/z 525 – 531 (lower spectrum).

**18**

Molecular formula: $C_{30}H_{35}N_3O_5$

Theoretical monoisotropic mass $[M+H]^+$: 518.2655

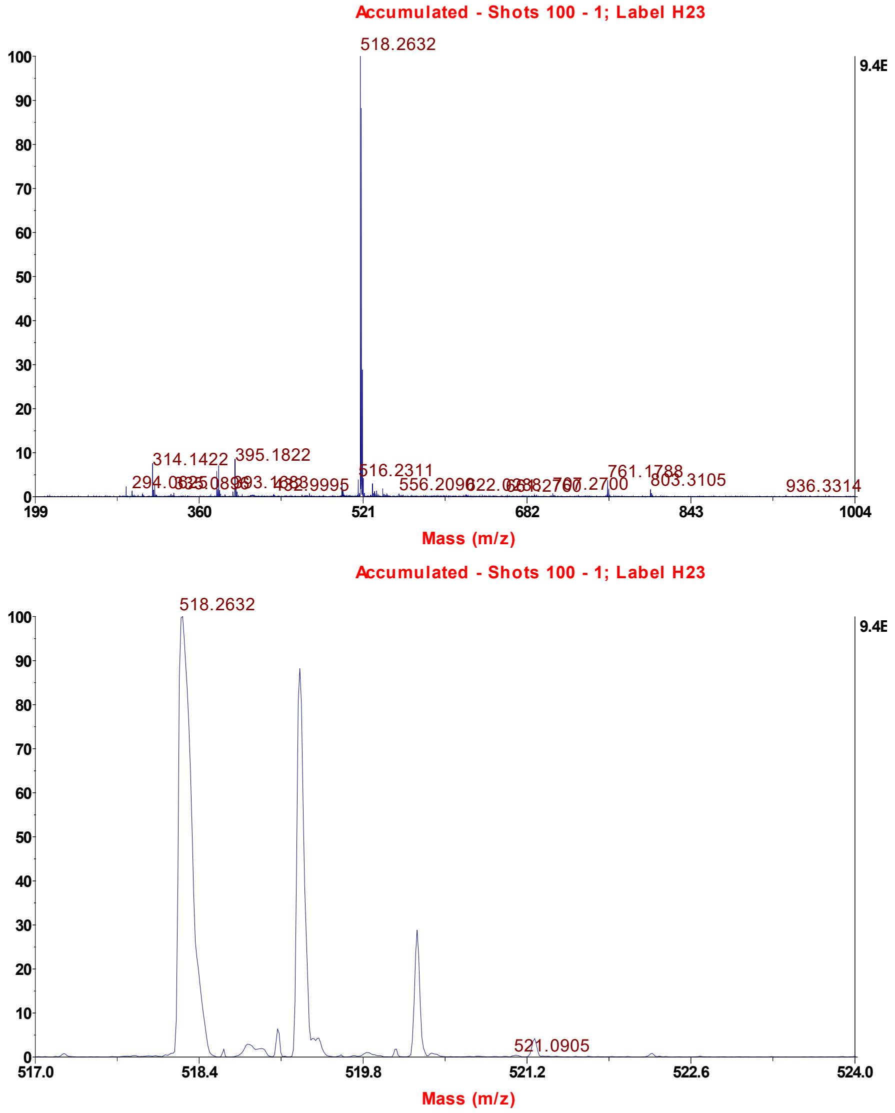


Figure S37. Mass spectar of **18** in the area m/z 199 – 1004 (upper spectrum) and its enlarged view in the area m/z 517 – 524 (lower spectrum).

List of predicted non-bonding interactions between hBChE and biscarbamates

**Table S3.** Interactions between compound **1** and BChE.

| Amino acid | Non-bonding interactions |
|---|---|
| Ser198 | O17, hydrogen bond-conventional hydrogen bond |
| Pro285 | H36, hydrogen bond-conventional hydrogen bond |
| Gln119 | H37, hydrogen bond-conventional hydrogen bond |
| His438 | H48, hydrogen bond-conventional hydrogen bond |
| Gly117 | O8, hydrogen bond-carbon hydrogen bond |
| His438 | O10, hydrogen bond-carbon hydrogen bond |
| Gln119 | H34, hydrogen bond-carbon hydrogen bond |
| Gln119 | H38, hydrogen bond-carbon hydrogen bond |
| Glu197 | H50, hydrogen bond-carbon hydrogen bond |
| Leu286 | H55, hydrogen bond-carbon hydrogen bond |
| Leu286 | H57, hydrogen bond-carbon hydrogen bond |
| Phe329 | 2DM, hydrophobic-π-π T-shaped |
| Leu286 | C27, hydrophobic-alkyl |
| Val288 | C27, hydrophobic-alkyl |
| Leu286 | C28, hydrophobic-alkyl |
| Trp82 | C25, hydrophobic-π-alkyl |
| Trp82 | C26, hydrophobic-π-alkyl |
| Trp82 | C25, hydrophobic-π-alkyl |
| Trp82 | C26, hydrophobic-π-alkyl |
| Trp231 | C27, hydrophobic-π-alkyl |
| Trp231 | C28, hydrophobic-π-alkyl |
| Trp231 | C27, hydrophobic-π-alkyl |
| Trp231 | C28, hydrophobic-π-alkyl |
| Phe398 | C28, hydrophobic-π-alkyl |
| His438 | C25, hydrophobic-π-alkyl |

**Table S4.** Interactions between compound **2** and BChE.

| Amino acid | Non-bonding interactions |
|---|---|
| HOH709 | O13, hydrogen bond-water hydrogen bond; conventional hydrogen bond |
| HOH706 | H47, hydrogen bond-water hydrogen bond; carbon hydrogen bond |
| Gln119 | O17, hydrogen bond-conventional hydrogen bond |
| Gln119 | H32, hydrogen bond-conventional hydrogen bond |
| Gly117 | O10, hydrogen bond-carbon hydrogen bond |
| Ser198 | H47, hydrogen bond-carbon hydrogen bond |
| Trp231 | H45, hydrophobic-π-sigma |
| Leu286 | C21, hydrophobic-alkyl |
| Trp82 | C25, hydrophobic-π-alkyl |
| Trp82 | C25, hydrophobic-π-alkyl |
| Trp231 | C22, hydrophobic-π-alkyl |
| Phe329 | C21, hydrophobic-π-alkyl |
| Phe398 | C22, hydrophobic-π-alkyl |
| His438 | C25, hydrophobic-π-alkyl |

**Table S5.** Interactions between compound **3** and BChE.

| Amino acid | Non-bonding interactions |
|---|---|
| HOH709 | O13, hydrogen bond-water hydrogen bond; conventional hydrogen bond |
| Ser287 | O17, hydrogen bond-conventional hydrogen bond |
| Gly117 | O7, hydrogen bond-carbon hydrogen bond |
| Ser198 | H59, hydrogen bond-carbon hydrogen bond |
| Leu286 | H61, hydrogen bond-carbon hydrogen bond |
| Val288 | C30, hydrophobic-alkyl |
| Trp82 | 7DM, hydrophobic-π-alkyl |
| Trp82 | 7DM, hydrophobic-π-alkyl |
| Trp231 | C29, hydrophobic-π-alkyl |
| Trp231 | C30, hydrophobic-π-alkyl |
| Trp231 | C29, hydrophobic-π-alkyl |
| Trp231 | C30, hydrophobic-π-alkyl |
| Phe398 | C29, hydrophobic-π-alkyl |
| His438 | 7DM, hydrophobic-π-alkyl |

**Table S6.** Interactions between compound **4** and BChE.

| Amino acid | Non-bonding interactions |
|---|---|
| HOH930 | O17, hydrogen bond-water hydrogen bond; conventional hydrogen bond |
| HOH825 | H38, hydrogen bond-water hydrogen bond; conventional hydrogen bond |
| HOH706 | H51, hydrogen bond-water hydrogen bond; carbon hydrogen bond |
| HOH1031 | H66, hydrogen bond-water hydrogen bond; carbon hydrogen bond |
| Gln119 | O8, hydrogen bond-conventional hydrogen bond |
| Asp70 | H35, hydrogen bond-conventional hydrogen bond |
| Pro285 | O17, hydrogen bond-carbon hydrogen bond |
| Leu286 | O7, hydrogen bond-carbon hydrogen bond |
| Leu286 | H56, hydrogen bond-carbon hydrogen bond |
| Leu286 | H57, hydrogen bond-carbon hydrogen bond |
| Leu286 | C27, hydrophobic-alkyl |
| Val288 | C27, hydrophobic-alkyl |
| Trp82 | 2EN, hydrophobic-π-alkyl |
| Trp82 | 2EN, hydrophobic-π-alkyl |
| Trp231 | C27, hydrophobic-π-alkyl |
| Trp231 | C27, hydrophobic-π-alkyl |
| Phe329 | C26, hydrophobic-π-alkyl |
| Phe398 | C26, hydrophobic-π-alkyl |
| His438 | C26, hydrophobic-π-alkyl |

**Table S7.** Interactions between compound **5** and BChE.

| Amino acid | Non-bonding interactions |
|---|---|
| HOH736 | O13, hydrogen bond-water hydrogen bond; conventional hydrogen bond |
| HOH910 | O10, hydrogen bond-water hydrogen bond; conventional hydrogen bond |
| HOH825 | H33, hydrogen bond-water hydrogen bond; carbon hydrogen bond |
| HOH910 | H34, hydrogen bond-water hydrogen bond; conventional hydrogen bond |
| Gly117 | O17, hydrogen bond-carbon hydrogen bond |
| His438 | H40, hydrogen bond-carbon hydrogen bond |
| Trp82 | N15, electrostatic-π-cation |
| Leu286 | C20, hydrophobic-alkyl |
| Trp82 | C29, hydrophobic-π-alkyl |
| Trp82 | C29, hydrophobic-π-alkyl |
| Trp231 | C20, hydrophobic-π-alkyl |
| Trp231 | C20, hydrophobic-π-alkyl |
| Phe329 | C19, hydrophobic-π-alkyl |
| Phe398 | C19, hydrophobic-π-alkyl |
| His438 | C19, hydrophobic-π-alkyl |

**Table S8.** Interactions between compound **6** and BChE.

| Amino acid | Non-bonding interactions |
|---|---|
| HOH786 | O17, hydrogen bond-water hydrogen bond; conventional hydrogen bond |
| HOH910 | O13, hydrogen bond-water hydrogen bond; conventional hydrogen bond |
| HOH989 | H43, hydrogen bond-water hydrogen bond; conventional hydrogen bond |
| HOH706 | H64, hydrogen bond-water hydrogen bond; carbon hydrogen bond |
| Gly116 | H39, hydrogen bond-conventional hydrogen bond |
| Leu286 | H59, hydrogen bond-carbon hydrogen bond |
| Glu197 | H69, hydrogen bond-carbon hydrogen bond |
| Gly116;Gly117 | 7EN, hydrophobic-amide-π stacked |
| Leu286 | C30, hydrophobic-alkyl |
| Val288 | C30, hydrophobic-alkyl |
| Trp82 | C32, hydrophobic-π-alkyl |
| Trp82 | C32, hydrophobic-π-alkyl |
| Trp82 | C33, hydrophobic-π-alkyl |
| Trp231 | C31, hydrophobic-π-alkyl |
| Trp231 | C31, hydrophobic-π-alkyl |
| His438 | C32, hydrophobic-π-alkyl |

**Table S9.** Interactions between compound **7** and BChE.

| Amino acid | Non-bonding interactions |
|---|---|
| Asp70 | H40, hydrogen bond; electrostatic-salt bridge; attractive charge |
| HOH825 | H37, hydrogen bond-water hydrogen bond; conventional hydrogen bond |
| HOH806 | H40, hydrogen bond-water hydrogen bond; conventional hydrogen bond |
| HOH930 | H41, hydrogen bond-water hydrogen bond; conventional hydrogen bond |
| HOH989 | H42, hydrogen bond-water hydrogen bond; carbon hydrogen bond |
| HOH708 | H59, hydrogen bond-water hydrogen bond; carbon hydrogen bond |
| Gln119 | O17, hydrogen bond-conventional hydrogen bond |
| Gly116 | O7, hydrogen bond-carbon hydrogen bond |
| Gly117 | O17, hydrogen bond-carbon hydrogen bond |
| His438 | O10, hydrogen bond-carbon hydrogen bond |
| Asp70 | H39, hydrogen bond-carbon hydrogen bond |
| Leu286 | H51, hydrogen bond-carbon hydrogen bond |
| Leu286 | H53, hydrogen bond-carbon hydrogen bond |
| Glu197 | H59, hydrogen bond-carbon hydrogen bond |
| Glu197 | H60, hydrogen bond-carbon hydrogen bond |
| Tyr332 | N15, electrostatic-π-cation |
| Leu286 | C27, hydrophobic-alkyl |
| Val288 | C27, hydrophobic-alkyl |
| Trp82 | C31, hydrophobic-π-alkyl |
| Trp82 | C32, hydrophobic-π-alkyl |
| Trp82 | C31, hydrophobic-π-alkyl |
| Trp82 | C32, hydrophobic-π-alkyl |
| Trp231 | C27, hydrophobic-π-alkyl |
| Trp231 | C28, hydrophobic-π-alkyl |
| Trp231 | C27, hydrophobic-π-alkyl |
| Trp231 | C28, hydrophobic-π-alkyl |
| Phe398 | C28, hydrophobic-π-alkyl |

**Table S10.** Interactions between compound **8** and BChE.

| Amino acid | Non-bonding interactions |
|---|---|
| HOH736 | O13, hydrogen bond-water hydrogen bond; conventional hydrogen bond |
| HOH910 | H35, hydrogen bond-water hydrogen bond; carbon hydrogen bond |
| Gly117 | O17, hydrogen bond-carbon hydrogen bond |
| Ser287 | H51, hydrogen bond-carbon hydrogen bond |
| Trp82 | N15, electrostatic-π-cation |
| Trp82 | H68, hydrophobic-π-sigma |
| Leu286 | C20, hydrophobic-alkyl |
| Val288 | C20, hydrophobic-alkyl |
| Ser287 | H66, hydrogen bond-carbon hydrogen bond |
| Trp82 | C31, hydrophobic-π-alkyl |
| Trp82 | C31, hydrophobic-π-alkyl |
| Trp231 | C20, hydrophobic-π-alkyl |
| Trp231 | C20, hydrophobic-π-alkyl |
| Phe329 | C21, hydrophobic-π-alkyl |
| His438 | C21, hydrophobic-π-alkyl |

**Table S11.** Interactions between compound **9** and BChE.

| Amino acid | Non-bonding interactions |
|---|---|
| HOH930 | O17, hydrogen bond-water hydrogen bond; conventional hydrogen bond |
| HOH709 | O13, hydrogen bond-water hydrogen bond; conventional hydrogen bond |
| HOH706 | H61, hydrogen bond-water hydrogen bond; carbon hydrogen bond |
| Asp70 | H36, hydrogen bond-conventional hydrogen bond |
| Leu286 | H70, hydrogen bond-carbon hydrogen bond |
| Leu286 | C31, hydrophobic-alkyl |
| Trp82 | 7DE, hydrophobic-π-alkyl |
| Trp82 | 7DE, hydrophobic-π-alkyl |
| Trp231 | C30, hydrophobic-π-alkyl |
| Trp231 | C30, hydrophobic-π-alkyl |
| Trp231 | C31, hydrophobic-π-alkyl |
| Trp231 | C31, hydrophobic-π-alkyl |
| Phe398 | C30, hydrophobic-π-alkyl |
| His438 | 7DE, hydrophobic-π-alkyl |
| His438 | C30, hydrophobic-π-alkyl |

**Table S12.** Interactions between compound **10** and BChE.

| Amino acid | Non-bonding interactions |
|---|---|
| HOH825 | H39, hydrogen bond-water hydrogen bond; conventional hydrogen bond |
| HOH706 | H62, hydrogen bond-water hydrogen bond; carbon hydrogen bond |
| Leu286 | C33, hydrophobic-alkyl |
| Val288 | C33, hydrophobic-alkyl |
| Trp231 | C33, hydrophobic-π-alkyl |
| Trp231 | C33, hydrophobic-π-alkyl |
| Phe329 | C32, hydrophobic-π-alkyl |
| Phe398 | C32, hydrophobic-π-alkyl |
| His438 | C32, hydrophobic-π-alkyl |

**Table S13.** Interactions between compound **11** and BChE.

| Amino acid | Non-bonding interactions |
|---|---|
| HOH709 | O17, hydrogen bond-water hydrogen bond; conventional hydrogen bond |
| HOH825 | H61, hydrogen bond-water hydrogen bond; carbon hydrogen bond |
| HOH706 | H63, hydrogen bond-water hydrogen bond; carbon hydrogen bond |
| Ser287 | O13, hydrogen bond-conventional hydrogen bond |
| Pro285 | H39, hydrogen bond-conventional hydrogen bond |
| Gln119 | H40, hydrogen bond-carbon hydrogen bond |
| Leu286 | H72, hydrogen bond-carbon hydrogen bond |
| Ala328 | C28, hydrophobic-alkyl |
| Leu286 | C33, hydrophobic-alkyl |
| Val288 | C33, hydrophobic-alkyl |
| Trp231 | C32, hydrophobic-π-alkyl |
| Trp231 | C33, hydrophobic-π-alkyl |
| Phe329 | C28, hydrophobic-π-alkyl |
| Phe329 | C29, hydrophobic-π-alkyl |
| Tyr332 | C28, hydrophobic-π-alkyl |

| | |
|---|---|
| Phe398 | C32, hydrophobic-π-alkyl |
| His438 | C29, hydrophobic-π-alkyl |
| His438 | C32, hydrophobic-π-alkyl |

**Table S14.** Interactions between compound **12** and BChE.

| Amino acid | Non-bonding interactions |
|---|---|
| Asp70 | H41, hydrogen bond; electrostatic-salt bridge; attractive charge |
| HOH930 | O10, hydrogen bond-water hydrogen bond; conventional hydrogen bond |
| HOH930 | H37, hydrogen bond-water hydrogen bond; conventional hydrogen bond |
| HOH825 | H42, hydrogen bond-water hydrogen bond; carbon hydrogen bond |
| HOH706 | H62, hydrogen bond-water hydrogen bond; carbon hydrogen bond |
| Gln119 | O7, hydrogen bond-conventional hydrogen bond |
| Pro285 | O10, hydrogen bond-carbon hydrogen bond |
| Asp70 | H36, hydrogen bond-carbon hydrogen bond |
| Asp70 | H38, hydrogen bond-carbon hydrogen bond |
| HOH930 | 2MF, other-π-lone pair |
| Ala277 | 2P, hydrophobic-alkyl |
| Leu286 | 2P, hydrophobic-alkyl |
| Trp82 | 2P, hydrophobic-π-alkyl |
| Trp82 | 2P, hydrophobic-π-alkyl |
| Trp231 | 2P, hydrophobic-π-alkyl |
| Trp231 | 2P, hydrophobic-π-alkyl |
| His438 | 2P, hydrophobic-π-alkyl |

**Table S15.** Interactions between compound **13** and BChE.

| Amino acid | Non-bonding interactions |
|---|---|
| Asp70 | N15, electrostatic-attractive charge |
| HOH736 | O8, hydrogen bond-water hydrogen bond; conventional hydrogen bond |
| HOH786 | O17, hydrogen bond-water hydrogen bond; conventional hydrogen bond |
| HOH709 | 6P, hydrogen bond-water hydrogen bond; π-donor hydrogen donor |
| HOH920 | H37, hydrogen bond-water hydrogen bond; carbon hydrogen bond |
| HOH989 | H38, hydrogen bond-water hydrogen bond; carbon hydrogen bond |
| HOH930 | H40, hydrogen bond-water hydrogen bond; conventional hydrogen bond |
| Asp70 | H36, hydrogen bond-conventional hydrogen bond |
| Asp70 | H35, hydrogen bond-carbon hydrogen bond |
| Gln119 | H38, hydrogen bond-carbon hydrogen bond |
| Leu286 | 6P, hydrophobic-alkyl |
| Trp82 | 6P, hydrophobic-π-alkyl |
| Trp82 | 6P, hydrophobic-π-alkyl |
| Trp231 | 6P, hydrophobic-π-alkyl |
| Trp231 | 6P, hydrophobic-π-alkyl |

**Table S16.** Interactions between compound **14** and BChE.

| Amino acid | Non-bonding interactions |
|---|---|
| HOH736 | O13, hydrogen bond-water hydrogen bond; conventional hydrogen bond |
| HOH930 | O17, hydrogen bond-water hydrogen bond; conventional hydrogen bond |
| HOH910 | H41, hydrogen bond-water hydrogen bond; conventional hydrogen bond |
| HOH706 | H62, hydrogen bond-water hydrogen bond; carbon hydrogen bond |
| Ser198 | H61, hydrogen bond-carbon hydrogen bond |
| Leu286 | H67, hydrogen bond-carbon hydrogen bond |
| Trp82 | N15, electrostatic-π-cation |
| Trp82 | H50, hydrophobic-π-sigma |
| Leu286 | 7P, hydrophobic-alkyl |
| Trp82 | 7P, hydrophobic-π-alkyl |
| Trp231 | 7P, hydrophobic-π-alkyl |
| Trp231 | 7P, hydrophobic-π-alkyl |
| His438 | 7P, hydrophobic-π-alkyl |

**Table S17.** Interactions between compound **15** and BChE.

| Amino acid | Non-bonding interactions |
|---|---|
| Asp70 | H38, hydrogen bond-carbon hydrogen bond |
| Gln119 | H63, hydrogen bond-carbon hydrogen bond |
| Leu286 | H65, hydrogen bond-carbon hydrogen bond |
| Leu286 | H66, hydrogen bond-carbon hydrogen bond |
| Trp82 | N15, electrostatic-π-cation |
| Leu286 | 2PI, hydrophobic-alkyl |
| Trp82 | 2PI, hydrophobic-alkyl |
| Trp231 | 2PI, hydrophobic-π-alkyl |
| Trp231 | 2PI, hydrophobic-π-alkyl |

**Table S18.** Interactions between compound **16** and BChE.

| Amino acid | Non-bonding interactions |
|---|---|
| HOH709 | O17, hydrogen bond-water hydrogen bond; conventional hydrogen bond |
| HOH1024 | H38, hydrogen bond-water hydrogen bond; conventional hydrogen bond |
| HOH825 | H43, hydrogen bond-water hydrogen bond; carbon hydrogen bond |
| HOH825 | H50, hydrogen bond-water hydrogen bond; carbon hydrogen bond |
| HOH706 | H61, hydrogen bond-water hydrogen bond; carbon hydrogen bond |
| Ala277 | C31, hydrophobic-alkyl |
| Leu286 | 5PI, hydrophobic-alkyl |
| Ala328 | 5PI, hydrophobic-alkyl |
| Trp231 | 5PI, hydrophobic-π-alkyl |
| Trp231 | 5PI, hydrophobic-π-alkyl |
| Phe329 | 5PI, hydrophobic-π-alkyl |
| Tyr332 | 5PI, hydrophobic-π-alkyl |

**Table S19.** Interactions between compound **17** and BChE.

| Amino acid | Non-bonding interactions |
|---|---|
| HOH910 | O7, hydrogen bond-water hydrogen bond; conventional hydrogen bond |
| HOH709 | O10, hydrogen bond-water hydrogen bond; carbon hydrogen bond |
| HOH825 | H71, hydrogen bond-water hydrogen bond; carbon hydrogen bond |
| Ser287 | H43, hydrogen bond-conventional hydrogen bond |
| Thr284 | H46, hydrogen bond-conventional hydrogen bond |
| Gly117 | O8, hydrogen bond-carbon hydrogen bond |
| Thr284 | H42, hydrogen bond-carbon hydrogen bond |
| Ser287 | H42, hydrogen bond-carbon hydrogen bond |
| Ser287 | H42, hydrogen bond-carbon hydrogen bond |
| Asp70 | H64, hydrogen bond-carbon hydrogen bond |
| Leu286 | H81, hydrogen bond-carbon hydrogen bond |
| Ala199 | 7PI, hydrophobic-alkyl |
| Ala328 | 7PI, hydrophobic-alkyl |
| Trp231 | 7PI, hydrophobic-π-alkyl |
| Trp231 | 7PI, hydrophobic-π-alkyl |
| Phe329 | 7PI, hydrophobic-π-alkyl |
| Tyr332 | 7PI, hydrophobic-π-alkyl |

**Table S20.** Interactions between compound **18** and BChE.

| Amino acid | Non-bonding interactions |
|---|---|
| HOH825 | H48, hydrogen bond-water hydrogen bond; carbon hydrogen bond |
| Leu286 | H52, hydrogen bond-carbon hydrogen bond |
| Ser287 | H53, hydrogen bond-carbon hydrogen bond |
| HOH930 | 2MF, other-π-lone pair |
| Tyr332 | O10, other-π-lone pair |
| Trp82 | 2MF, hydrophobic-π-π T-shaped |
| Trp231 | 2MF, hydrophobic-π-π T-shaped |
| Trp231 | 2MF, hydrophobic-π-π T-shaped |
| Tyr332 | 2MF, hydrophobic-π-π T-shaped |
| Pro285 | 2MF, hydrophobic-alkyl |
| Ala328 | C20, hydrophobic-alkyl |
| Val288 | C21, hydrophobic-alkyl |
| Phe329 | C20, hydrophobic-π-alkyl |
| Tyr332 | C20, hydrophobic-π-alkyl |
| Leu286 | 2MF, hydrophobic-π-alkyl |
